# Resurrecting tRNAs of the Last Universal Common Ancestor (Commonote) Toward Rebuilding the Ancient Translation System

**DOI:** 10.1007/s00239-026-10332-5

**Published:** 2026-07-09

**Authors:** Kei Yamanouchi, Hinata Moriya, Shin-ichi Yokobori

**Affiliations:** Department of Applied Life Sciences, School of Life Sciences, Tokyo University of Pharmacy and Life Sciences, Horinouchi, Tokyo, Hachioji, 192-0392 Japan

**Keywords:** tRNA, Ancestral sequence reconstruction, Last universal common ancestor, Wobble rule

## Abstract

Ancestral sequence reconstruction (ASR) was used to study the evolution of the translational system by inferring tRNA sequences of the Last Archaeal Common Ancestor (LACA), the Last Bacterial Common Ancestor (LBCA), and the Last Universal Common Ancestor (LUCA), referred to as “Commonote” in our previous studies. A composite phylogenetic tree inferred from the reconstructed ancestral tRNA sequences revealed a topology distinct from those of previously inferred aminoacyl-tRNA synthetase (ARS) trees. This incongruence may reflect changes in tRNA structural features or distinct evolutionary pressures on tRNAs and ARSs, suggesting a more nuanced evolutionary history of the translation system, but it may also arise from methodological differences between studies. Phylogenetic analysis identified conserved identity elements, such as the discriminator base and anticodon, across tRNAs for different amino acids, supporting their status as ancestral features. Most LUCA tRNA species retain bacterial identity elements, potentially enabling their use in modern systems such as *Escherichia coli*, although exceptions, including tRNA^Gly^ and initiator tRNA^Met,^ may present functional limitations. To evaluate the translational functionality of LUCA tRNAs and ancient codon-anticodon interactions, synthesized LUCA tRNA^Ala^ and tRNA^Ser^ variants with anticodons decoding ACN codons were tested in an *E. coli* reconstituted in vitro translation system. These reconstructed tRNAs successfully decoded ACN codons, with tRNA^Ala^ variants exhibiting translation efficiencies comparable to those of native *E. coli* tRNA^Thr^. Notably, decoding patterns differed from Crick’s and extended wobble rules, as anticodons with various first-position bases recognized multiple third codon positions. This study offers new insights into the early evolution of the translation system and demonstrates the functional potential of ancestral tRNAs in contemporary contexts.

**Supplementary Information:**

The online version contains supplementary material available at 10.1007/s00239-026-10332-5.

## Introduction

The inference of ancestral protein or nucleic acid sequences using molecular phylogenetic analysis, commonly referred to as ancestral sequence reconstruction (ASR), has become a widely used approach in evolutionary studies and is often followed by synthesis and functional characterization of the inferred molecules (Jermann et al. 1995; Gaucher et al. 2003, 2008; Finnigan et al. 2012; Kacar et al. 2017a, b). ASR is a powerful approach for reconstructing ancestral biomolecules and experimentally investigating the physiology of extinct organisms. For example, Gaucher et al. (2008) reconstructed the translational elongation factor EF-Tu from various evolutionary ages, analyzed its thermal stability, and used this information to infer that ancestral organisms were thermophilic, supporting hypotheses about elevated global temperatures in ancient environments. In the same manner, our laboratory analyzed the thermostability of an inferred ancestral nucleoside diphosphate kinase (NDK) and inferred that the common ancestor of Archaea and Bacteria—as well as the last universal common ancestor (LUCA, referred to as “Commonote” in our previous studies such as Furukawa et al. 2017)—were likely (hyper) thermophiles (Akanuma et al. 2013, 2015). We also inferred ancient global surface temperature trends based on the properties of ancestral NDKs (Garcia et al. 2017; Harada et al. 2019).

The translation systems of Archaea, Bacteria, and Eukarya generally utilize the standard genetic code, with a few exceptions (Watanabe and Yokobori 2014). Thus, the last archaeal common ancestor (LACA), last bacterial common ancestor (LBCA), and LUCA are all presumed to have used the standard code. During translation, specific amino acids are attached to their corresponding tRNAs by aminoacyl-tRNA synthetases (ARSs), and the anticodon of tRNAs pairs with the codon of mRNA on ribosomes, preserving the codon-amino acid relationship.

Prior to LUCA, the early translation system likely utilized a limited set of prebiotically synthesized amino acids, with the repertoire expanding over time (cf. Wong 1975). Functional proteins formed from such prebiotic amino acids—identified in meteorites, asteroids such as Ryugu (Lawless 1973; Cronin and Pizzarello 1983; Naraoka et al. 2023; Parker et al. 2023) and chemical evolution experiments such as those of Stanley Miller (Miller 1953)—have been studied experimentally (e.g. Patel et al. 2015; Shibue et al. 2018).

Regarding aminoacylation, it has been hypothesized that, in the RNA world, a limited set of amino acids was recognized and bound by proto-tRNAs (Koonin 2017). As proto-tRNAs diversified, different RNAs began to recognize similar amino acids, driving the emergence of polypeptide cofactors and protein domains that catalyzed aminoacylation, ultimately giving rise to the modern ARS system (Furukawa et al. 2022).

Despite their different amino acid specificities, all tRNAs share a conserved structure, suggesting that they are derived from a common ancestor. This structural conservation allows the reconstruction of composite phylogenetic trees of tRNAs. However, due to their short sequence length and limited phylogenetic signal, tRNA-based phylogenetic analysis remains challenging. In one such effort, de Farias (2013) inferred ancestral tRNA sequences and a composite phylogenetic tree but did not address the co-evolution of tRNAs and ARSs. However, this study emphasized the evolutionary significance of the second anticodon position. Other studies have investigated early tRNA evolution prior to the establishment of the canonical L-shaped (cloverleaf-like) structure (Tamura 2015; Root-Bernstein et al. 2016).

Phylogenetic studies of ARSs suggest that GlnRS originated from an archaeal/eukaryal-type GluRS (Woese et al. 2000; Nair et al. 2016). GlnRS forms a distinct clade that is closely related to archaeal and eukaryal GluRS, rather than bacterial GluRS. Similarly, AsnRS is thought to have evolved from archaeal AspRS (Woese et al. 2000; Nair et al. 2016). GluRS and AspRS exist in two forms: non-discriminating (ND-GluRS and ND-AspRS) and discriminating (D-GluRS and D-AspRS), based on their ability to distinguish between tRNA^Glu^ and tRNA^Gln^ or tRNA^Asp^ and tRNA^Asn^. These observations imply that LUCA may have lacked dedicated GlnRS and AsnRS enzymes, instead relying on ND-GluRS and ND-AspRS in combination with tRNA-dependent transamidosomes to synthesize Gln and Asn from Glu and Asp directly on tRNA^Gln^ and tRNA^Asn^.

In Bacteria, methionine-attached initiator tRNA is formylated (fMet-tRNA_i_^fMet^) by methionyl-tRNA formyltransferase, forming a complex with IF2-GTP to initiate translation. In contrast, archaeal and eukaryal initiator tRNAs (Met-tRNA_i_^Met^) are not formylated. Although formylation enhances bacterial translation initiation, it is not essential; disruption of the formyltransferase gene in *Escherichia coli* (*E. coli*) is non-lethal but reduces growth (Guillon et al. 1992a). Therefore, it is likely that Met-tRNA_i_^Met^ in LUCA was not formylated and lacked features for interaction with the methionyl-tRNA formyltransferase.

Another important aspect of tRNA evolution is codon decoding. While non-Watson–Crick base pairs (e.g., G: U) are tolerated at the wobble position (the first position of the tRNA anticodon and the third position of the mRNA codon) (Crick 1966), wobble rules vary among systems (Osawa et al. 1992; Watanabe and Yokobori 2011, 2014).

Unmodified U at the first anticodon position enables decoding of all four codons in a 4-codon box in translation systems with a limited tRNA repertoire, such as mycoplasmas and mitochondria (Osawa et al. 1992; Watanabe and Yokobori 2011, 2014). In contrast, in Eukarya, unmodified U typically pairs only with A. Modified U nucleotide, such as 5-carboxymethylaminomethyluridine (cmnm⁵U), 5-methylaminomethyluridine (mnm⁵U), or 2-thiouridine derivatives—are common in bacterial and eukaryal tRNAs, while archaeal systems tend to use simpler modifications (Masuda and Hou 2024). Modified U at the first anticodon position decodes only A and G at the third codon position.

Other unmodified nucleotides (A, G, and C) also appear at the first anticodon position in some modern tRNAs, enabling broader decoding. For instance, unmodified A is found in mycoplasma tRNAs and in the mitochondrial tRNA^Arg^_ACG_ from the nematode, *Ascaris suum*, which decodes all CGN codons (Watanabe et al. 1997). Inosine (I34), a deaminated A, expands decoding capacity in Bacteria (decoding U, C, and A) and Eukarya (decoding U and C) but is absent in Archaea (Masuda and Hou 2024). Unmodified G can decode C and U in general, but also A as seen in *Drosophila* mitochondrial tRNA^Ser^_GCU_ (Tomita et al. 1999). Queuosine (Q), a modified base found in bacterial and eukaryal tRNAs for Tyr, His, Asn, and Asp, is also absent in Archaea (Masuda and Hou 2024). Unmodified C can also function in decoding, although it decodes only G in Crick’s wobble rule, e.g., the mitochondrial tRNA^Met^_CAU_ decodes both AUG and AUA codons in the squid, *Loligo bleekeri* (Ohira et al. 2013). Modifications like lysidine (Bacteria) and agmatidine (Archaea) further diversify decoding (decoding A instead of G) (Muramatsu et al. 1988; Mandal et al. 2010; Ikeuchi et al. 2010).

Given the differences in modification between bacterial and archaeal tRNAs (in particular, A and C), it is plausible that the number of anticodon modifications in LUCA was likely minimal or absent. Estimating wobble rules in this pre-modification era is essential for understanding tRNA evolution. Watanabe and Yokobori (2014) proposed that 4-codon boxes were decoded by single tRNAs with unmodified U (or A) at the first anticodon position. For NNY 2-codon families, tRNAs with G_34_ are likely responsible, and for NNR families, U_34_ was hypothesized. U_34_ could potentially decode both NNY and NNR, possibly competing with G_34_ tRNAs. The roles of unmodified A and C remain to be clarified. A central unanswered question concerns the identity of the nucleotides that were used at the first anticodon position in the unmodified era and whether this minimum set was sufficient to decode all the sense codons.

As described above, ASR is widely used to reconstruct ancient genes and proteins and to infer ancient life and environments (Gaucher et al. 2003, 2008; Thornton 2004; Akanuma et al. 2015). Ancestral proteins from LACA, LBCA, and LUCA have been reconstructed and their properties inferred to evaluate the thermal condition of their host’s growth environment by several groups, including ours (Akanuma et al. 2013, 2015; Garcia et al. 2017; Harada et al. 2019).

ASR approaches have also been applied to study translational system evolution. For instance, ancestral EF-Tu proteins have been analyzed not only for their thermostability but also for their ability to function within extant protein synthesis systems (Gaucher et al. 2008; Kacar et al. 2017a, b). In the case of aminoacyl-tRNA synthetases (ARSs), Fournier et al. (2011) inferred the ancestral sequence of a common ancestor of IleRS and ValRS based on a composite phylogenetic tree of IleRS, ValRS, and LeuRS. Their analysis suggested that the ancestral enzyme could have discriminated between isoleucine and valine, implying that both amino acids were already utilized in protein synthesis at that evolutionary stage. Similarly, Fournier and Alm (2015) reconstructed the ancestral protein sequences of TyrRS and TrpRS and proposed that tryptophan was incorporated into the genetic code after the divergence of these two enzymes.

In another line of research, we aim to elucidate the evolutionary pathway of the genetic code prior to LUCA by reconstructing ancestral sequences of Class IIa ARSs based on composite phylogenetic trees (Furukawa et al. 2022). Through comparative analyses of these ancestral sequences, we explored the evolution of amino acid specificity among Class IIa ARSs (Furukawa et al. 2022) and experimentally reconstructed and characterized several ancestral ARSs from Classes I and II (S. Yokobori et al., in preparation).

Like ARSs and other translation-related proteins, tRNAs have also undergone evolutionary changes. To investigate the substrate specificities of ancestral translation factors and ARSs, it is necessary to examine tRNAs that are contemporaneous with these ancestral proteins. Only through such complementary analyses can ancient translation systems be experimentally validated. Therefore, we argue that, like elongation factors and ARSs, tRNAs should also be investigated using ASR-based approaches. However, as noted above, most prior studies of ancestral tRNAs remain theoretical (e.g. Tamura 2015; Root-Bernstein et al. 2016), and empirical ASR-based research on tRNAs is still quite limited (de Farias 2013).

As a first step toward reconstructing an early translation system, we inferred the ancestral tRNA sequences corresponding to LUCA, LACA, and LBCA for all 20 standard amino acids and characterized their features. Our ultimate goal is to reconstruct a “primitive translation system” composed entirely of tRNAs from LUCA or earlier lineages, providing a foundation to explore the nature of early translation. Two key challenges are (1) determining the minimal set of anticodons and (2) acquiring functional tRNAs for all amino acids.

To evaluate the functionality and anticodon requirements, we focused on LUCA tRNA^Ala^ and tRNA^Ser^. Since their anticodons are not recognized by their respective ARSs (Hou and Schimmel 1988; Francklyn and Schimmel 1989), their anticodons can be modified without affecting aminoacylation specificity. We engineered LUCA and *E. coli* tRNA^Ala^ and tRNA^Ser^ genes with anticodons (UGU, CGU, AGU, and GGU) to decode Thr codons. We also synthesized Thr codon-free reporter genes beginning with ACA, ACC, ACG, or ACT codons for T7 in vitro transcription (“ACN codon test genes”).

Using these tRNAs and ACN reporter constructs in a Thr-free version of the *E. coli* cell-free protein synthesis system (Shimizu et al. 2001), we assessed the functionality of the modified tRNAs and examined their codon–anticodon pairing properties.

## Materials and Methods

### Data Collection

Genome sequences from selected species representing Bacteria, Archaea, and Eukarya were retrieved from GenBank (Supplementary Table 1). Species were selected to maximize phylogenetic diversity across the three domains of life while minimizing redundancy from closely related taxa. tRNA genes were extracted from these sequences and classified according to their amino acid specificity, as inferred from their anticodon sequences. Twenty distinct tRNA groups were defined for the initial analysis.

### Phylogenetic Analyses and Ancestral Sequence Inference

After excluding tRNA sequences with potentially incorrect annotations and those that may have been recruited from other tRNA groups, each set was aligned using MAFFT version 7.017 (Katoh and Standley 2013). The initial alignments were refined manually based on the standardized tRNA numbering system (Giegé and Eriani 2023). Positions containing gaps were retained in the alignment unless they were ambiguously aligned, in which case they were excluded during the manual curation. Poorly aligned sequences were removed from the datasets. The resulting alignments of tRNAs, grouped by amino acid specificity, were then used to reconstruct phylogenetic trees.

Phylogenetic analysis of each tRNA species was performed using IQ-TREE version 1.6.12 (Nguyen et al. 2014). For each tRNA dataset, the best-fit substitution model was selected based on the Bayesian Information Criterion (BIC) implemented in IQ-TREE (see the legend of Supplementary Fig. S1). Branch support was assessed using nonparametric bootstrap analysis with 1,000 replicates.

tRNAs with the CAU anticodon include initiator tRNA (tRNA_i_^fMet^ or tRNA_i_^Met^), elongator tRNA_e_^Met^, and tRNA^Ile^_CAU_. However, many genome annotations do not distinguish among these types and often label them all as tRNA^Met^. Therefore, for tRNA^Met^, we first constructed a phylogenetic tree by aligning all genes with a CAU anticodon annotated as tRNA^Met^. Based on the resulting tree, we inferred the respective clades corresponding to the initiator and elongator tRNAs, as these two types have distinct conserved sequence features. Experimentally validated sequences of *E. coli* initiator and elongator tRNAs were used as references to identify these clades. For tRNA^Ile^, genes annotated as tRNA^Ile^_CAU_, tRNA^Ile^_GAU_, tRNA^Ile^_UAU_, and tRNA^Ile^_AAU_ were aligned, followed by phylogenetic analysis.

Ancestral sequence estimation was performed using MEGA version 10.1.7 (Kumar et al. 2018), with the tree topologies inferred by IQ-TREE specified as user-defined trees. Ancestral sequences of LACA and LBCA were inferred under the GTR+G4 model (Nei and Kumar 2000; Kumar et al. 2018; Stecher et al. 2020). Given the limited length and information content of tRNA sequences, the GTR+G4 model was adopted as a practical approximation for ancestral sequence reconstruction, as more complex models are constrained by the weak phylogenetic signal in short tRNA alignments. In MEGA, gaps were treated as character states rather than ignored during ancestral sequence estimation. After excluding the terminal CCA sequence and the − 1 nucleotide of tRNA^His^, the aligned tRNA sequences were used for phylogenetic analysis and ancestral sequence reconstruction of LACA and LBCA for each tRNA species.

Ancestral sequences were inferred using a maximum likelihood approach. Because the phylogenetic trees are unrooted, we placed LACA and LBCA at opposite ends of the branch linking the archaeal cluster (containing mainly archaeal but some eukaryal sequences) and the bacterial cluster (containing mainly bacterial but with some eukaryal sequences). During ancestral sequence reconstruction in MEGA, the maximum probability of the inferred nucleotide at each site was recorded for each reconstructed ancestral tRNA sequence, in order to assess site-wise uncertainty in the ASR results.

Next, the ancestral sequences of LACA and LBCA for each tRNA were aligned, and phylogenetic inference and ancestral sequence reconstruction were performed as described above. The position of LUCA was inferred as the node joining the LACA and LBCA sequences for each tRNA species.

### In Vitro Transcription of LUCA and *E. coli* tRNA Genes

**Fig. 1:**
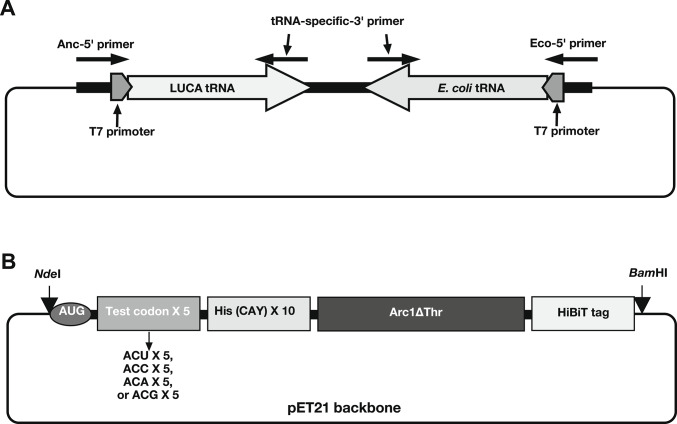
Outline of construction of plasmids carrying LUCA and *E. coli* tRNA genes and codon test genes. **A** LUCA and *E. coli* tRNA genes. Pairs of tRNA genes from *E. coli* and LUCA with identical anticodon sequences were synthesized. Primers (Supplementary Table S2) were used to amplify the transcription templates for in vitro synthesis. **B** Codon test genes. The constructs were based on the pET23a vector. Test genes were inserted between the *Nde*I and *Bam*HI sites, with the start codons included in the *Nde*I site. Two versions were constructed: one containing a HiBiT tag and the other without

tRNA^Ala^ and tRNA^Ser^ genes from both LUCA and *E. coli* were selected for anticodon sequence modifications. tRNA^Ala^ and tRNA^Ser^ were selected because their anticodons are not primary identity elements for aminoacylation, allowing anticodon modification without disrupting amino acid specificity. Genes in which the anticodons were modified to AGT, CGT, GGT, or TGT were synthesized and inserted into the pTwist Amp High Copy vector (Twist Bioscience, South San Francisco, CA, USA). As shown in Fig. 1A, pairs of tRNA genes from LUCA and *E. coli*, corresponding to the same amino acid and sharing the same modified anticodon, were each placed under a T7 promoter and inserted into the vector. These constructs were used to amplify the respective tRNA genes. For tRNA^Ser^ with anticodons AGT, CGT, and TGT, new gene constructs were synthesized and inserted into the pUC-GW-Amp vector (GENEWIZ, Azenta Life Sciences, South Plainfield, NJ, USA). The resulting plasmids were designated as: pTwist-Ala-AGU, pTwist-Ala-CGU, pTwist-Ala-GGU, pTwist-Ala-UGU, pUC-GW-Ser-AGU, pUC-GW-Ser-CGU, pUC-GW-Ser-GGU, and pUC-GW-Ser-UGU.

### Monitor Genes of Codon Recognition

The expression vector for *Arc1ΔThr* gene (Shibue et al. 2018) was kindly provided by Dr. Satoshi Akanuma at Waseda University. The *Arc1ΔThr* gene, which lacks codons for threonine (Thr) and cysteine (Cys), was inserted between the *Nde*I and *Bam*HI sites of the pET23a vector, resulting in the plasmid pET23a-*Arc1ΔThr*. The *Arc1ΔThr* gene does not contain any tag sequences.

Five threonine “monitor” codons (ACA, ACC, ACG, or ACT) followed by ten histidine codons were added using the following procedure to the 5′ end of the *Arc1ΔThr* gene. First, inverse PCR was done on pET23a-*Arc1ΔThr* using primers Arc1ΔThr_inverse_first_to_5end and Arc1ΔThr_inverse_first_to_3end (see Supplementary Table S2). In this step, five Thr codons (ACA–ACC–ACG–ACT–ACG) were added to the 5′ end of the gene, along with three codons (GGT–TCT–TCT, encoding Gly–Ser–Ser) immediately downstream of the initiation codon. The resulting PCR product was self-ligated (circularized) and used for transformation of *E. coli* JM109 competent cells (ECOSTM Competent *E. coli* JM109; NIPPON GENE, Tokyo, Japan).

Plasmids were purified using the QIAquick Miniprep Kit (QIAGEN, Hilden, Germany), following the manufacturer’s instructions. The purified plasmid was then used as a template for a second inverse PCR using primers Arc1ΔThr_inverse_second_to_5end and Arc1ΔThr_inverse_second_to_3end, in order to insert ten His codons between the five Thr codons and the *Arc1ΔThr* gene. The resulting PCR product was again self-ligated and cloned.

Subsequently, the 5′ Thr codons were replaced with five copies of a single type of codon (either ACA, ACC, ACG, or ACT) via a third round of inverse PCR. This was done using primer Arc1ΔT_inverse_third_to_5end and one of the following reverse primers: Arc1ΔT_ACA5_inverse_third_to_3end, Arc1ΔT_ACC5_inverse_third_to_3end, Arc1ΔT_ACG5_inverse_third_to_3end, or Arc1ΔT_ACU5_inverse_third_to_3end. Each PCR product was again self-ligated and cloned.

All inverse PCR and self-ligation steps described above were completed using the KOD Plus Mutagenesis Kit (Toyobo Co., Ltd., Osaka, Japan). Oligonucleotides were synthesized by means of Hokkaido System Science Co., Ltd. (Sapporo, Japan), Eurofins Scientific SE (Luxembourg City, Luxembourg), or Integrated DNA Technologies, Inc. (Coralville, IA, USA). Self-ligated PCR products were used for the transformation of *E. coli* JM109 competent cells. Overnight cultures from selected colonies were used for plasmid purification using the QIAquick Miniprep Kit (QIAGEN).

The resulting plasmids were named: pET23a-ACA5-Arc1ΔThr, pET23a-ACC5-Arc1ΔThr, pET23a-ACG5-Arc1ΔThr, and pET23a-ACU5-Arc1ΔThr.

Furthermore, the HiBiT tag sequence was added to the 3′ end of each modified gene (as previously done for the test codons and His codons at the 5′ end) using a similar inverse PCR strategy. The resulting plasmids were designated: pET23a-ACA5-Arc1ΔThr-HiBiT, pET23a-ACC5-Arc1ΔThr-HiBiT, pET23a-ACG5-Arc1ΔThr-HiBiT, and pET23a-ACU5-Arc1ΔThr-HiBiT (see Fig. 1B).

### In Vitro Transcription of tRNA

Each vector carrying a tRNA gene was used to transform *E. coli* JM109 chemically competent cells (ECOSTM Competent *E. coli* JM109). Plasmids were then purified using the QIAquick Miniprep Kit. Each tRNA gene, along with its upstream T7 promoter, was amplified by PCR using primers listed in Supplementary Table S2.

PCR products were purified by agarose gel electrophoresis followed by extraction using the QIAquick Gel Purification Kit (QIAGEN, Hilden, Germany). In vitro transcription of each purified tRNA gene was done using the ScriptMAX^®^ Thermo T7 Transcription Kit (Toyobo Co., Ltd., Osaka, Japan). The resulting tRNA transcripts were purified with the NucleoSpin^®^ miRNA Kit (MACHEREY-NAGEL GmbH & Co. KG, Düren, Germany), according to the manufacturer’s protocol.

Purified transcribed tRNAs were analyzed by polyacrylamide gel electrophoresis (PAGE) with and without 7 M urea to assess the accuracy of transcription termination and to examine possible tertiary structural variations in the transcripts. The concentration of each tRNA was measured using a Qubit 3.0 Fluorometer (Thermo Fisher Scientific Inc., Waltham, MA, USA) with the Qubit RNA BR Assay Kit (Thermo Fisher Scientific).

### In Vitro Translation and Analysis

In vitro translation was done using a customized PURE*frex* 1.0 system (GeneFrontier Corporation, Kashiwa, Chiba, Japan). In this customized version, threonine (Thr) was omitted from Solution I of the kit. Each reaction mixture (total volume: 20 µL) was prepared in a 200 µL PCR tube and contained a tRNA transcript with a test anticodon, a plasmid vector encoding a monitor gene containing the test codon, and the customized PURE*frex* solutions lacking Thr. The mixture was incubated at 37 °C for 4 h. As controls, a reaction containing the ACU-test codon monitor plasmid and supplemented with Thr was included, using the same customized PURE*frex* solution lacking endogenous Thr, and a reaction containing only customized PURE*frex* solution lacking endogenous Thr.

The results of the in vitro translation assays were analyzed by SDS–polyacrylamide gel electrophoresis (SDS–PAGE), followed by Coomassie Brilliant Blue (CBB) staining. The gel consisted of a 5% stacking layer and a 15% separating layer, using a Tris–Glycine buffer system.

### Protein Quantification with HiBiT System

Luminescence (LUM) was measured using the Nano-Glo^®^ HiBiT Detection System (Promega Corporation, Madison, WI, USA). In each well of a white, opaque 96-well ProxiPlate (shallow well; Revvity, Inc., Waltham, MA, USA), 25 µL of stepwise-diluted cell-free protein synthesis reaction solution or HiBiT control protein was dispensed. Next, 25 µL of Nano-Glo^®^ HiBiT Lytic Reagent was added to each well. After mixing, the plate was incubated for 10 min to allow equilibration between LgBiT and HiBiT. Luminescence intensity was then measured using a VICTOR Nivo™ multimode plate reader (Revvity).

To address the possibility that luminescence values might exceed the detection range, each sample was subjected to serial dilutions. A buffer containing 0.05 M Tris-HCl (pH 6.5) and 0.05 M NaCl was used for dilution. For each sample, three plates were prepared, with three technical replicates per plate. The average luminescence from each plate was calculated, and these averages constituted one data set. A total of four such independent sets were obtained. Among the dilution series, values from the 500-fold dilution—falling within the linear detection range—were used for the final analysis.

## Results and Discussion

In this study, we addressed three key questions regarding the early evolution of tRNA and the translation system: whether the evolutionary history of tRNA reflects that of ARSs; whether the inferred ancestral tRNA retains the identity elements necessary for aminoacylation; and whether such ancestral tRNA is functionally compatible with the modern translation system such as *E. coli*. Our analyses focus on these functional and evolutionary features rather than on detailed phylogenetic relationships among tRNAs.

### Individual tRNA Phylogenies and Ancestral tRNA Sequences for LACA and LBCA

The phylogenetic analyses presented here are primarily intended to provide a framework for ancestral sequence reconstruction, rather than to resolve detailed evolutionary relationships among different tRNA species. Given the short length of tRNA sequences and their limited phylogenetic signal, the inferred topologies are interpreted cautiously and are used primarily for inferring ancestral sequences of LACA and LBCA.

**Fig. 2:**
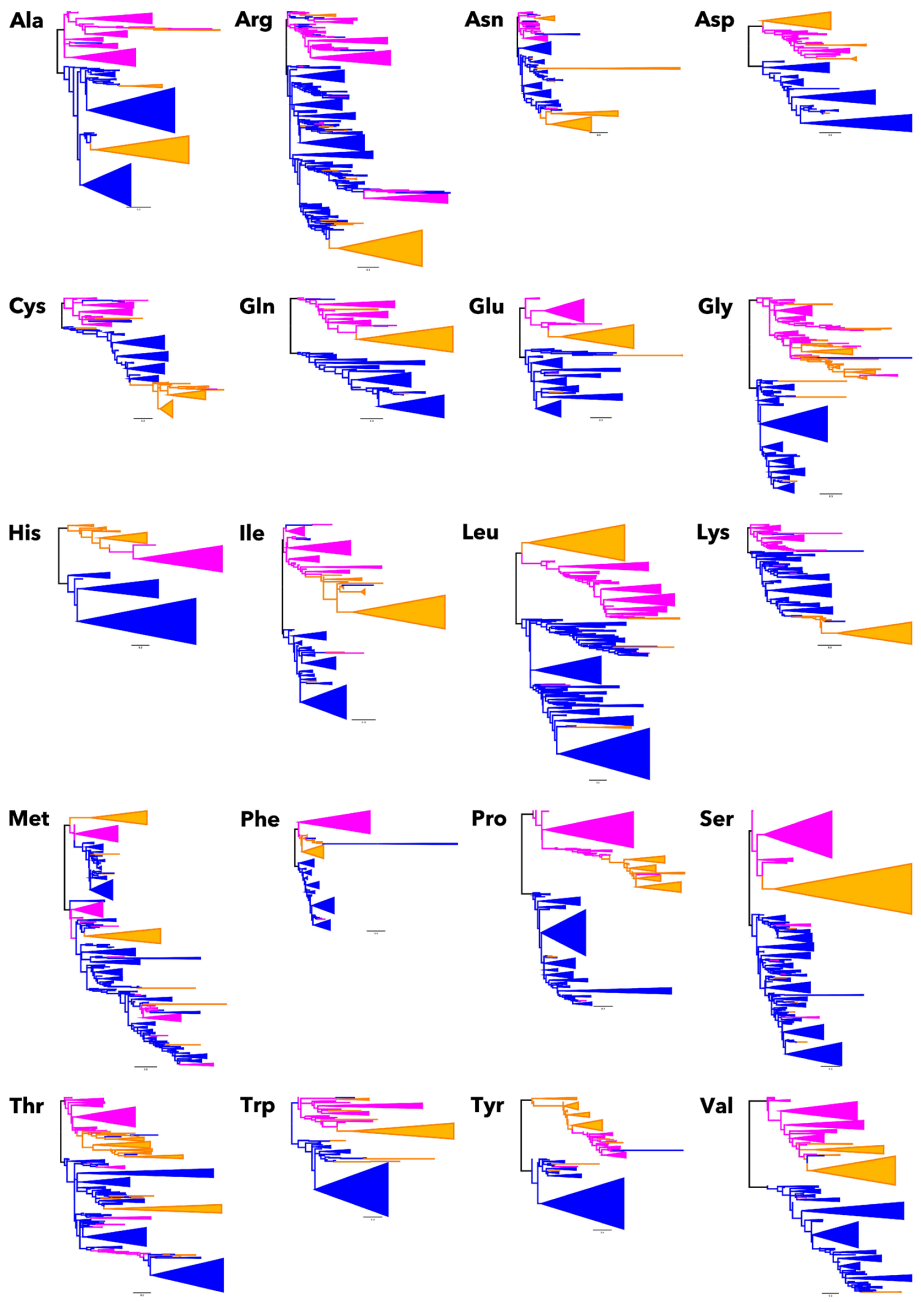
Summary of the phylogenetic trees for individual tRNA species. Each tree is labeled with the three-letter code of the corresponding amino acid. Archaea, Bacteria, and Eukarya are indicated by magenta, blue, and orange, respectively. Trees are unrooted, but the branch between the archaeal and bacterial clades likely includes a root. The full trees are presented in Supplementary Figure S1

Figure 2 provides an overview of the phylogenetic trees for each tRNA (for details, see Supplementary Fig. S1), under the assumption that LUCA is positioned on the branch connecting the archaeal and bacterial clusters (see above for details). Bootstrap support values are provided in Supplementary Fig. S1. As expected given the short length of the tRNA alignment, the support for some deep internal nodes is limited. However, broader clustering patterns—particularly the separation between archaeal and bacterial lineages into distinct clusters—were generally recovered, with bootstrap support values often exceeding 80 in most tRNA datasets (except for tRNA^Glu^ and tRNA^Gln^). Accordingly, interpretations based on weakly supported nodes are treated with caution.

In this framework, several eukaryal tRNAs—namely tRNA^Ala^, tRNA^Arg^, tRNA^Asn^, tRNA^Cys^, and tRNA^Lys^—are placed within the bacterial clades in the inferred trees. In contrast, tRNA^Gln^, tRNA^Glu^, tRNA^Gly^, tRNA^Ile^, tRNA^Phe^, tRNA^Pro^, tRNA^Ser^, tRNA^Thr^, tRNA^Trp^, and tRNA^Val^ tend to cluster within the archaeal clades. However, in the cases of tRNA^Asp^, tRNA^His^, and tRNA^Tyr^, archaeal sequences are positioned within eukaryal clades. In addition, eukaryal tRNA^Leu^ forms a distinct third group.

Previous studies (e.g., Furukawa et al. 2017) have proposed that the eukaryal cytoplasmic ARSs are mosaic. Several ARSs (SerRS, GlyRS (α₂ type), GluRS, LeuRS, TrpRS, PheRS, CysRS, TyrRS, ProRS, and HisRS) are thought to have archaeal origins, whereas others (ThrRS, ValRS, IleRS, AspRS, ArgRS, and MetRS) are inferred to derive from bacterial lineages, in some cases involving horizontal gene transfer from archaea (IleRS, AspRS, and ArgRS—as well as one subgroup of eukaryal MetRS). In addition, eukaryal AsnRS and AlaRS include multiple evolutionary lineages of distinct origins (i.e., bacterial and archaeal). For LysRS, Class II LysRS is found in eukaryotes, most bacteria, and some archaea, whereas Class I LysRS is found in most archaea and a few bacteria. The bacterial affinity observed for eukaryal tRNA^Lys^ in our analyses may therefore reflect co-evolution with Class II LysRS. Similarly, the archaeal affinities observed for several eukaryal tRNAs (tRNA^Glu^, tRNA^Gly^, tRNA^Phe^, tRNA^Pro^, tRNA^Ser^, and tRNA^Trp^)—and the bacterial affinity of eukaryal tRNA^Arg^—are broadly consistent with the hypothesis that tRNAs co-evolved with their corresponding ARSs.

However, discrepancies between tRNA and ARS phylogenetic patterns are also evident. For several tRNA species, the inferred placements do not match the expected origins based on ARS evolution, suggesting that the evolutionary histories of tRNAs and their cognate synthetases are not fully coupled.

While some topological relationships remain uncertain due to limited phylogenetic signal, these trees provide a consistent and practical framework for ancestral sequence inference and for exploring the early evolution of the translation system. Using this framework, ancestral tRNA sequences corresponding to LACA and LBCA were inferred for each tRNA species. To evaluate site-wise uncertainty in these reconstructions, we recorded the maximum probability of the inferred nucleotide at each site. These site-wise maximum probabilities for LACA and LBCA tRNAs are summarized in Supplementary Fig. S2.

### Composite tRNA Tree and Ancestral tRNA Sequences of LUCA

**Fig. 3:**
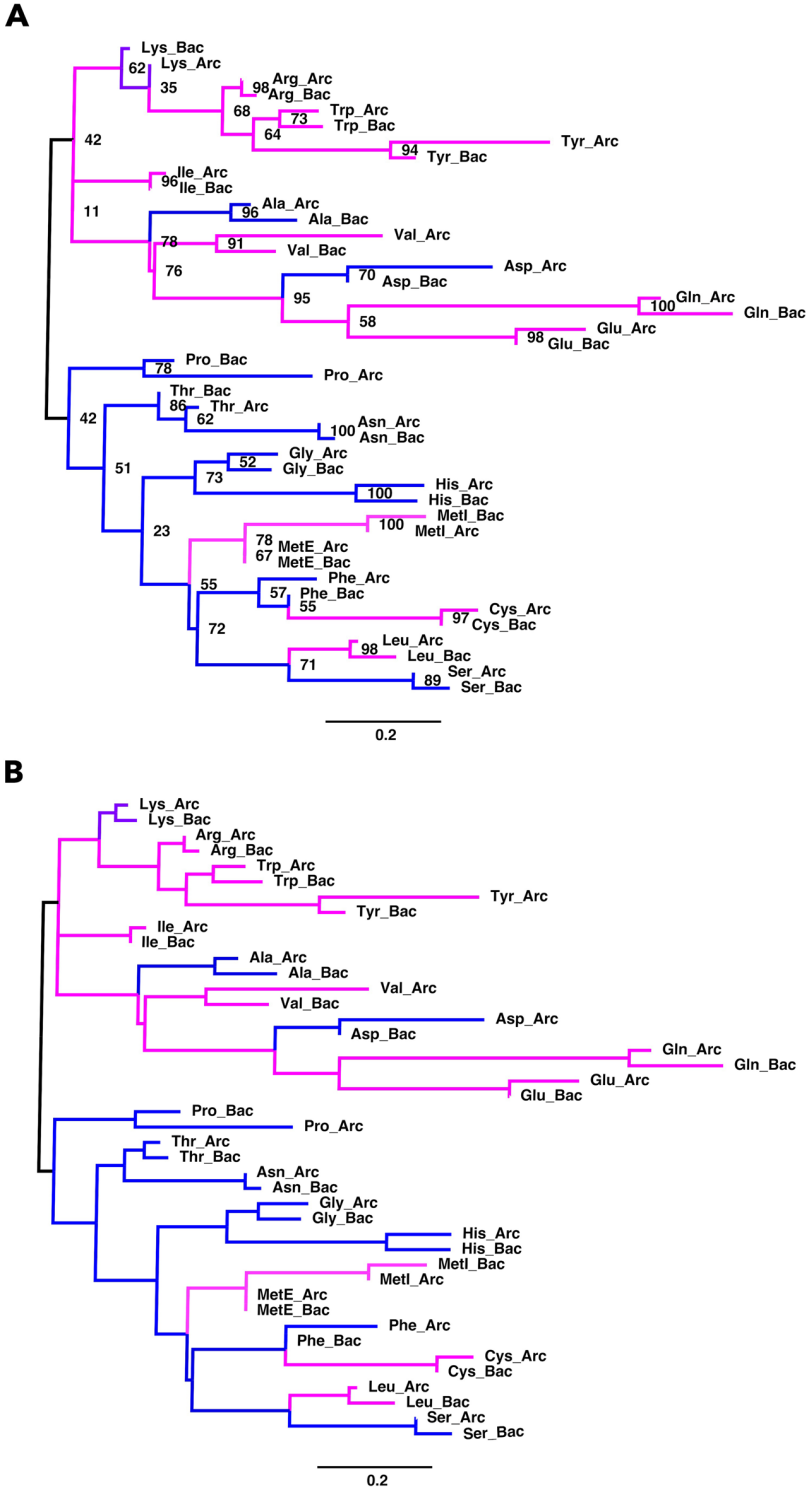
Phylogenetic tree of ancestral (LACA and LBCA) tRNAs. **A** Tree without topological constraints. The numbers at the nodes indicate the bootstrap values. **B** Tree with topological constraint (see text) used to infer the LUCA tRNA sequence for each species

To integrate the ancestral sequences inferred for individual tRNA species and to define the position of LUCA, we constructed a composite phylogenetic tree based on the ancestral tRNAs of LACA and LBCA. We inferred 21 pairs of ancestral tRNA sequences corresponding to the 20 standard amino acids, including both initiator and elongator tRNAs for methionine, from LACA and LBCA. Using these 42 ancestral tRNA sequences, we reconstructed a composite phylogenetic tree of ancestral tRNAs from LACA and LBCA (Fig. 3), following the approach of de Farias (2013). Phylogenetic inference was done using IQ-TREE with the K3Pu + F+I+G4 substitution model (Nguyen et al. 2014). The nodes connecting LACA and LBCA for each tRNA were interpreted as representing the positions of the LUCA.

**Fig. 4:**
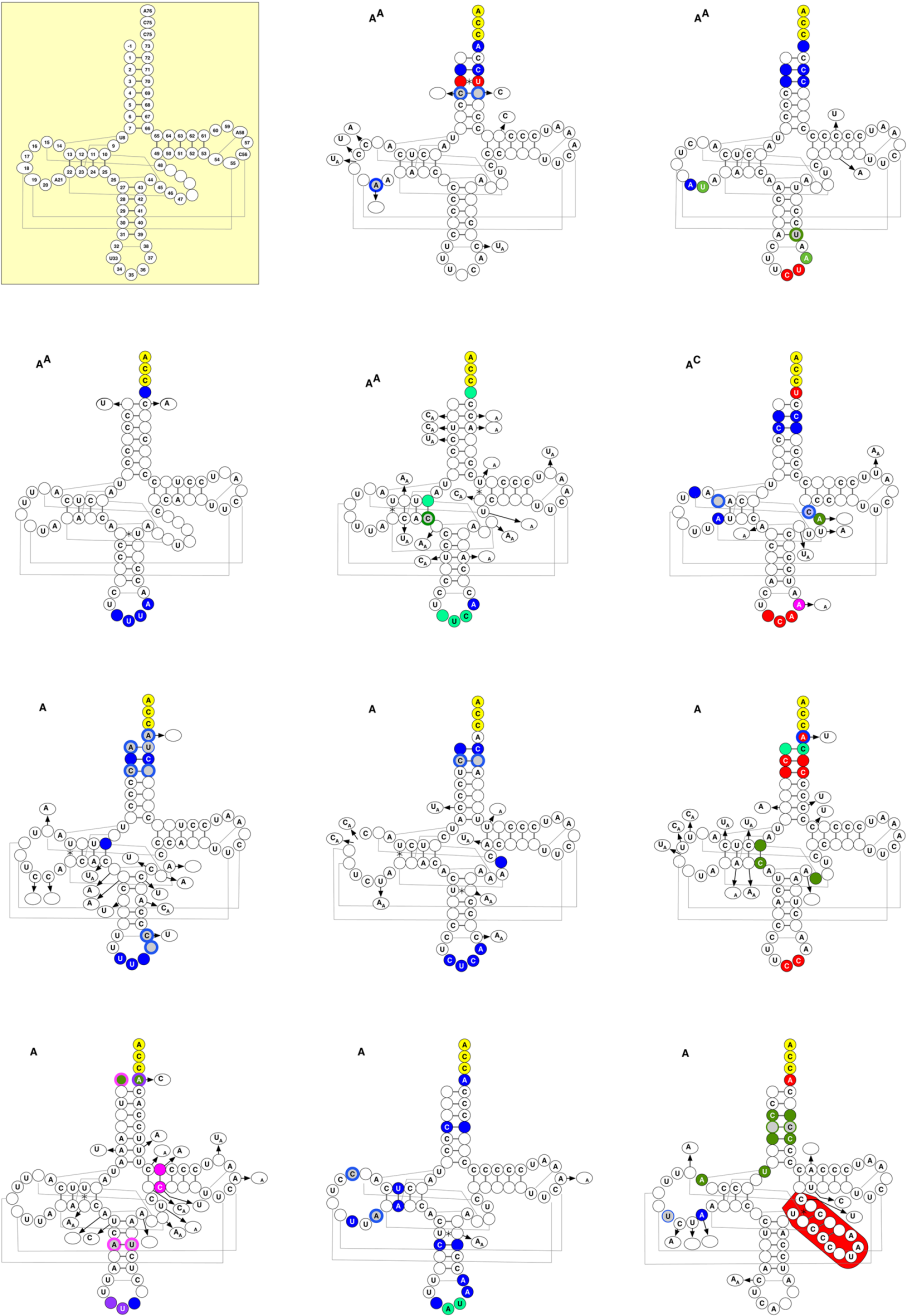

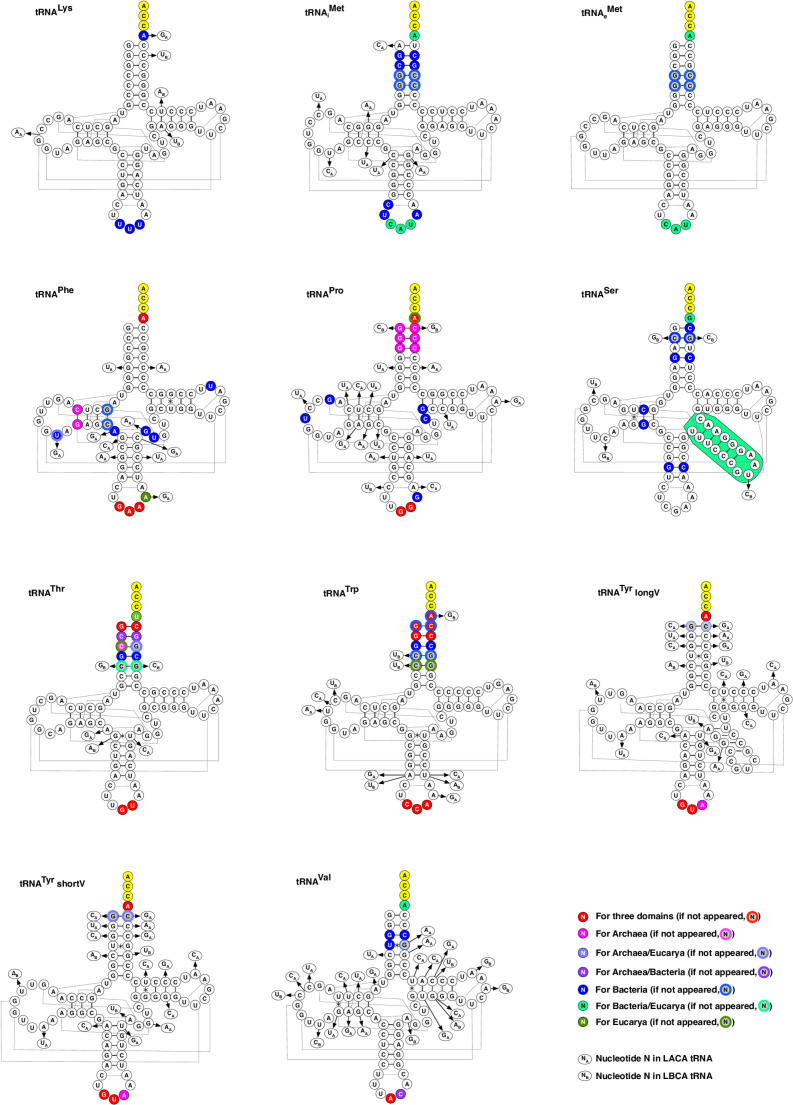
Predicted cloverleaf secondary structures of LUCA tRNAs. Nucleotide numbering follows the system proposed by Giegé and Eriani (2023). The nucleotides of LACA and LBCA tRNAs that differ from those of LUCA tRNAs are also shown (N_A_ for LACA tRNAs and N_B_ for LBCA tRNAs)

However, tRNA^Lys^, tRNA^Phe^, and tRNA^Thr^ appeared as paraphyletic groups in the ML tree (Fig. 3A). To address this, a constraint tree was generated (Fig. 3B), enforcing the monophyly of each tRNA species while preserving the inter-species relationships as closely as possible to those in the ML tree. Both the ML and constraint trees were inferred under the same model. The log-likelihoods of ML and constraint trees were − 1797.5 and − 1802.0, respectively—a difference of only 4.5. The constraint tree is not rejected by Kishino-Hasegawa test (Kishino and Hasegawa 1989) (unweighted and weighted), Shimodaira-Hasegawa test (Shimodaira and Hasegawa 1999) (unweighted and weighted), or the approximately unbiased test (Shimodaira and Goldman 2002). Therefore, we used the constraint tree (Fig. 3B) for subsequent analyses, including estimation of the LUCA sequence for each tRNA species. These inferred sequences are presented in Fig. 4. To evaluate site-wise uncertainty in the LUCA reconstructions, we recorded the maximum probability of the inferred nucleotide at each site. These site-wise maximum probabilities for LUCA tRNAs are summarized in Supplementary Fig. S3.

This composite tree should be regarded as an operational framework for ancestral sequence inference rather than a definitive representation of evolutionary relationships among tRNAs. Therefore, the following observations should be interpreted with caution.

The topology of the tRNA composite tree (Fig. 3A and B), which served as the basis for ancestral sequence inference, does not match that of the ARS tree. In the tRNA composite tree, tRNAs corresponding to Class I and Class II ARSs do not form distinct clades. tRNA^Ala^ and tRNA^Asp^ appear in the upper half of the trees (Fig. 3A and B), which roughly corresponds to tRNAs for Class I ARSs, while tRNA^Cys^, tRNA^Leu^, and tRNA^Met^s are found in the lower half, corresponding to Class II ARSs.

For subclass Ia ARSs, the relationship (MetRS, (LeuRS, (IleRS, ValRS)) is widely accepted (Nagel and Doolittle 1995). However, in our tRNA tree, tRNA^Met^, tRNA^Leu^, tRNA^Ile^, and tRNA^Val^ are located in separate branches (Fig. 3A and B). Similarly, tRNA^Arg^ and tRNA^Cys^ are apart from other tRNAs for Class Ia ARSs. Notably, tRNA^Leu^ clusters with tRNA^Ser^, whose synthetase (SerRS) belongs to subclass IIa. Both tRNAs have long variable arms, which may reflect a shared evolutionary origin. In *Candida albicans*, tRNA^Ser^_CAG_ is known to be leucylated (Suzuki et al. 1997; Zhou et al. 2013). However, this observed sister relationship could be an artifact, potentially due to the inclusion of gap-containing sites in the alignment or convergent features associated with their long variable arms.

In contrast, tRNA^Trp^ and tRNA^Tyr^—both substrates of subclass Ic ARSs, form a group, despite bacterial tRNA^Tyr^ having a long variable arm. If our phylogenetic inference is accurate, the long variable arm of bacterial tRNA^Tyr^ may have emerged after divergence from the archaeal/eukaryal lineage.

tRNA^Glu^ and tRNA^Gln^, which correspond to subclass Ib ARSs, also form a group. Their sister group is tRNA^Asp^, a substrate of a subclass IIb ARS. However, tRNA^Asn^ appears in a separate position in the tree. Interestingly, *Helicobacter pylori* AspRS has been reported to possess glutamylation activity toward tRNA^Glu^ in addition to its canonical role in aspartylation of tRNA^Asp^ and tRNA^Asn^ (Rathnayake and Hendrickson 2019), which may be related to the proximity of tRNA^Asp^ and tRNA^Glu^ in our phylogenetic tree.

All tRNAs corresponding to Class IIa ARSs—namely GlyRS (α₂), HisRS, ThrRS, ProRS, and SerRS, are in “Class II clade” in the tRNA composite tree, along with tRNA^Asn^ and some tRNAs associated with Class I ARSs (e.g., tRNA^Me^ts, tRNA^Cys^, and tRNA^Ser^) (Fig. 3A and B). Notably, tRNA^His^ and tRNA^Gly^ form a group. HisRS and GlyRS (α_2_) are members of subclass IIa ARS. Although GlyRS is believed to have diverged early within subclass IIa, GlyRS (α₂) has been shown to group with HisRS under certain conditions in molecular trees (Furukawa et al. 2022), consistent with the topology of our tree, although this pattern may also be influenced by methodological factors.

It should be noted that the ASR approach used here relies on a homogeneous substitution model (GTR+G4), which may not fully capture site-specific evolutionary constraints. More sophisticated models, such as site-heterogeneous models, may improve reconstruction accuracy and should be explored in future studies. Additionally, the treatment of gaps in the alignment may influence inferred ancestral sequences, representing another source of uncertainty. These methodological limitations, together with the relatively short length of tRNA sequences, suggest that some inferred relationships and ancestral states should be interpreted with caution. The observed incongruence between tRNA and ARS phylogenies may reflect genuine biological differences, but could also be influenced by methodological limitations inherent to short tRNA sequences and model assumptions.

### tRNA Identity Elements in Ancestral tRNAs and Their Evolution

We next examined whether ancestral tRNAs retain identity elements required for aminoacylation, as this provides insight into the functional continuity of the translation system from the LUCA to extant organisms. In evaluating these features, we took into account the site-wise maximum probabilities obtained during ancestral sequence reconstruction. Most reconstructed sites showed maximum probabilities greater than 0.9, and most of the conserved residues and identity elements discussed below fell within these high-probability sites. Thus, the main conclusions concerning conserved sequence features are based largely on sites with relatively high reconstruction confidence.

Identity elements of tRNA are sequences and structural features recognized by their cognate ARSs. In Fig. 4, we show that LUCA, LACA, and LBCA tRNAs seem to retain identity elements mostly studied for *E. coli* tRNAs and less so for *Thermus thermophilus*, *Saccharomyces cerevisiae*, *Aeropyrum pernix*, and other species’ tRNAs (see Giegé and Eriani 2023). In most cases, these identity elements are well conserved in ancestral tRNAs. In addition, in Fig. 4, the LUCA, LACA, and LBCA tRNAs are compared with some bacterial, archaeal, and eukaryal tRNAs including *E. coli*,* T. thermophilus*, and *S. cerevisiae*.

tRNA^Ala^: The major identity determinant of tRNA^Ala^ is the G_3_-U_70_ wobble pair, rather than the anticodon, which governs the alanylation of *E. coli* tRNA^Ala^ (Hou and Schimmel 1988; McClain et al. 1988; Park et al. 1989). This wobble pair, along with G_2_-C_71_ and A_73_, is well conserved across LACA, LBCA, and LUCA tRNA^Ala^ (Fig. 4).

tRNA^Arg^: The major identity determinants in *E. coli* tRNA^Arg^ include A_20_, C_35_ and U_36_/G_36_ and the discriminator base A_73_/G_73_ (McClain and Foss 1988; Schulman and Pelka 1989; Tamura et al. 1992) A_20_ is the key determinant for arginylation by *E. coli* ArgRS. These elements are conserved in LUCA, LBCA and LACA tRNA^Arg^ (discriminator G_73_) (Fig. 4).

tRNA^Asn^. The major identity determinants of *E. coli* tRNA^Asn^ are the anticodon (G_34_, U_35_, and U_36_) and the discriminator base G_73_ (Li et al. 1993); LACA, LBCA and LUCA tRNA^Asn^ all retain G_34_, U_35_, U_36_, A_37_ and G_73_ (Fig. 4).

tRNA^Asp^: Conserved identity elements of tRNA^Asp^ across *S. cerevisiae*, *E. coli*, and *T. thermophilus* include _73_, G_34_, U_35_, C_36_, and C_38_ (Nameki et al. 1992; Giegé et al. 1996, 1998). All these are present in LACA, LBCA and LUCA tRNA^Asp^ (Fig. 4).

tRNA^Cys^: The discriminator U_73_ and the anticodon G_34_-C_35_-A_36_ are major identity determinants in *E. coli* (Pallanck et al. 1992; Komatsoulis and Abelson 1993), and all of these are conserved in LUCA, LBCA and LACA tRNA^Cys^ (Fig. 4). However, G_15_:G_48_ of an important tRNA element in *E. coli* tRNA^Cys^ (Hou et al. 1993; Hauenstein et al. 2004) is absent in LUCA, LBCA and LACA tRNA^Cys^.

tRNA^Gln^: Identity elements for tRNA^Gln^ in *E. coli* include G_73_, U_1_-A_72_, G_2_-C_71_, G_3_-C_70_, U_34_, U_35_, and G_36_ (Jahn et al. 1991). The base pair of 10th and 25th nucleotides and the U_35_ are also important binding sites for *E. coli* GlnRS, with G_36_ contributing to both binding and recognition (Ibba et al. 1996). LACA, LBCA and LUCA tRNA^Gln^ retain the G_2_-C_71_, G_10_, and anticodon sequences (U_34_/C_34_ U_35_ G_36_), respectively (Fig. 4). However, LACA and LUCA tRNA^Gln^ have A_73_ instead of G_73_. Ancestral trait of tRNA^Gln^ identity might be G_2_-C_71_, G_10_, anticodon sequences (U_34_/C_34_ U_35_ G_36_) and discriminator A_73_.

tRNA^Glu^: Strong identity determinants of tRNA^Glu^ are U_34_, U_35_, C_36_ and A_37_, along with U_11_-A_24_, U_13_-G_22_:A_46_ and the absence of 47th nucleotide serve as major identity determinants for tRNA^Glu^ (Sekine et al. 1996). LACA tRNA^Glu^ retains U_34_, while LBCA and LUCA tRNA^Glu^ substitute it with C_34_ (Fig. 4). The presence of U_11_-A_24_ and U_13_-G_22_:A_46_ and the absence of 47th nucleotide is also common to the three ancestral tRNA^Glu^s. The ancestral trait identity of tRNA^Glu^ appears to be C_34_/U_34_, U_35_, C_36_ and A_37_, U_11_-A_24_, U_13_-G_22_:A_46_, and the absence of 47th nucleotide.

tRNA^Gly^: In bacterial tRNA^Gly^, the discriminator base U_73_ is a critical identity element; however, A_73_ is typical of eukaryal and archaeal tRNA^Gly^. Additionally, the sequences G_1_-C_72_ pair, C_35_, C_36_, and the (G_10_-Y_25_):G_45_ triplet are conserved (Mazauric et al. 1998). The tRNA^Gly^ of LBCA retains the bacterial U_73_, while those of LACA and LUCA possess A_73_. The mutation of U_73_ of *E. coli* tRNA^Gly^ reduces glycylation activity, indicating that U_73_ functions as an identity element for GlyRS (Shimizu et al. 1992). By contrast, in the archaeon *A. pernix*, the A_73_ discriminator is not essential for tRNA^Gly^ identity (Okamoto et al. 2005). Further mutational analyses in *E. coli*, *T. thermophilus*, and *S. cerevisiae* have identified a broader identity set that includes the first two base pairs of the acceptor stem (G_1_–C_72_ and C_2_–G_71_) as well as the anticodon bases C_35_ and C_36_ (Mazauric et al. 1999). In *T. thermophilus*, additional identity elements have been identified, including the C_50_–G_64_ base pair, G_10_, and U_16_ (Mazauric et al. 1999). tRNA^Gly^ in LACA, LBCA, and LUCA retain several conserved features: G_1_–C_72_, C_2_–G_71_, G_3_–C_70_, and the anticodon nucleotides C_35_ and C_36_. The identity elements C_50_–G_64_ and G_10_ are also conserved among these ancestral lineages.

tRNA^His^: In *E. coli* (Himeno et al. 1989)d *cerevisiae* tRNA^His^ (Nameki et al. 1995), G_− 1_ and anticodon (G_34_, U_35_, G_36_) have been shown to play an essential role in identity. In Bacteria, the discriminator C_73_ forms a base pair with G_− 1_, while the discriminator is A_73_ in Eukarya (cytoplasm). Both C_73_ and A_73_ are found in archaeal tRNA^His^ (Fig. 4). The G_− 1_-C_73_ pair is important for the recognition by the HisRS in *E. coli*. LACA and LUCA tRNA^His^ have A_73_, while LBCA tRNA^His^ has C_73_.

tRNA^Ile^: In tRNA^Ile^, key identity determinants include the discriminator base (A_73_) and anticodon nucleotides (G_34_/C_34_, A_35_, U_36_) followed by A_37_ (Muramatsu et al. 1988; Normanly et al. 1990; Pallanck and Schulman 1991). In *E. coli*, additional elements critical for isoleucylation and editing are found in the stem region (C_4_-G_69_, U_12_-G_23_, C_29_-G_41)_ and the D-loop (G_16_, D_20_, D_21_) (Nureki et al. 1994; Farrow et al. 1999; Hendrickson 2001). These are conserved in LUCA, LBCA and LACA tRNA^Ile^ except for G_16_.

tRNA^Leu^: In *E. coli* tRNA^Leu^, A_73_ is important for leucine identity, while the anticodon and long variable arm are less significant (Asahara et al. 1993). Mutagenesis studies have emphasized the importance of tertiary interactions, such as G_18_:U_55_, G_19_:C_56_ and U_54_:A_58_ in maintaining leucine identity (Du and Wang 2003). These are conserved in LUCA, LACA, and LBCA tRNA^Leu^.

tRNA^Lys^: The A_73_ of *E. coli* tRNA^Lys^ plays a critical role in lysine identity (McClain et al. 1990). Additionally, substitution experiments indicate that all three anticodon bases (U_34_, U_35_, U_36_) contribute to lysine identity (Tamura et al. 1992). LACA, LBCA and LUCA tRNA^Lys^ share U_34_, U_35_ and U_36_. However, unlike LBCA and LUCA tRNA^Lys^, LACA tRNA^Lys^ has G_73_ instead of A_73_. This suggests that A_73_ is an ancestral trait for tRNA^Lys^.

tRNA^Met^: The anticodon (C_34_, A_35_, U_36_) is a major identity element in tRNA^Met^ (Schulman and Pelka 1983, 1988). Additionally, the tRNA^fMet^-formyltransferase recognition elements (1–72 mismatch pairs and R_11_:Y_24_ base pairs) are known in tRNA_i_^fMet^ (Guillon et al. 1992b). Furthermore, G_29_-C_41_, G_30_-C_40_, and G_31_-G_39_ are important for *E. coli* tRNA_i_^fMet^ to play a role as initiator tRNA but not as elongator (Guillon et al. 1993). Among these, the 1–72 mismatch pair is not retained in LACA and LUCA tRNA_i_^Met^. This matches with the observation that in Archaea and Eukarya, the amino acid corresponding to the start codon is methionine (Met) rather than formylmethionine (fMet) (Ramesh and RajBhandary 2001; Schmitt et al. 2020).

tRNA^Phe^: The tRNA^Phe^ identity elements are the anticodon triplet G_34_, A_35_, and A_36_ and the discriminator A_73_ (Peterson and Uhlenbeck 1992). Additional elements critical for phenylalanylation include U_20_ and U_59_ (Peterson and Uhlenbeck 1992; Peterson et al. 1993). LACA, LBCA and LUCA tRNA^Phe^ retain G_34_, A_35_, A_36_, U_59_, and A_73_. However, U_20_ is conserved only in LBCA and LUCA tRNA^Phe^, and is absent in LACA tRNA^Phe^.

tRNA^Pro^: Identity elements of *E. coli* tRNA^Pro^ include G_35_, G_36_, A_37_, G_72_, A_73_, and the G_15_:C_48_ pair (Liu et al. 1995; Hasegawa and Yokogawa 2000). In archaeon *A. pernix* tRNA^Pro^, identity is also influenced by the G_1_–C_72_ base pair and anticodon (Yokozawa et al. 2003). All ancestral tRNAs retain G_15_:C_48_, G_35_, G_36_, and A_73_. However, A_37_ is absent from all ancestral sequences, suggesting a later acquisition. LBCA tRNA^Pro^ additionally retains C_1_-G_72_, while LACA and LUCA tRNA^Pro^ have G_1_-C_72_, suggesting that G_1_-C_72_ is the ancestral trait of tRNA^Pro^.

tRNA^Ser^: Serine identity is predominantly determined by the presence of a large variable arm rather than the anticodon (Normanly et al. 1992). In *E. coli*, conserved features include G_1_-C_72_ and G_2_-C_71_ across tRNA^Ser^ isoacceptors, (Jühling et al. 2009). Both *E. coli* and human SerRS recognize tRNA^Ser^ through its large variable arm and the discriminator G_73_ (Asahara et al. 1994; Breitschopf and Gross 1994, 1996; Breitschopf et al. 1995). All ancestral tRNAs exhibit large variable arms and retain G_1_-C_72_, G_4_-C_69_, C_11_-G_24_, G_30_-C_40_, and G_73_. LBCA retains G_2_-C_71_, while LACA and LUCA possess the reverse pair C_2_-G_71_.

tRNA^Thr^: Unlike many other tRNAs, the discriminator base is not essential for tRNA^Thr^ identity, though exceptions exist in species such as *Haloferax volcanii* and *S. cerevisiae* (Nameki 1995; Ishikura et al. 2000). Across five species (*E. coli*, *T. thermophilus*, *H. volcanii*, *A. pernix* and *S. cerevisiae*), the identity elements comprise acceptor stem base pairs (G_1_-C_72_, C_2_-G_71_, G_4_-C_69_) and anticodon nucleotides G_35_ and G_36_ (Hasegawa et al. 1992; Nameki 1995; Nameki et al. 1996; Ishikura et al. 2000; Iwaki et al. 2002). The primary identity elements consist of base pairs in the acceptor stem (G_1_-C_72_, C_2_-G_71_, G_4_-C_69_) and anticodon nucleotides (G_35_ and G_36_). All ancestral tRNA^Thr^ sequences examined retain these features.

tRNA^Trp^: In *E. coli* and *Bacillus subtilis*, identity elements for tRNA^Trp^ include the discriminator base G_73_, the anticodon (C_34_, C_35_, A_36_), and the A_1_-U_72_ base pair (Himeno et al. 1991; Xue et al. 1993). Additional acceptor stem base pairs (G_2_-C_71_, G_3_-C_70_, G_4_-C_69_) are also essential for *B. subtilis* tRNA^Trp^ (Xu et al. 2002). These base pairs are also present in *E. coli* tRNA^Trp^. In ancestral tRNA^Trp^s, G_2_-C_71_, G_3_-C_70_, and the three anticodon bases are conserved, but they have G_1_-C_72_ instead of A_1_-U_72_. In addition, LACA and LUCA tRNA^Trp^ have A_73_ instead of G_73_. The combination of A_73_ and G_1_-C_72_ in LACA and LUCA tRNA^Trp^ mirrors the identity pattern observed in *A. pernix* tRNA^Trp^ (Tsuchiya and Hasegawa 2009), suggesting that archaeal-type traits were retained in tRNA^Trp^.

tRNA^Tyr^: Bacterial tRNA^Tyr^ is a type II tRNA with a long extra arm, whereas archaeal and eukaryal tRNA^Tyr^ is a type I tRNA with a short extra arm. Our phylogeny suggests that LUCA tRNA^Tyr^ was a type I tRNA and that bacterial type II tRNA^Tyr^ emerged after the divergence of Bacteria from the archaeal/eukaryal lineage. Identity depends on the discriminator A_73_ and anticodon U_35_ (Bedouelle 1990), together with the first base pair (C_1_-G_72_ in archaea/eukaryotes; G_1_-C_72_ in bacteria) (Fechter et al. 2000; Iwaki et al. 2002; Bonnefond et al. 2005). LACA, LBCA and LUCA tRNA^Tyr^ share G_34_, U_35_ and A_73_. LACA tRNA^Tyr^ retains the archaeal-type C_1_-G_72_, while LBCA and LUCA tRNA^Tyr^ share the bacterial-type G_1_-C_72_.

tRNA^Val^ identity: The identity elements of tRNA^Val^ overlap with those of tRNA^Ile^ and tRNA^Met^, sharing A_73_ and A_35_. Additionally, C_36_ is a critical identity element, while G_20_ and G_45_ are minor recognition elements (Horowitz et al. 1999). In *E. coli*, mutations of base pairs G_3_-C_70_ or U_4_-A_69_ in the acceptor stem significantly affect activity (Tamura et al. 1991). Both LUCA and LBCA tRNA^Val^ lack U_4_-G_69_. LACA, LBCA, and LUCA tRNA^Val^ lacks G_20_, but G_20_ is also not conserved among *E. coli* tRNA^Val^ isoacceptors. Ancestral traits of tRNA^Val^ identity seem to be G_3_-C_70_, A_35_, C_36_, and A_73_.

Identity of tRNAs for transamidosome: The genes for AsnRS and GlnRS genes are absent in various bacterial and archaeal genomes (Curnow et al. 1996; Furukawa et al. 2017). In those organisms, Asn-tRNA^Asn^ and Gln-tRNA^Gln^ are synthesized via a two-step indirect process. First, non-discriminatory AspRS (ND-AspRS) and GluRS (ND-GluRS) aminoacylate the tRNA to form Asp-tRNA^Asn^ and Glu-tRNA^Gln^, respectively. Second, Asp-tRNA^Asn^ and Glu-tRNA^Gln^ are amidated in transamidosomes to Asn-tRNA^Asn^ and Gln-tRNA^Gln^, respectively (aaRS: tRNA: AdT, AdT is amidotransferase)(Bailly et al. 2007).

In the transamidosome, amidation of Asp-tRNA^Asn^ is promoted by the U_1_-A_72_, whereas the presence of G_1_-C_72_ pair and U_20a_ in the D-loop of tRNA^Asp^ inhibits this process (Bailly et al. 2006). The bacterial AdT (GatCAB) has a region that distinguishes between U_1_-A_72_ and G_1_-C_72_, while archaeal GatCAB lacks this specificity and can accept aminoacyl-tRNA with a G_1_-C_72_ base pair (Nakamura et al. 2010). In archaeal AdTs, the Asp-to-Asn conversion is promoted primarily by G_46_ and U_47_ in the variable region (Bailly et al. 2006); a similar recognition mechanism is thought to operate for Gln-tRNA^Gln^ formation.

AsnRS and GlnRS are believed to have evolved secondarily from AspRS and GluRS, respectively (Woese et al. 2000). AsnRS shares a common ancestor with archaeal AspRS, while GlnRS shares a common ancestor with eukaryal GluRS. Among AspRS, ND-AspRS is considered ancestral (Nair et al. 2016), and similarly, ND-GluRS is thought to be the ancestral form of GluRS (Siatecka et al. 1998). Therefore, the AspRS and GluRS enzymes of LACA, LBCA, and LUCA are likely to have been the ND type.

Regarding sequence features, LACA, LBCA and LUCA tRNA^Asn^ all retain G_46_ and U_47_ in the variable region. However, while LACA and LUCA tRNA^Asn^ have G_1_-C_72_, LBCA tRNA^Asn^ contains U_1_-A_72_, promoting amidation. Conversely, all three ancestral tRNA^Asp^ retain U_20a_, a feature that inhibits recognition by bacterial transamidosomes. These findings suggest that LACA and LUCA tRNA^Asn^ were not compatible with bacterial-type AdTs but instead were likely recognized by archaeal-type AdTs. In contrast, LBCA tRNA^Asn^ is likely to have been compatible with the bacterial-type transamidosome.

An important limitation of ancestral sequence reconstruction is that the maximum likelihood sequence represents only one of many possible ancestral states. Therefore, the inferred ancestral tRNAs analyzed here should be interpreted as representative sequences rather than exact historical reconstructions. Although most conserved residues and identity elements discussed above were inferred with high site-wise maximum probabilities, alternative ancestral states at some positions may still influence the interpretation. Future work could incorporate probabilistic sampling of ancestral sequences to be better account for this uncertainty and to evaluate the robustness of these inferences.

### Can Inferred tRNAs Function in *E. coli* Translation System?

To assess the functional relevance of the inferred sequences, we evaluated whether LUCA tRNAs are compatible with a modern translation system (*E. coli*). When the inferred LUCA tRNA sequences were compared with their cognate *E. coli* counterparts (Supplementary Fig. S4), sequence identities ranged from 50 to 85.1% (Supplementary Table S3). The lowest sequence identity was observed for tRNA^Glu^ with only 50.0–51.4% identity between LUCA and *E. coli* sequences.

As discussed above, most of the inferred LUCA tRNAs retain bacterial identity elements, particularly those characterized in *E. coli* (Fig. 4), suggesting that they may be partially compatible with the *E. coli* system. Specifically, the following LUCA tRNAs were found to include all known bacterial identity elements: tRNA^Ala^, tRNA^Asn^, tRNA^Asp^, tRNA^Lys^, tRNA_i_^Met^, tRNA_e_^Met^, and tRNA^Phe^. Other LUCA tRNAs did not retain the full set of bacterial identity elements, which is not unexpected given that identity elements are not necessarily conserved among bacteria, archaea, and eukaryotes.

However, several LUCA tRNAs may lack key residues required for efficient aminoacylation in *E. coli*. One such case is LUCA tRNA^Gly^, which has A_73_ instead of the canonical U_73_. Previous studies have shown that this substitution reduces glycylation activity approximately 11-fold in the *E. coli* system (Shimizu et al. 1992; Nameki et al. 1997). Another example is LUCA tRNA^His^, which carries A_73_ instead of C_73_, which is highly conserved in bacterial tRNA^His^. A study by Rosen et al. (2006) on the minihelix of tRNA^His^ suggested that the substitution of C_73_ by A_73_ in the minihelix does not affect the histidylation activity of *E. coli* HisRS, indicating that LUCA tRNA ^His^ may still function in the *E. coli* system.

A more critical exception is the LUCA tRNA_i_^Met^. In *E. coli*, the initiator tRNA_i_^fMet^ contains a conserved mismatch at the first base pair of the acceptor stem (C_1_-A_72_) – a signature feature essential for selective recognition by methionyl-tRNA formyltransferase (Lee et al. 1991). The absence of N-formylation of Met on tRNA^fMet^ was reported to reduce translation activity in the *E. coli* system ((Guillon et al. 1992a). It is also important for the recognition by translational initiation factor 2 (IF2) (Guenneugues et al. 2000). In contrast, LUCA tRNA_i_^Met^ has A_1_-U_72_ instead of the C_1_-A_72_ mismatch. This suggests that LUCA tRNA_i_^Met^ may not be a suitable substrate for *E. coli*’s formylation machinery and could be inefficient in supporting translation initiation.

Furthermore, previous studies have shown that tRNAs lacking post-transcriptional modifications often exhibit reduced translational activity, particularly for tRNAs corresponding to cysteine, glutamate, isoleucine, lysine, and phenylalanine in *E. coli*, and isoleucine and phenylalanine in *S. cerevisiae* (Giegé and Eriani 2023). Modifications at the wobble position (position 34) and adjacent to the anticodon (position 37) are known to play crucial roles in codon recognition, identity, and aminoacylation (e.g. Nureki et al. 1994; Senger et al. 1997; Madore et al. 1999; Liu et al. 2007; Giegé and Lapointe 2009; Wang et al. 2022). In particular, modifications at residue 34 of the wobble position or at position 37 adjacent to the anticodon can be important as identity elements.

In summary, while many LUCA tRNAs appear to retain sufficient identity elements to function in modern translation systems such as that of *E. coli*, it is crucial to experimentally assess the functionality of each individual LUCA tRNA species in such systems. Therefore, compatibility of LUCA tRNAs with the *E. coli* translation system should be regarded as a testable inference based on representative reconstructed sequences.

### Testing the Function of LUCA tRNA in the Translation System

We experimentally tested whether selected LUCA tRNAs can decode codons and support protein synthesis in vitro. Arc1 is the inferred nucleotide diphosphate kinase (NDK) of LACA (Akanuma et al. 2013). Shibue et al. (2018) used Arc1 variants to investigate the minimal number of amino acid species required to form a functional protein. One such mutant, Arc1ΔThr, lacks codons for threonine (Thr) and cysteine (Cys).

When a particular amino acid is excluded from the translation system, its corresponding codon cannot be decoded—even if the relevant ARS and tRNA are present—because aminoacylation does not occur. The uncharged tRNA cannot form a ternary complex with EF-Tu·GTP and is therefore not delivered to the ribosome. Even if the uncharged tRNA enters the A-site, translation is halted due to the absence of an attached amino acid. However, if an exogenous tRNA is introduced that carries a different amino acid and can decode the excluded codon, translation may resume.

To evaluate decoding ACN codons (where N = A, C, G, or T), we inserted a sequence encoding five consecutive test codons (ACT X 5, ACC X 5, ACA X 5, or ACG X 5) and ten His codons at the 5′ end of the *Arc1ΔThr* gene in the pET23a vector (see Materials and Methods and Fig. 1B). This design allows testing whether specific tRNA species can decode ACN codons and thereby enable synthesis of Arc1ΔThr.

As discussed above, the functionality of individual LUCA tRNA species in modern translation systems such as that of *E. coli* needs to be evaluated. As a first step, we selected two LUCA tRNA species—tRNA^Ala^ and tRNA^Ser^, for functional testing. It is well established that the anticodon sequences of tRNA^Ala^ and tRNA^Ser^ are not identity elements for their respective aminoacylation activities (Hou and Schimmel 1988; McClain et al. 1988; Park et al. 1989; Normanly et al. 1992). Therefore, altering the anticodon of tRNA^Ala^ and tRNA^Ser^ does not affect their alanylation or serylation efficiency, nor their amino acid specificity.

**Fig. 5:**
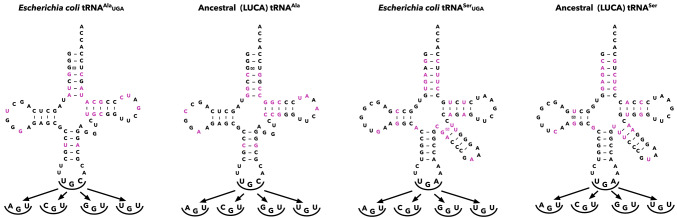
Cloverleaf structures of *E. coli* and LUCA tRNA^Ala^ and tRNA^Ser^. Mutants with altered anticodon sequences (AGU, CGU, GGU, and UGU) were used. The nucleotides differing between *E. coli* tRNA and LUCA tRNA are shown in magenta

To test whether LUCA tRNA^Ala^ and tRNA^Ser^ can function in the *E. coli* translation system and to explore a minimal anticodon set functional without posttranscriptional modifications, we synthesized *E. coli* and LUCA tRNA^Ala^ and tRNA^Ser^ variants with four different anticodon sequences (either of AGU, CGU, GGU, and UGU), resulting in 16 tRNA species in total (Fig. 5).

The 7 M Urea acrylamide gel electrophoresis confirmed that the in vitro transcripts of *E. coli* and LUCA tRNA^Ala^ and tRNA^Ser^ variants were successfully amplified as single bands (Supplementary Fig. S5). Native acrylamide gel electrophoresis also showed predominantly single bands, although faint additional bands were observed in some cases (data not shown). These results suggest that the tRNA transcripts generally adopt a single, stable structural form.

### In Vitro Translation of Arc1ΔThr Containing Test Codons Using *E. coli* and LUCA tRNA^Ala^ Anticodon Sequence Variants

**Fig. 6:**
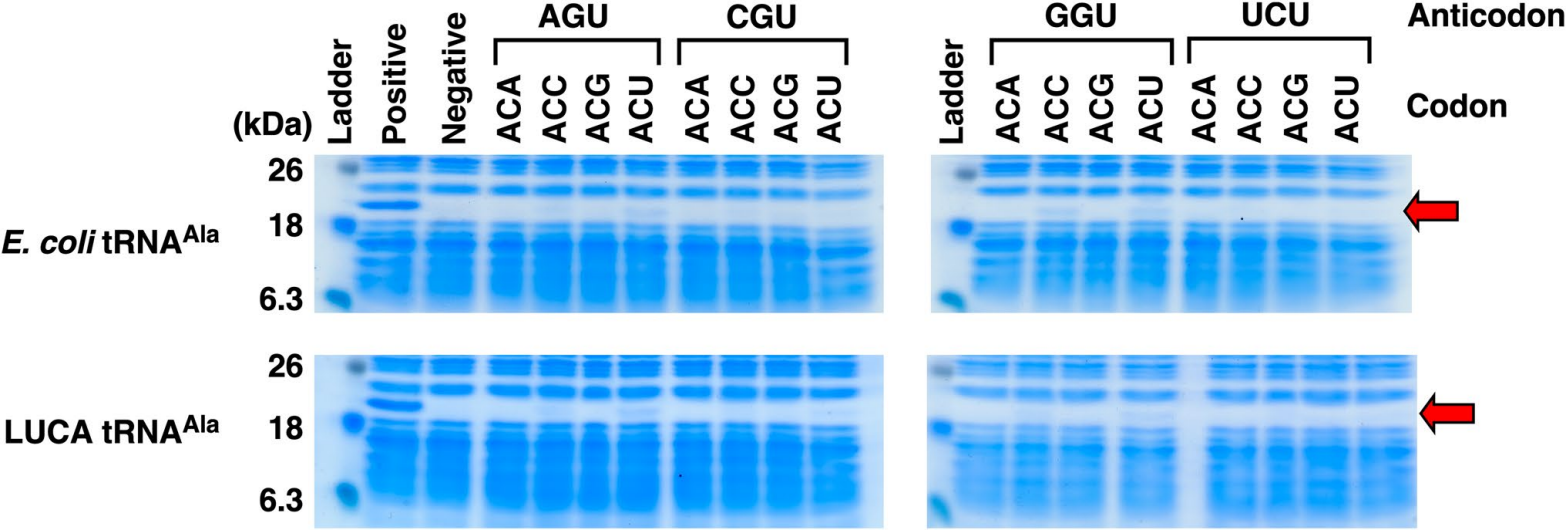
In vitro translation of Arc1ΔThr with test codon using *E. coli* and LUCA tRNA^Ala^ anticodon variants. Reactions were separated on SDS–PAGE gels (5% stacking and 15% separating) using Tris–glycine buffer. The upper panel shows reactions with *E. coli* tRNA^Ala^, and the lower panel shows reactions with LUCA tRNA^Ala^. The synthesized Arc1ΔThr protein was detected at the positions marked by arrows. The “Positive” control included all 20 amino acids (including threonine) and template plasmid (pET23a-ACU5-Arc1ΔThr), but no added tRNA transcript. The “Negative” control lacked threonine, template DNA, and tRNA. For reactions including a tRNA transcript, Thr was absent to assess the “suppressor activity” of the tRNA variants. The protein size marker used (“Ladder”) is the DynaMarker Protein MultiColor Ladder Marker, Stable II (BioDynamics Laboratory Inc., Tokyo, Japan)

We first tested decoding ACN codons using in vitro transcripts of *E. coli* and LUCA tRNA^Ala^ variants with anticodons UGU, CGU, AGU, and GGU. As shown in Fig. 6, the ACC and ACU codons were clearly decoded by *E. coli* tRNA^Ala^_AGU_ and tRNA^Ala^_GGU_. Similarly, LUCA tRNA^Ala^_AGU_ and tRNA^Ala^_GGU_ appeared to decode ACC and ACU codons as well. These results indicate that LUCA tRNA^Ala^ can function in the *E. coli* translation system.

**Fig. 7:**
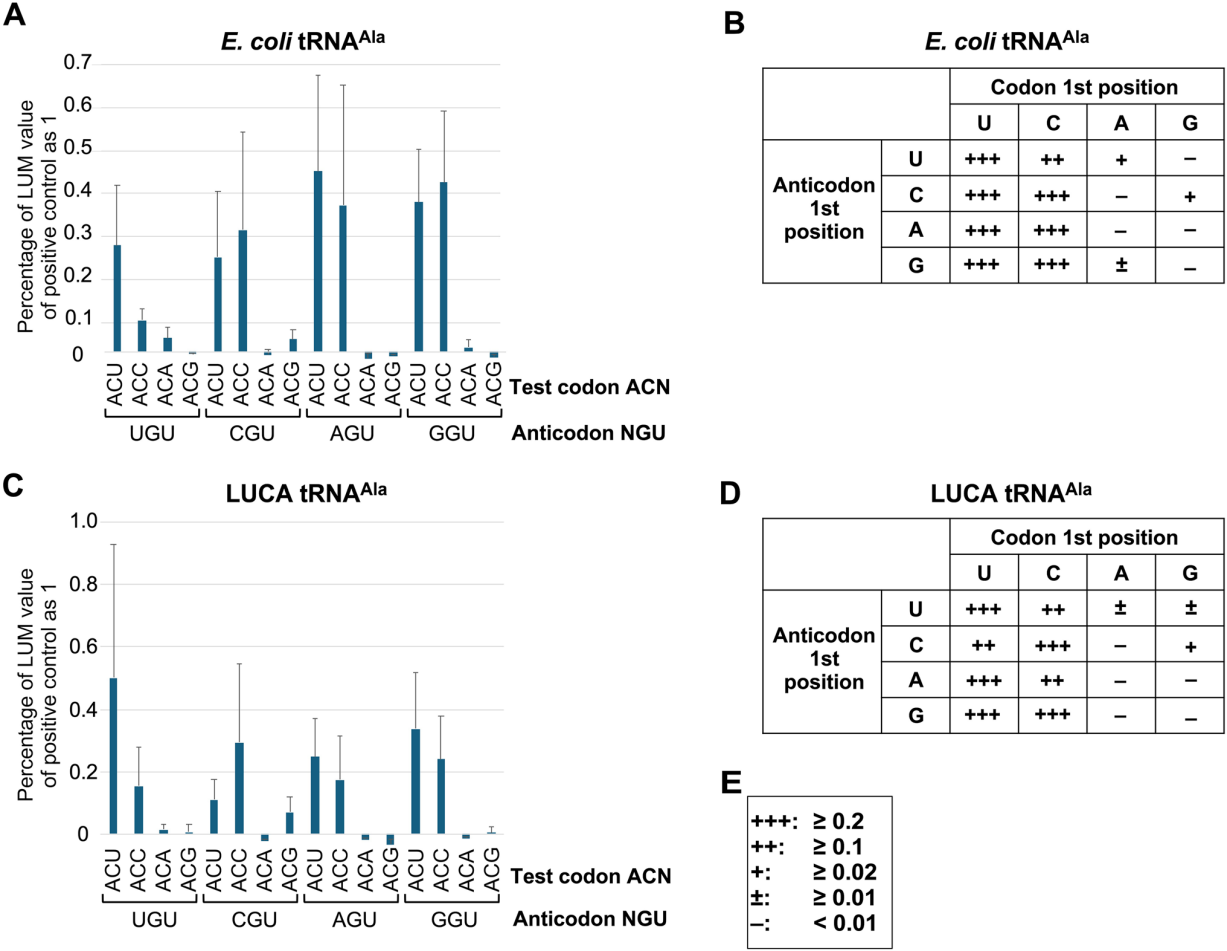
Relative translation efficiencies evaluated using the HiBiT system. Luminescence (LUM) values measured by the HiBiT system for reactions containing codon ACN and either *E. coli* tRNA^Ala^_NGU_ (**A**) or LUCA tRNA^Ala^_NGU_ (**C**). The values were normalized such that the positive and negative controls were 1 and 0, respectively. Bars represent the mean ± standard error (*N* = 4). Based on these results, codon recognition by the first base of the anticodon was evaluated for *E. coli* (**B**) and LUCA (**D**) using the criteria shown in panel (**E**).

Although faint bands corresponding to Arc1ΔThr were observed in other tRNA–codon combinations, their translation efficiency remained unclear. To evaluate translation activity with tRNA transcripts quantitatively with higher sensitivity we used HiBiT luminescence detection system (Dixon et al. 2016). The HiBiT tag sequence was added to the test genes as described in Materials and Methods section (Fig. 1B). Luminescence intensities were used to estimate the relative amounts of translated Arc1ΔThr (Fig. 7 and Supplementary Fig. S6).

In *E. coli* tRNA^Ala^ mutants (Fig. 7A and B), all four anticodon variants—UGU, CGU, AGU, and GGU—were able to decode ACU and ACC codons. *E. coli* tRNA^Ala^_UGU_ and tRNA^Ala^_CGU_ weakly decoded the ACA and ACG codon, respectively. Decoding of ACA codon by *E. coli* tRNA^Ala^_GGU_ was marginal. Similar decoding patterns were observed with the LUCA tRNA^Ala^ mutants (Fig. 7C and D), where all four variants could decode ACU and ACC codons. LUCA tRNA^Ala^_CGU_ also weakly decoded ACG codon, while the decoding of ACA by LUCA tRNA^Ala^_GGU_ was marginal.

In contrast to the tRNA^Ser^ mutants, the translation efficiencies of tRNA^Ala^ were substantially higher (Supplementary Fig. S6). For *E. coli* tRNA^Ser^ mutants (Supplementary Fig. S6A and S6B), tRNA^Ser^_AGU_ and tRNA^Ser^_GGU_ appeared to decode ACU and ACC codons, while tRNA^Ser^_CGU_ weakly decoded ACU and ACG codons. Among LUCA tRNA^Ser^ mutants (Supplementary Fig. S6C and S6D), tRNA^Ser^_AGU_ appeared to decode ACU codon, and tRNA^Ser^_UGU_, tRNA^Ser^_CGU_, and tRNA^Ser^_GGU_ were able to decode both ACU and ACC codons. Because experiments with tRNA^Ser^ mutants were conducted only once, these results should be interpreted with caution, and standard error bars are not provided.

The lower translation efficiency of both *E. coli* and LUCA tRNA^Ser^ variants compared to tRNA^Ala^ variants may be due to several factors. (1) Differences in the catalytic efficiency of aminoacylation between AlaRS and SerRS in the PURE*frex* working solution. (2) Structural stability of tRNA^Ala^ and tRNA^Ser^ in vitro transcripts. tRNA^Ala^ is a class I tRNA with a small variable loop (76 nucleotides including the CCA terminus), while tRNA^Ser^ is a class II tRNA with a large variable arm (88 nucleotides including the CCA terminus), which may reduce folding stability or translational efficiency. (3) Differences in the binding affinity of tRNA^Ala^ and tRNA^Ser^ to EF-Tu·GTP may also impact their delivery to the ribosome and overall translation efficiency.

The estimated abundance of native *E. coli* tRNA^Thr^ capable of decoding each ACN codon is as follows (Dong et al. 1996). ACU: tRNA^Thr^_GGU_ + tRNA^Thr^_UGU_ = 3.28%, ACC: tRNA^Thr^_GGU_ = 1.86%, ACA: tRNA^Thr^_UGU_ = 1.42%, and ACG: tRNA^Thr^_CGU_ + tRNA^Thr^_UGU_ = 2.26%. The concentration of total tRNA in the PURE*frex* 1.0 reaction solution in this study was calculated as approximately 46 µM. Therefore, the tRNA^Thr^ concentration decoding each ACN codon ranges from 0.6 to 1.5 µM. In the positive control with ACU-codon test gene, the decoding tRNA^Thr^ species were present at an estimated concentration of ~ 1.5 µM. By comparison, the concentration of the synthetic tRNA transcripts in the reaction solution was 0.8 µM—roughly half that of the native ACU-decoding *E. coli* tRNA^Thr^.

Direct comparison of translation efficiency between native *E. coli* tRNA^Thr^ and engineered *E. coli* or LUCA tRNA^Ala^ and tRNA^Ser^ (lacking post-transcriptional modifications) is inherently challenging. Nonetheless, several *E. coli* and LUCA tRNA^Ala^ variants, depending on their anticodon–codon pairing, exhibited translation efficiencies comparable to native *E. coli* tRNA^Thr^. In contrast, tRNA^Ser^ variants from both sources generally exhibited lower translation efficiency than tRNA^Ala^ variants. This may be attributed to the structural complexity of Class II tRNA^Ser^, including its large variable arm, which could lead to misfolded or translationally incompetent forms. Differences in the activity of AlaRS and SerRS in the PURE*frex* system may also have contributed.

### Wobble Basepairs Between LUCA tRNA and mRNA

**Fig. 8:**
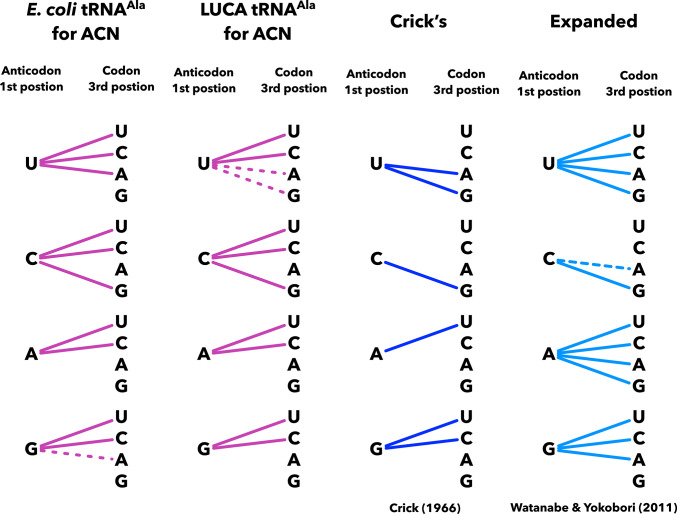
Recognition of third codon positions by the first position of the anticodon. Summary of decoding patterns for *E. coli* and LUCA tRNA^Ala^ based on the results shown in Fig. 6. Crick’s original wobble rules are from Crick (1966), and the expanded rules based on mitochondrial and mycoplasma systems are from Watanabe and Yokobori (2014)

Finally, we examined codon–anticodon pairing properties of LUCA tRNAs to infer characteristics of primitive decoding systems. As described above, the correspondence between the first anticodon position and the third codon position to the decoding of the ACN codon using tRNA^Ala^ variants in this study can be summarized as shown in Fig. 8. Our results demonstrate that the anticodons with U, C, A, or even G at the wobble position can decode codons with U and C in the third position. These observations do not strictly conform to either Crick’s original wobble hypothesis or the extended wobble rules (Table 1; Fig. 8).

**Table 1:** Expanded wobble rule proposed by Watanabe and Yokobori (2014) based on metazoan mitochondrial translation system compared with original wobble rule proposed by Crick (1966)

*N*_34_ of tRNA (1st position of tRNA)	*N*_3_ of codon (3rd position of codon)		
	Crick’s (Crick 1966)	Metazoan mitochondrial (Watanabe and Yokobori 2014)	
U	A, G	U, C, A, G	All tRNAs belonging to family box of almost all metazoans^a^
A	U	U, C, A, G	Nematode (*Ascaris suum*) tRNA^Arg^_ACG_^b^
G	C, U	U, C, A	Insect (*Drosophila melanogaster*) tRNA^Ser^_GCU_ for AGY/A codons^c^
C	G	A, G	Squid (*Loliogo bleekeri*) tRNA^Met^_CAU_^d^, Insect (*D. melanogaster*) tRNA^Lys^_CΨU_^e^

a: (Suzuki et al. 2011), b: (Watanabe et al. 1997), c: (Tomita et al. 1999), d: (Ohira et al. 2013), e: (Tomita et al. 1999)

Only cases of unmodified nucleotides at N_34_ of tRNA are shown

The ability of G at the first anticodon position to decode U and C at the third codon position is consistent with Crick’s wobble rule. Likewise, its potential to decode A at the third codon position agrees with the extended wobble framework (Watanabe and Yokobori 2014). In contrast, the observation that all tested anticodon variants—including those with U, C, and A at the first position, can decode both U and C codon endings is contradictory to conventional expectation.

According to the extended wobble rule, U at the first anticodon position is capable of decoding any of the four nucleotides at the third codon position (U, C, A, or G), a phenomenon reported in metazoan mitochondria as well as certain bacteria and archaea, such as *Mycoplasma capricolum* (Inagaki et al. 1995; Yokobori et al. 2013). However, in *M. capricolum* tRNA^Thr^_UGU_, the ACC codon is read less efficiently than ACU, ACA, and ACG (Inagaki et al. 1995). Similarly, studies have shown that U at the first anticodon position can misread U and C at the third codon position in vivo with relatively high efficiency (Manickam et al. 2014), especially in systems lacking competing tRNAs—as was the case in our in vitro condition. This is consistent with the fact that U_34_ can decode the third letter of the codons U and C.

Unmodified *E. coli* tRNA^Gly^_UCC_ has also been shown to decode all four GGN codons, whereas unmodified *E. coli* tRNA^Gly^_GCC_ and tRNA^Gly^_CCC_ fail to decode at least one of them (Kompatscher et al. 2023). Also, in Crick’s wobble rule, unmodified U at anticodon first position also decodes A and G in the codon third position. In our study, neither *E. coli* nor LUCA tRNA^Ala^ variants efficiently decoded A or G at the third codon position, even when U was present at the wobble position. This may reflect structural limitations in forming U_34_:A_3_ and U_34_:G_3_ pairs in our system (A_3_ and G_3_ are at the third codon position), possibly due to conformational constraints in the anticodon loop.

The case of C at the first anticodon position is particularly noteworthy. According to both Crick’s and the extended wobble rules, C at the first anticodon position should pair only with G at the third codon position. While rare exceptions suggest that A at the third codon position may be decoded via context-dependent mechanisms such as base 37 modification or loop sequence effects (Watanabe and Yokobori 2011, 2014), there is no known example of C at the first anticodon position decoding U or C at the third codon position. Our results, which show the apparent decoding of U and C at the third codon position by C at the first anticodon position, are not readily explained by established wobble rules and suggest the possibility of broader flexibility in decoding under simplified or primitive conditions.

Similarly, A at the first anticodon first position has been proposed to decode all four nucleotides at the third codon position in certain mitochondrial and bacterial systems with limited number of tRNA species (Inagaki et al. 1995; Watanabe and Yokobori 2014). In the case of *M. capricolum* tRNA^Thr^_AGU_, the ACA codon is read poorly so when compared with ACU, ACC, and ACG (Inagaki et al. 1995). Therefore, it is not surprising that the A at the first anticodon position decodes the U and C at the third codon position. Yet in our results, both *E. coli* and LUCA tRNA^Ala^ with A at the first anticodon position showed no decoding efficiency for A and G at the third codon position (Fig. 7). This again suggests that the intrinsic properties of the anticodon loop—rather than base pairing rules alone, may impose constraints on wobble flexibility.

It is also worth noting that the test constructs used in our experiments contained five consecutive instances of the same test codon. This repetition amplifies the effects of small differences in decoding efficiency: for example, a per-codon efficiency of 0.1, 0.5, and 0.9 results in cumulative translation success of only ~ 10^− 5^, 0.03, and 0.69 for five identical codons. Conversely, even low per-codon misreading rates (e.g., 1%) yield virtually no detectable full-length product over five codons (probability ≈ 10⁻^10^). Thus, our experimental system is highly sensitive to decoding accuracy and selectivity.

In summary, our results suggest that unmodified synthetic tRNAs, especially those reconstructed from (inferred) LUCA sequences, can exhibit broader wobble decoding flexibility than previously expected, particularly in simplified in vitro translation systems. These findings may reflect ancestral decoding mechanisms before the evolutionary emergence of anticodon modifications and refinements in codon discrimination.

## Concluding Remarks

In this study, we inferred a set of LUCA, LACA, and LBCA tRNAs for all 20 standard protein amino acids. In addition, we have also shown that LUCA tRNA^Ala^ and tRNA^Ser^, each bearing modified anticodons for ACN codons, can function within the *E. coli* translation system. Whether similar codon–anticodon pairing patterns are maintained in other codon boxes, and whether other LUCA tRNAs can operate effectively in the *E. coli* translational context, remains to be determined. A next goal of our future work is to reconstruct a translation system composed entirely of LUCA-derived tRNAs.

We believe that ASR based on molecular phylogenetics may enable the inference of pre-LUCA tRNA sequences, allowing deeper exploration of the evolutionary origins of the translational apparatus. The phylogenetic tree shown in Fig. 3 illustrates how such reconstructions may proceed. In parallel, progress has been made in reconstructing ARSs (Fournier et al. 2011; Fournier and Alm 2015), including our efforts to infer ARSs predating the LUCA (Furukawa et al. 2022; S. Yokobori, in preparation). Functional analysis of ancestral EF-Tu proteins has already been reported (Gaucher et al. 2008; Kacar et al. 2017a, b), and further studies of additional translational factors are likely to follow.

Importantly, the feasibility of replacing *E. coli* rRNA with that from other organisms (Asai et al. 1999; Kitahara et al. 2012), together with the in vitro reconstitution of the *E. coli* small ribosomal subunit (Tamaru et al. 2018), suggests that partial ancestral reconstruction of the ribosome is now within reach.

By combining ancestral components—tRNAs, ARSs, elongation factors, and ribosomal RNAs—derived from LUCA or earlier lineages, it may be possible to experimentally reconstitute primordial translation systems. Such reconstructions will provide valuable insights into how modern translation machinery evolved and became highly optimized.

## Supplementary Information

Below is the link to the electronic supplementary material.

Supplementary Material 1
