# Supplementary material for: Resurrecting tRNAs of the Last Universal Common Ancestor (Commonote) Toward Rebuilding the Ancient Translation System
Source: J Mol Evol. 2026 Jul 9;94(4):686–710. doi: 10.1007/s00239-026-10332-5 (PMC13433404; doi:10.1007/s00239-026-10332-5)
Supplement: Supplementary file 1 [file 239_2026_10332_MOESM1_ESM.pdf]

Resurrecting tRNAs of the Last Universal Common Ancestor (Commonote) toward Rebuilding the Ancient Translation System

Kei Yamanouchi, Hinata Moriya, & Shin-ichi Yokobori*
Department of Applied Life Sciences, School of Life Sciences, Tokyo University of Pharmacy and Life Sciences, Horinouchi, Hachioji, Tokyo 192-0392, Japan

*Corresponding Author

Email. yokobori@toyaku.ac.jp (Shin-ichi Yokobori)
Telephone No. +81-42-676-5035
Fax No. +81-42-676-7145

Supplementary Tables

Supplementary Table 1. List of genomes from which tRNA genes retrieved.

Archaea		
	Acidilobus saccharovorans 345_15	NC_014374
	Aciduliprofundum boonei T469	NC_013926
	Archaeoglobus fulgidus DSM_4304	NC_000917
	archaeon_AArc_Sl	NZ_CP025066
	Caldisphaera lagunensis DSM_15908	NC_019791
	Caldivirga maquilingensis IC_167	NC_009954
	Candidatus Aenigmarchaeota archaeon CG_4_9_14_3_um_filter_37_18	PFTQ01000001-PFTQ01000067
	Candidatus Aenigmarchaeota archaeon CG_4_10_14_0_8_um_filter_37_24	PFNF01000001-PFNF01000095
	Candidatus Aenigmarchaeota archaeon CG_4_10_14_3_um_filter_37_21	PFKR01000001-PFKR01000091
	Candidatus Bathyarchaeota archaeon	NZ_PIXS01000001-NZ_PIXS01000425
	Candidatus Bathyarchaeota archaeon	NZ_PIXT01000001-NZ_PIXT01000253
	Candidatus Caldiarchaeum subterraneum	BA000048
	Candidatus Diapherotrites archaeon CG11_big_fil_rev_8_21_14_0_20_37_9	PCWY01000001-PCWY01000087
	Candidatus Heimdallarchaeota archaeon AB_125	MEHH01000001-MEHH01000225
	Candidatus Heimdallarchaeota archaeon	PBWW01000001-PBWW01000076
	Candidatus Heimdallarchaeota archaeon	QMYZ01000001-QMYZ01000261
	Candidatus *Korarchaeum cryptofilum* OPF8	NC_010482
	Candidatus Lokiarchaeota_archaeon	QMYW01000001-QMYW01000309
	Candidatus Lokiarchaeota archaeon CR_4	MBAA01000001-MBAA01000239
	Candidatus *Mancarchaeum acidiphilum*	NZ_CP019964
	Candidatus *Methanomethylophilus alvus*	NZ_CP017686
	Candidatus *Nanopusillus acidilobi*	CP010514
	Candidatus *Nitrosopumilus sediminis*	NC_018656
	Candidatus Odinarchaeota archaeon LCB_4	MDVT01000001-MDVT01000009
	Candidatus Parvarchaeota archaeon	PSQC01000001-PSQC01000051
	Candidatus Thorarchaeota archaeon	QMYT01000001-QMYT01000559
	Candidatus Thorarchaeota archaeon	QMYU01000001-QMYU01000324
	Candidatus Thorarchaeota archaeon SMTZ_45	LRSM01000001-LRSM01000206
	Desulfurococcus amylolyticus 1221n	NC_011766

	Halobacterium salinarum NRC_1	NC_002607
	Haloferax volcanii DS2	NC_013967
	Halophilic archaeon DL31	NC_015954
	Methanocaldococcus jannaschii DSM_2661	NC_000909
	Methanocella arvoryzae MRE50	NC_009464
	Methanopyrus sp. KOL6	NZ_CP019470
	Methanosarcina mazei Go1	NC_003901
	Methanospirillum hungatei JF_1	NC_007796
	Methanothermobacter thermautotrophicus str. Delta_H	NC_000916
	Nanohaloarchaea archaeon SG9	CP012986
	Nitrososphaera viennensis EN76	NZ_CP007536
	Sulfurisphaera tokodaii str. 7	NC_003106
	Thermococcus kodakarensis KOD1	NC_006624
	Thermofilum pendens Hrk_5	NC_008698
	Thermoplasma acidophilum DSM_1728	NC_002578
Bacteria		
	Acetomicrobium mobile DSM_13181	NC_018024
	*Acidobacterium capsulatum*_ATCC_51196	NC_012483
	Akkermansia muciniphila ATCC_BAA_835	NC_010655
	Alteromonas naphthalenivorans	NC_015554
	Aminobacterium colombiense DSM_12261	NC_014011
	Anaerolineaceae bacterium oral taxon 439	NZ_CP017039
	Aquifex aeolicus VF5	NC_000918
	Bacillus subtilis subsp. *subtilis* str. 168	NC_000964
	Bacteroides fragilis YCH46	NC_006347
	Bifidobacterium longum DJO10A	NC_010816
	Brachyspira intermedia PWS_A	NC_017243
	Caldimicrobium thiodismutans	NZ_AP014945
	Caldisericum exile AZM16c01	NC_017096
	Campylobacter jejuni RM1221	NC_003912
	Chlamydia gallinacea 08-1274/3	NZ_CP015840
	Chlamydia psittaci 6BC	NC_015470
	Chlamydia trachomatis D_UW_3_CX	NC_000117

	Chlorobium limicola DSM_245	NC_010803
	Chthonomonas calidirosea T49	NC_021487
	Clostridium botulinum A str. ATCC_3502	NC_009495
	Coxiella burnetii Dugway 5J108-111	NC_009727
	Deferribacter desulfuricans SSM1	NC_013939
	Deinococcus radiodurans R1	NC_001263, NC_001264
	Delftia acidovorans SPH_1	NC_010002
	Denitrovibrio acetiphilus DSM_12809	NC_013943
	Desulfovibrio vulgaris DP4	NC_008751
	Desulfurispirillum indicum S5	NC_014836
	Desulfurobacterium thermolithotrophum DSM_11699	NC_015185
	Dictyoglomus thermophilum H_6_12	NC_011297
	Dictyoglomus turgidum DSM_6724	NC_011661
	Elusimicrobium minutum Pei191	NC_010644
	Endomicrobium proavitum	NZ_CP009498
	Erysipelothrix rhusiopathiae str. Fujisawa	NC_015601
	Escherichia coli str. K_12 substr. MG1655	NC_000913
	Fibrobacter succinogenes subsp. *succinogenes* S85	NC_013410
	Fimbriimonas ginsengisoli Gsoil_348	NZ_CP007139
	Flavobacterium psychrophilum JIP02_86	NC_009613
	Flexistipes sinusarabici DSM_4947	NC_015672
	Fusobacterium nucleatum subsp. *nucleatum* ATCC_25586	NC_003454
	Gemmatimonas aurantiaca T_27	NC_012489
	Gemmatimonas phototrophica	NZ_CP011454
	Gemmatirosa kalamazoonesis	NZ_CP007128
	Granulicella mallensis MP5ACTX8	NC_016631
	Haliangium ochraceum DSM_14365	NC_013440
	Haloplasma contractile SSD_17B	NZ_AFNU02000001-NZ_AFNU02000034
	Helicobacter pylori J99	NC_000921
	Ilyobacter polytropus DSM_2926	NC_014632
	Inordinaticella fortuita	NZ_NSKI01000001-NZ_NSKI01000077
	Kosmotoga olearia TBF_19_5_1	NC_012785
	Leptospira interrogans serovar. Lai str. 56601	NC_004342

	Leptospirillum ferrooxidans C2_3	NC_017094
	Leptotrichia buccalis C_1013_b	NC_013192
	Mariprofundus aestuarium	NZ_CP018799
	Mariprofundus ferrinatatus	NZ_CP018800
	Mesorhizobium australicum WSM2073	NC_019973
	Methylacidiphilum infernorum V4	NC_010794
	Methylobacillus flagellates KT	NC_007947
	Microcystis aeruginosa NIES_843	NC_010296
	Mycobacterium marinum M	NC_010612
	Mycoplasma genitalium G37	NC_000908
	Mycoplasma mobile 163K	NC_006908
	Myxococcus macrosporus	NC_015711
	Nautilia profundicola	NZ_CP026530
	Nautilia profundicola AmH	NC_012115
	Neisseria meningitidis MC58	NC_003112
	Nitrospira defluvii	NC_014355
	Nostoc flagelliforme CCNUN1	NZ_CP024785
	Onion yellows phytoplasma OY_M	NC_005303
	Opitutus terrae PB90_1	NC_010571
	Pasteurella multocida 36950	NC_016808
	Persephonella marina EX_H1	NC_012440
	Petrotoga mobilis SJ95	NC_010003
	Pseudomonas putida F1	NC_009512
	Rhodothermus marinus DSM_4252	NC_013501
	Roseiflexus sp. RS_1	NC_009523
	Selenomonas ruminantium subsp. *lactilytica* TAM6421	NC_017068
	Spirochaeta thermophila DSM_6192	NC_014484
	Streptococcus pneumoniae TIGR4	NC_003028
	Streptomyces coelicolor A3_2	NC_003888
	Synechococcus elongatus PCC_6301	NC_006576
	Terriglobus roseus DSM_18391	NC_018014
	Thermanaerovibrio acidaminovorans DSM_6589	NC_013522
	Thermoanaerobacter pseudethanolicus ATCC_33223	NC_010321

	Thermodesulfatator indicus DSM_15286	NC_015681
	Thermodesulfobacterium geofontis OPF15	NC_015682
	Thermodesulfovibrio yellowstonii DSM_11347	NC_011296
	Thermomicrobium roseum DSM_5159	NC_011959
	Thermotoga maritima MSB8	NC_021214
	Thermus thermophilus HB8	NC_006461
	Truepera radiovictrix DSM_17093	NC_014221
	Victivallales bacterium CCUG_44730	NZ_CP027227
	*Xanthomonas campestris*_pv. *vesicatoria* str. 85_10	NC_007508
	Zymomonas mobilis subsp. *mobilis* ZM4, ATCC_31821	NC_006526
Eukarya		
	Arabidopsis thaliana	NC_003070-NC_003071, NC_003074-NC_003076
	Caenorhabditis elegans	NC_003279_NC_003284
	Candida dubliniensis CD36	NC_012860-NC_012867
	Cryptomonas paramecium	NC_015329-NC_015331
	Cyanidioschyzon merolae strain 10D	NC_010127-NC_010146
	Dictyostelium discoideum AX4	NC_007087-NC_007092
	Drosophila melanogaster	NC_004354, NT_033777-NT_033779, NT_033777-NT_033779, NT_037436
	Hemiselmis andersenii	NC_009977-NC_009979
	Homo sapiens	NC_000001-NC_000023
	Kluyveromyces lactis	NC_006037-NC_006042
	Leishmania major strain Friedlin	NC_001905, NC_004916, NC_007244-NC_007273, NC_007284-NC_007287
	Malassezia sympodialis ATCC_42132	LT671821-LT671828
	Naegleria gruber strain NEG_M	NW_003162543-NW_003163326
	Plasmodium falciparum 3D7	NC_000521, NC_004318, NC_004325-NC_004331, NC_037280-NC_037284
	Saccharomyces cerevisiae S288C	NC_001133-NC_001148
	Schizosaccharomyces pombe	NC_003421, NC_003423-NC_003424
	Ustilago bromivora	LT558117-LT558140

Supplementary Table S2. List of oligonucleotides used in this study.

Names of Primers	Sequences
5' primer for PCR amplification of LUCA tRNA	TCATA-CTCCG-TCCGC-TGCGA-ACTCC
3' primer for PCR amplification of LUCA tRNA^{Ala}	T**G**GTG-GACCG-GCCGG-GATTT-GAACC-C (**G** = 2'-*O* methyl G)
3' primer for PCR amplification of LUCA tRNA^{Ser}	T**G**GCG-CAGAG-GGTGG-GATTC-GAACC-CA (**G** = 2'-*O* methyl G)
5' primer for PCR amplification of *E. coli* tRNA	CCATG-GTCTT-GGGGT-CCACG-TTTGC
3' primer for PCR amplification of *E. coli* tRNA^{Ala}	T**G**GTG-GAGCT-ATGCG-GGATC-GAACC-G (**G** = 2'-*O* methyl G)
3' primer for PCR amplification of *E. coli* tRNA^{Ser}	T**G**GCG-GTGAG-GGGGG-GATTC-GAACC-CC (**G** = 2'-*O* methyl G)
Arc1ΔThr_inverse_first_to_5end	CGTAG-TCGTG-GTTGT-AGAAG-AACCC-ATATG-TATAT-CTCCT-TCTTA-AAGTT
Arc1ΔThr_inverse_first_to_3end	TCTTC-TGGTG-GTATG-GAACG-TTCCT-TCGTT-ATGA-TCAA
Arc1ΔThr_inverse_second_to_5end	ATGAT-GATGC-GTAGT-CGTGG-TTGTA-GAAGA-ACCCA-TATGT-AT
Arc1ΔThr_inverse_second_to_3end	CACCA-TCATC-ATCAC-CATCA-TTCTT-CTGGT-GGTAT-GGAAC-GTTCC-TTCGT
Arc1ΔT_inverse_third_to_5end	CACCA-TCATC-ATCAC-CATCA-TTCTT-CTGGT-GGTAT-GGAAC-GTTCC-TT
Arc1ΔT_ACU5_inverse_third_to_3end[a]	ATGAT-GATG**A-GTAGT-AGTAG-TAGT**A-GAAGA-ACCCA-TATGT-ATATC-TCCTT
Arc1ΔT_ACC5_inverse_third_to_3end[a]	ATGAT-GATG**G-GTGGT-GGTGG-TGGT**A-GAAGA-ACCCA-TATGT-ATATC-TCCTT
Arc1ΔT_ACA5_inverse_third_to_3end[a]	ATGAT-GATG**T-GTTGT-TGTTG-TTGT**A-GAAGA-ACCCA-TATGT-ATATC-TCCTT
Arc1ΔT_ACG5_inverse_third_to_3end[a]	ATGAT-GATG**C-GTCGT-CGTCG-TCGT**A-GAAGA-ACCCA-TATGT-ATATC-TCCTT
Arc1ΔT_HiBiT-5end[b]	**CAC**AC-CAGAA-GAACC-GCCAG-AGCTA-CCCCA-TTCAA-CCAGT-TCTTC-GTCTT-TGAAG-AA
Arc1ΔT_HiBiT-3end[b]	**AGCGG-CTGGC-GGCTG-TTCAA-GAAGA-TTAGC-**TAATA-AGGAT-CCGAA-TTCGA-GCTCC-GTC

a: Test codons (ACA, ACC, ACG, or ACT) are shown with boldface letters.

b: HiBiT sequence is shown with boldface letters.

Supplementary Table S3. Comparison of sequence similarity between LUCA and *E. coli* tRNA genes.

	Number of *E. coli* tRNA genes (Number of *E. coli* tRNA genes with different sequences)	Similarity among *E. coli* tRNAs between different sequences (%)	Similarity between LUCA tRNA and *E. coli* tRNAs (%)
$tRNA^{Ala}$	5 (2)	98.0	67.1 – 74.0
$tRNA^{Arg}$	7 (4)	53.2 – 77.9	54.1 – 79.3
$tRNA^{Asn}$	4 (1)		74.0
$tRNA^{Asp}$	3 (1)		84.2
$tRNA^{Cys}$	1 (1)		72.2
$tRNA^{Gln}$	4 (2)	90.7	50.0 – 51.4
$tRNA^{Glu}$	4 (1)		76.7
$tRNA^{Gly}$	6 (3)	61.0 – 70.7	68.9 – 74.0
$tRNA^{His}$	1 (1)		68.9
$tRNA^{Ile}$	5 (3)	68.8 – 97.4	70.7 – 77.3
$tRNA^{Leu}$	8 (6)	58.6 – 98.9	55.3 – 64.7
$tRNA^{Lys}$	6 (1)		78.1
$tRNA_i^{Met}$	4 (2)	98.7	83.8 – 85.1
$tRNA_e^{Met}$	2 (1)		71.6
$tRNA^{Phe}$	2 (1)		83.6
$tRNA^{Pro}$	3 (3)	72.7 – 83.1	63.3 – 70.3
$tRNA^{Ser}$	5 (4)	55.9 – 77.4	61.1 – 71.8
$tRNA^{Thr}$	4 (4)	65.8 – 92.1	54.8 – 78.1
$tRNA^{Trp}$	1 (1)		72.6
$tRNA^{Tyr}$	3 (2)	97.6	67.9 – 70.2
$tRNA^{Val}$	7 (3)	70.1 – 92.2	70.3 – 74.3

Supplementary Figures.

Supplementary Figure S1. Detailed trees of 20 tRNA trees. Maximum likelihood trees were inferred with IQ-TREE. Archaeal, bacterial, and eukaryal OTUs are shown with magenta, blue, and tangerine, respectively.
(a) $tRNA^{Ala}$. 402 OTUs, 75 sites with indels, TIMe+R6 model, lnL = -9535.970.
(b) $tRNA^{Arg}$. 657 OTUs, 78 sites with indels, GTR+F+R6 model, lnL = -20061.633.
(c) $tRNA^{Asn}$. 181 OTUs, 77 sites with indels, K3P+G4 model, lnL = -4858.973.
(d) $tRNA^{Asp}$. 171 OTUs, 78 sites with indels, TIM3e+I+G4 model, lnL = -4338.350.
(e) $tRNA^{Cys}$. 199 OTUs, 78 sites with indels, GTR+F+I+G4 model, lnL = -5612.931.
(f) $tRNA^{Gln}$. 275 OTUs, 77 sites with indels, TIMe+R5 model, lnL = -8042.561.
(g) $tRNA^{Glu}$. 280 OTUs, 76 sites with indels, TIMe+R4 model, lnL = -7599.513.
(h) $tRNA^{Gly}$. 422 OTUs, 76 sites with indels, SYM+R6 model, lnL = -10435.870.
(i) $tRNA^{His}$. 153 OTUs, 79 sites with indels, TVM+F+I+G4 model, lnL = -5032.469.
(j) $tRNA^{Ile}$. 193 OTUs, 77 sites with indels, K3P+I+G4 model, lnL = -5074.578.
(k) $tRNA^{Leu}$. 730 OTUs, 99 sites with indels, TIMe+R7 model, lnL = -25165.739.
(l) $tRNA^{Lys}$. 290 OTUs, 76 sites with indels, TIM2e+R6 model, lnL = -7774.114.
(m) $tRNA^{Met}$. 414 OTUs, 78 sites with indels, SYM+R6 model, lnL = -10810.144.
(n) $tRNA^{Phe}$. 144 OTUs, 75 sites with indels, TIM+F+I+G4 model, lnL = -3817.194.
(o) $tRNA^{Pro}$. 393 OTUs, 76 sites with indels, TIMe+R5 model, lnL = -10191.882.
(p) $tRNA^{Ser}$. 617 OTUs, 105 sites with indels, SYM+R6 model, lnL = -22863.940.
(q) $tRNA^{Thr}$. 447 OTUs, 77 sites with indels, TIMe+R6 model, lnL = -12188.208.
(r) $tRNA^{Trp}$. 145 OTUs, 77 sites with indels, TIMe+R4 model, lnL = -4693.030.
(s) $tRNA^{Tyr}$. 195 OTUs, 90 sites with indels, TVMe+R5 model, lnL = -5786.096.
(t) $tRNA^{Val}$. 406 OTUs, 775 sites with indels, GTR+F+R5 model, lnL = -10929.490.

(a) tRNA^{Ala} tree.

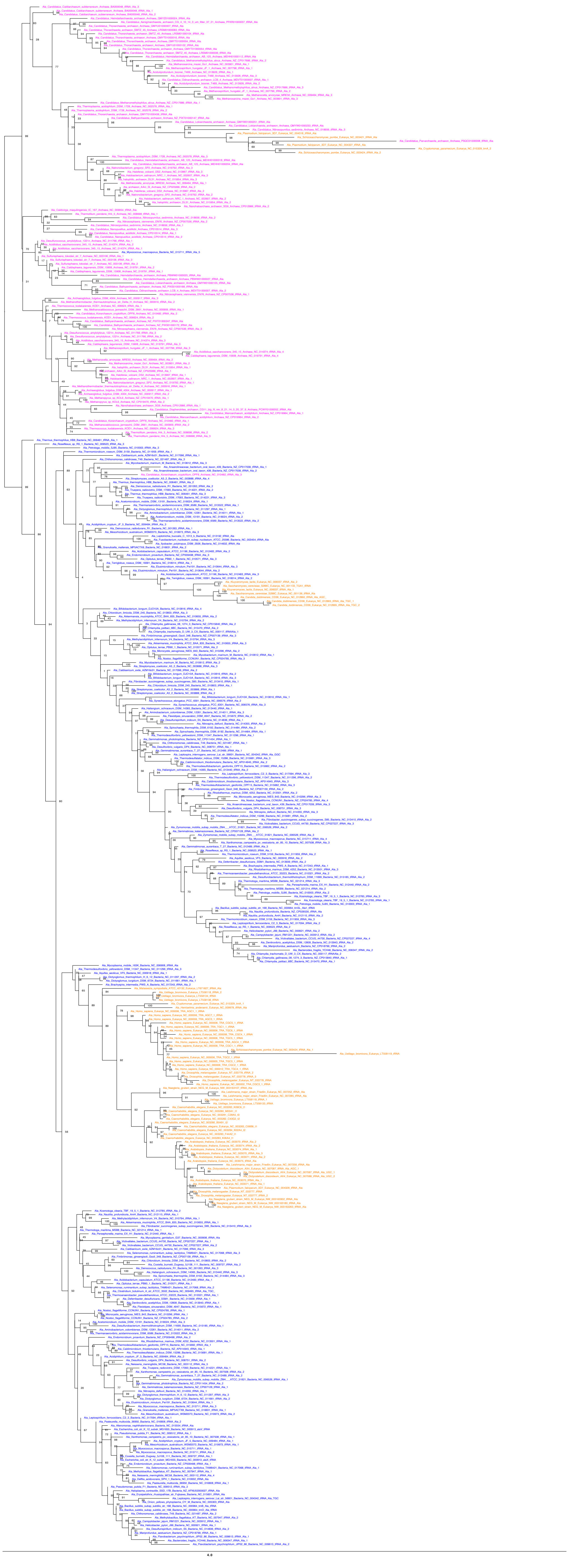

(b) tRNA^{Arg} tree.

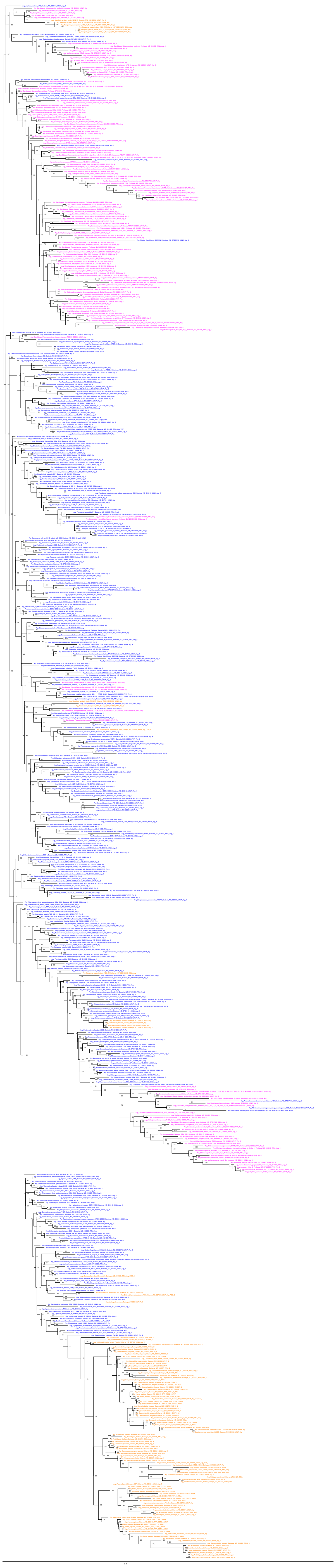

(c) tRNA^{Asn} tree.

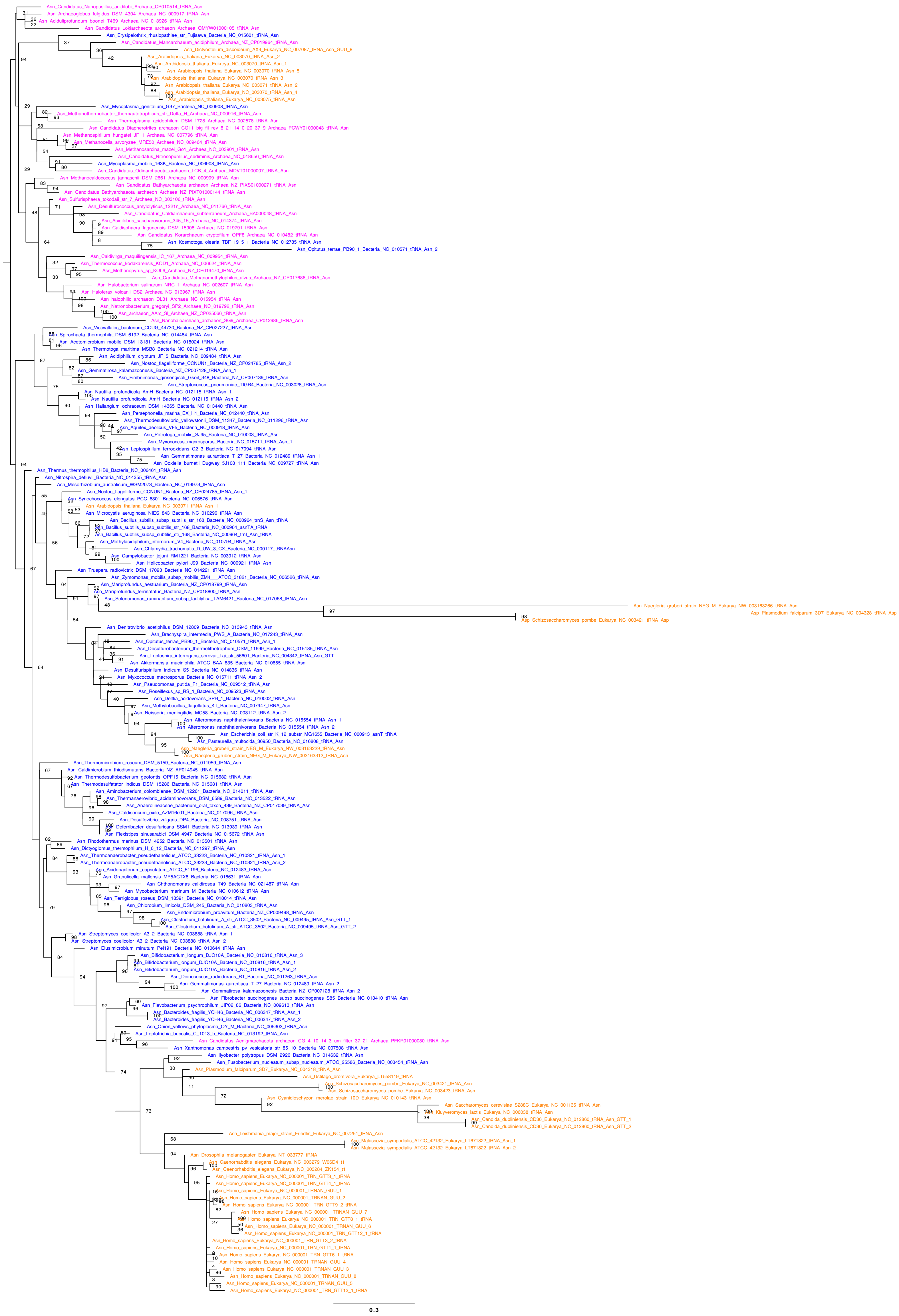

(d) tRNA^{Asp} tree.

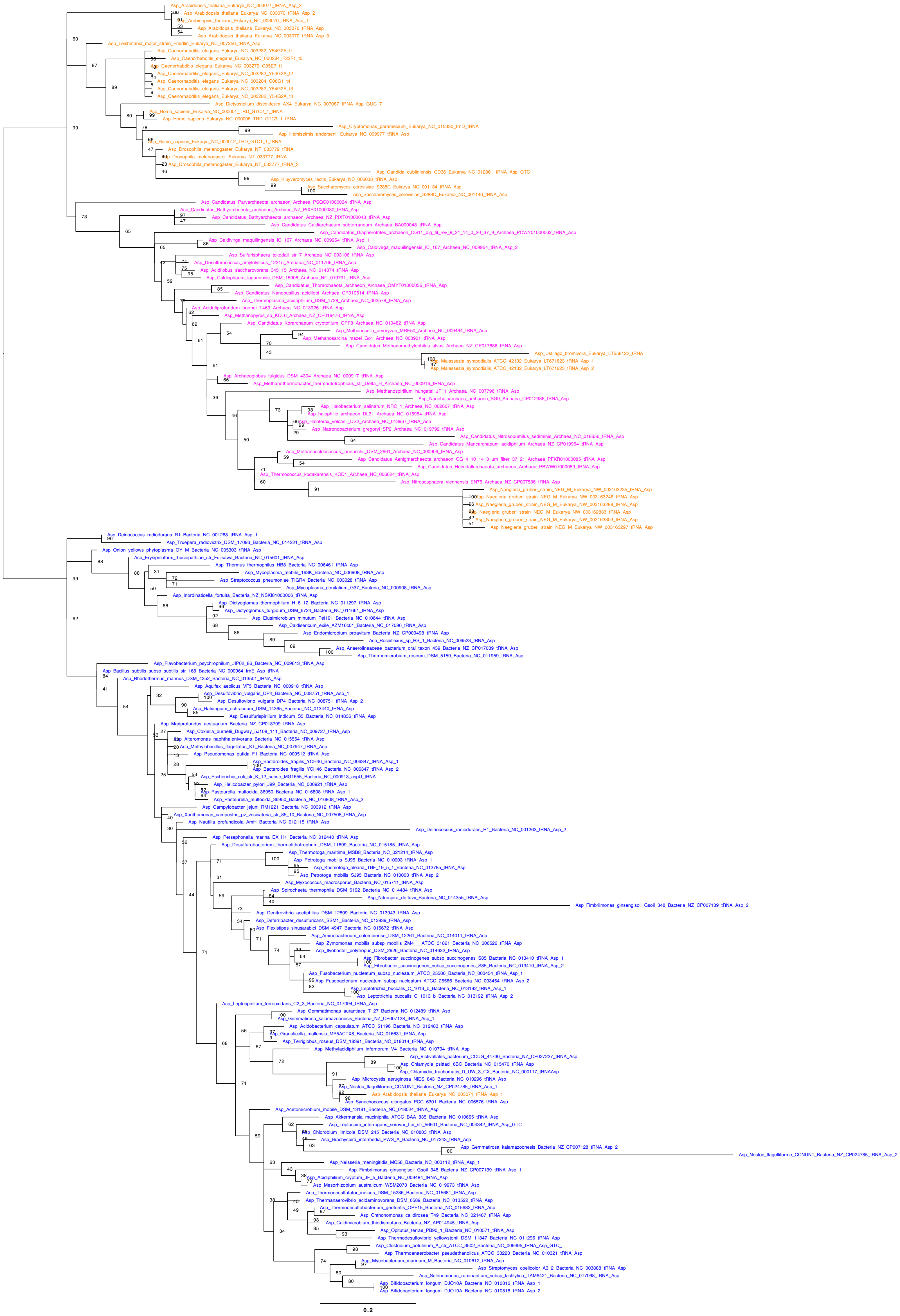

(e) tRNA^{Cys} tree.

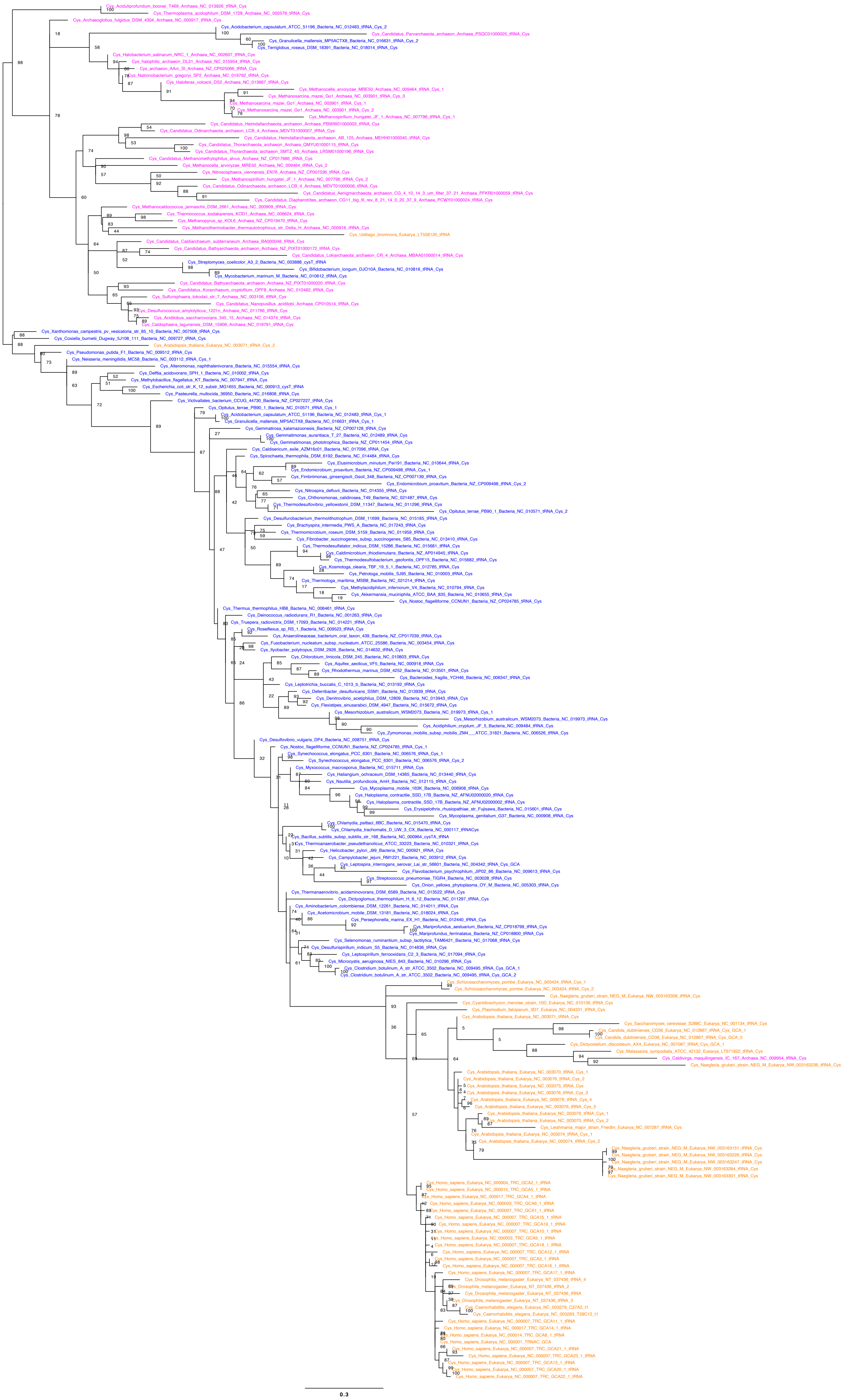

(f) tRNA^{Gln} tree.

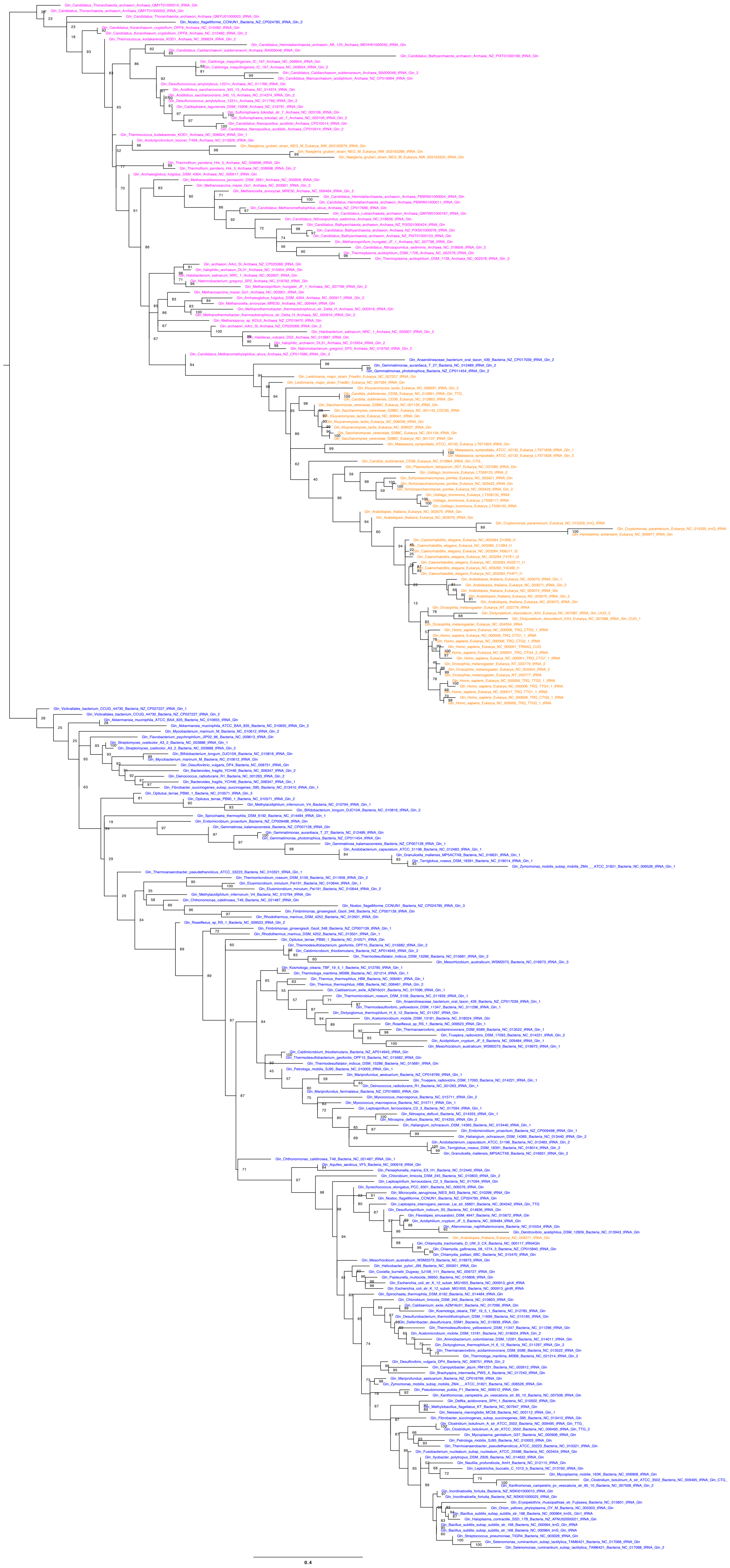

(g) tRNA^{Glu} tree.

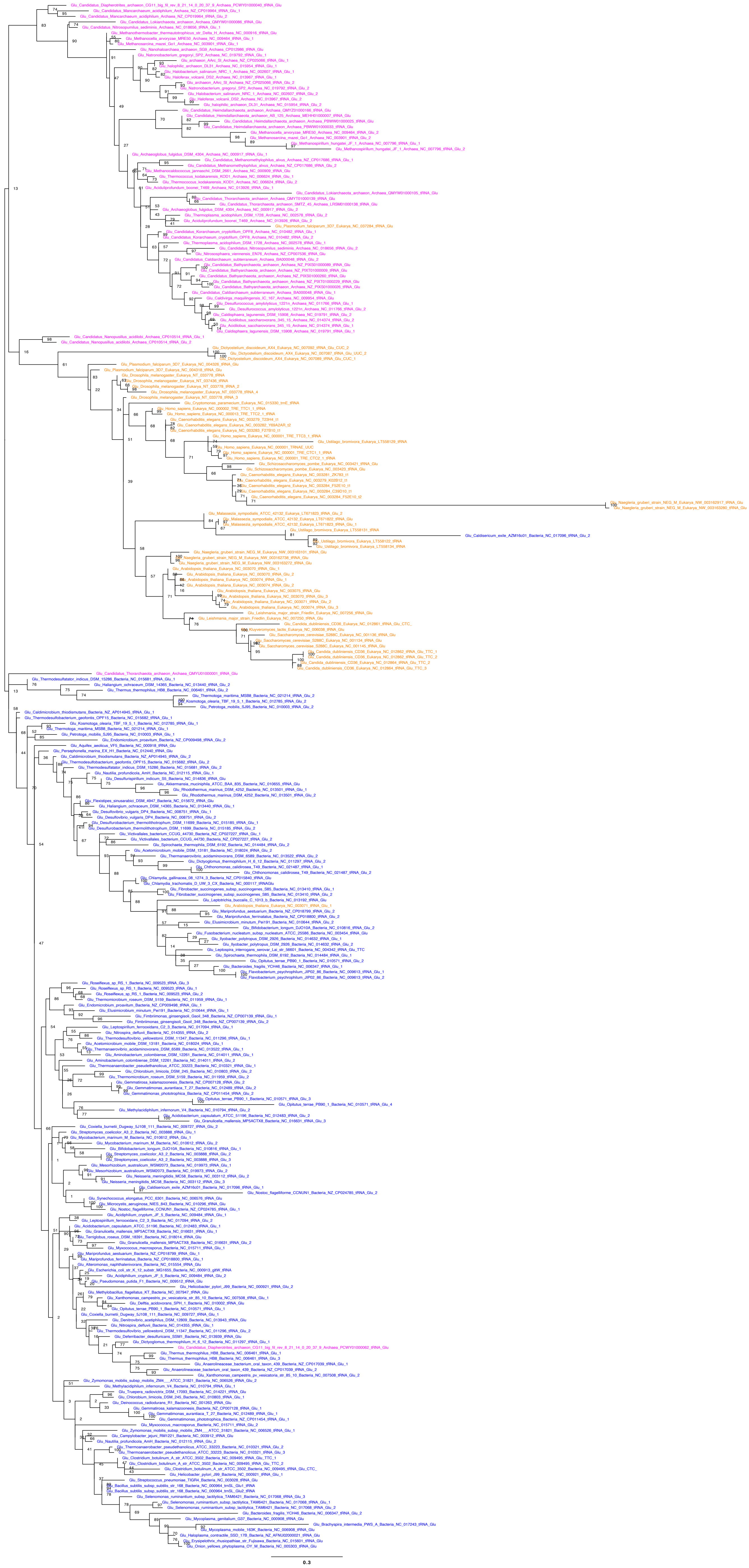

(h) tRNA^{Gly} tree.

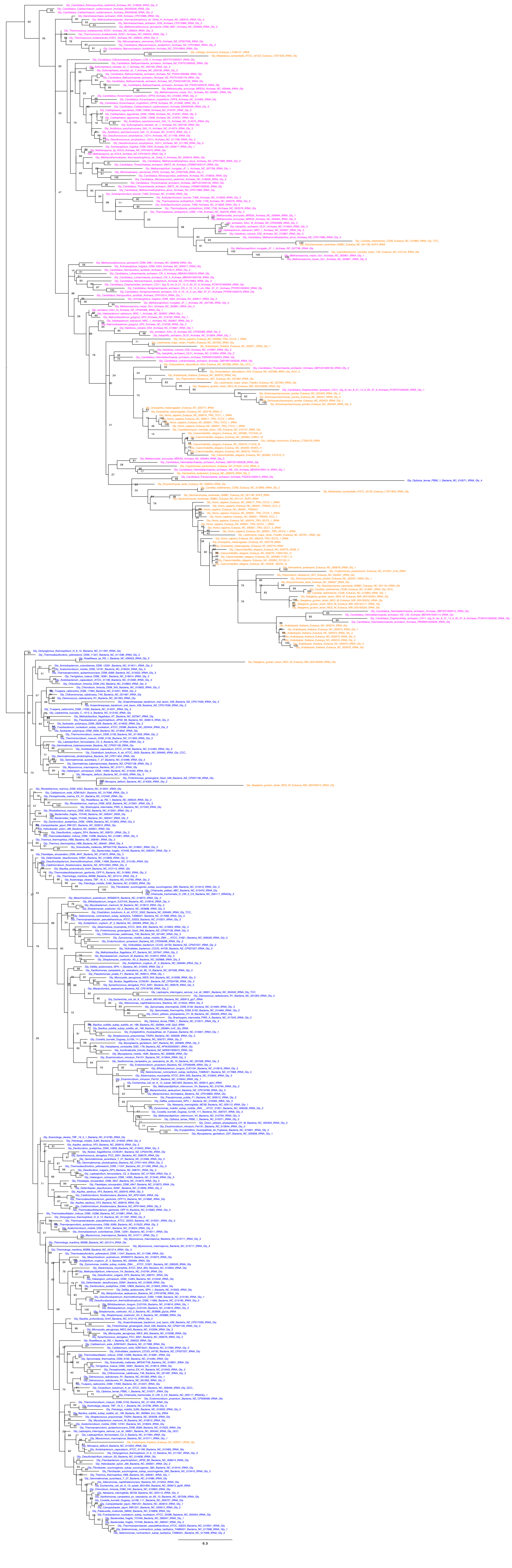

(i) tRNA^{His} tree.

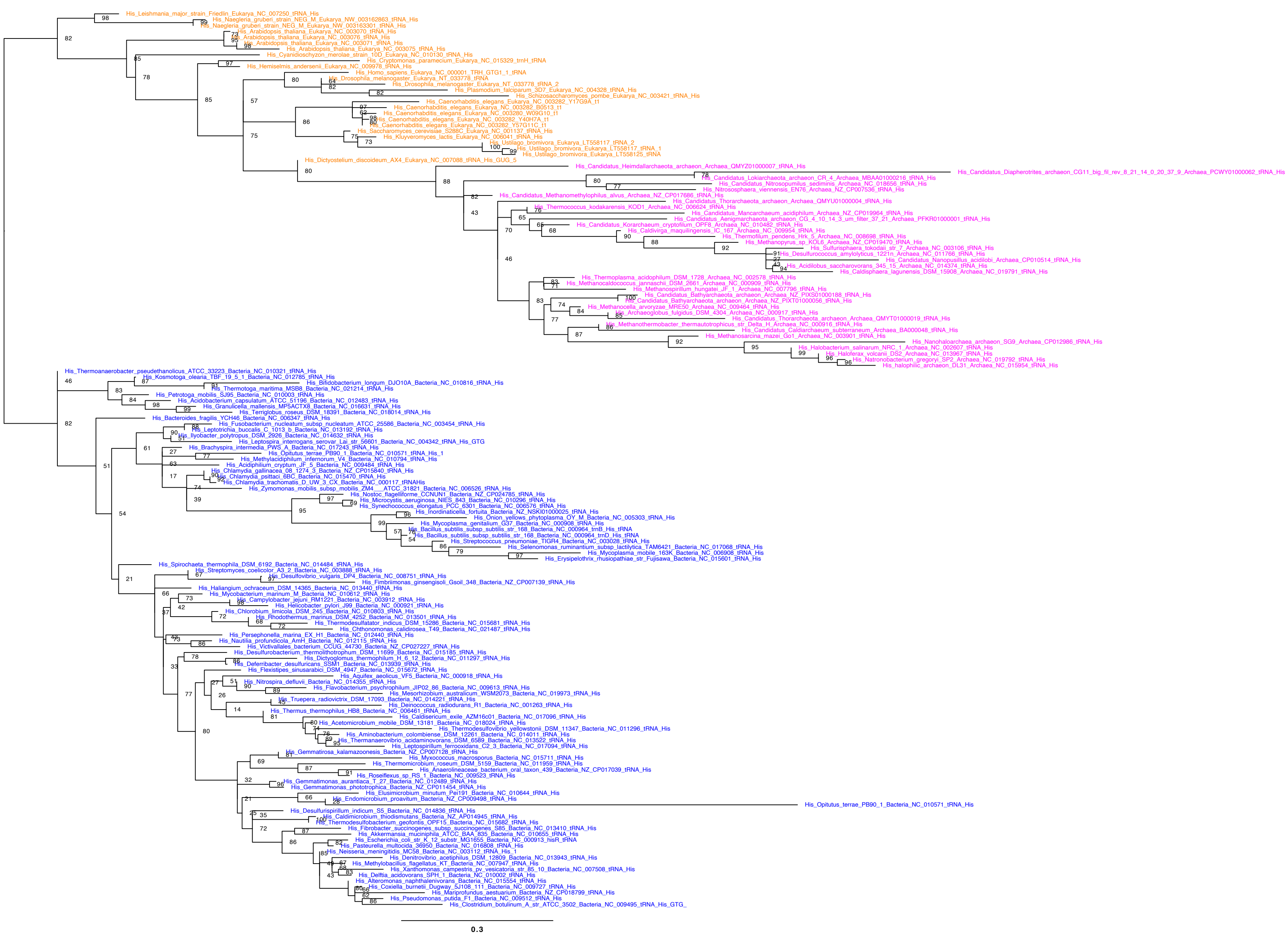

(j) $tRNA^{Ile}$ tree.

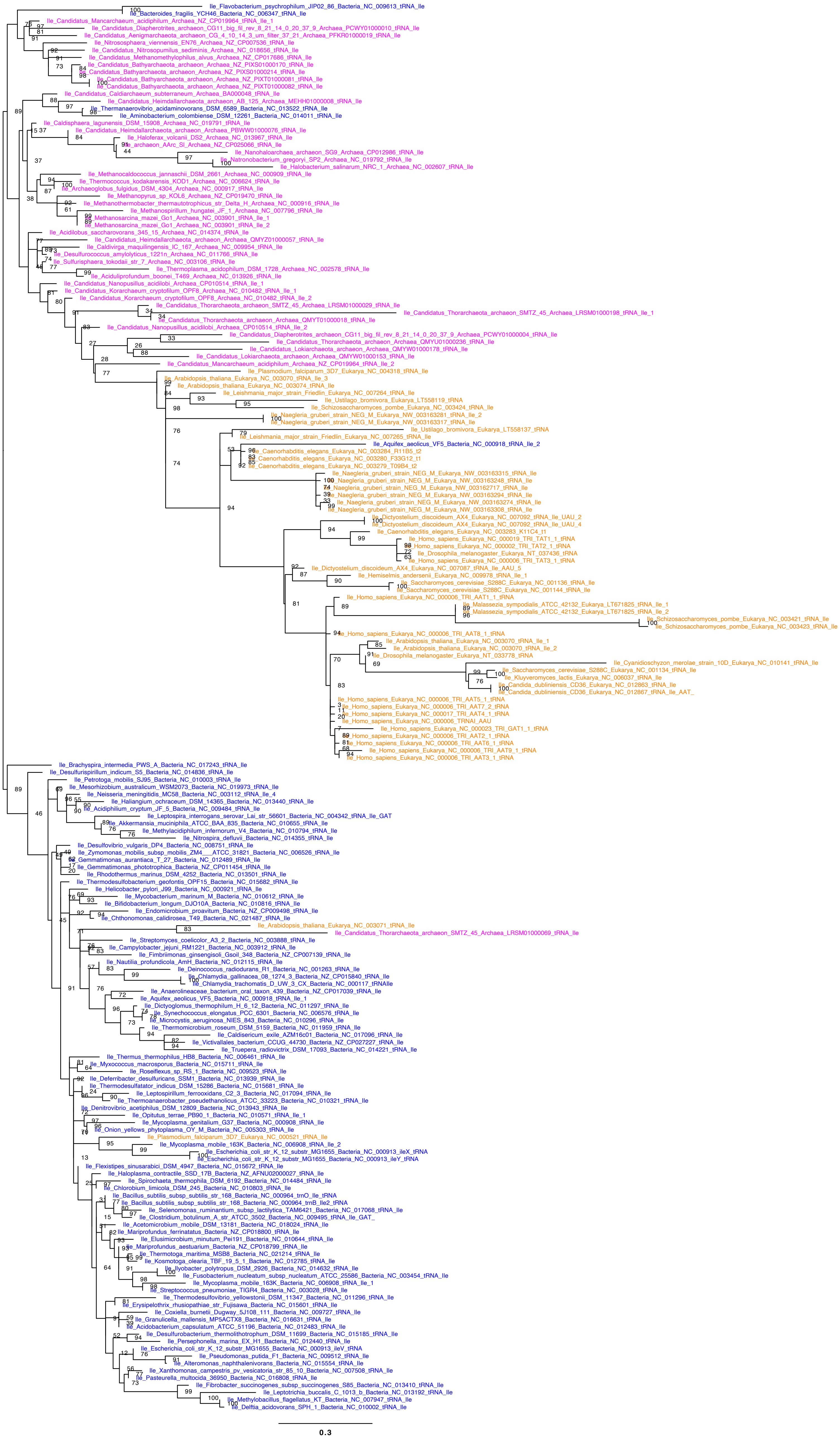

(k) $tRNA^{Leu}$ tree.

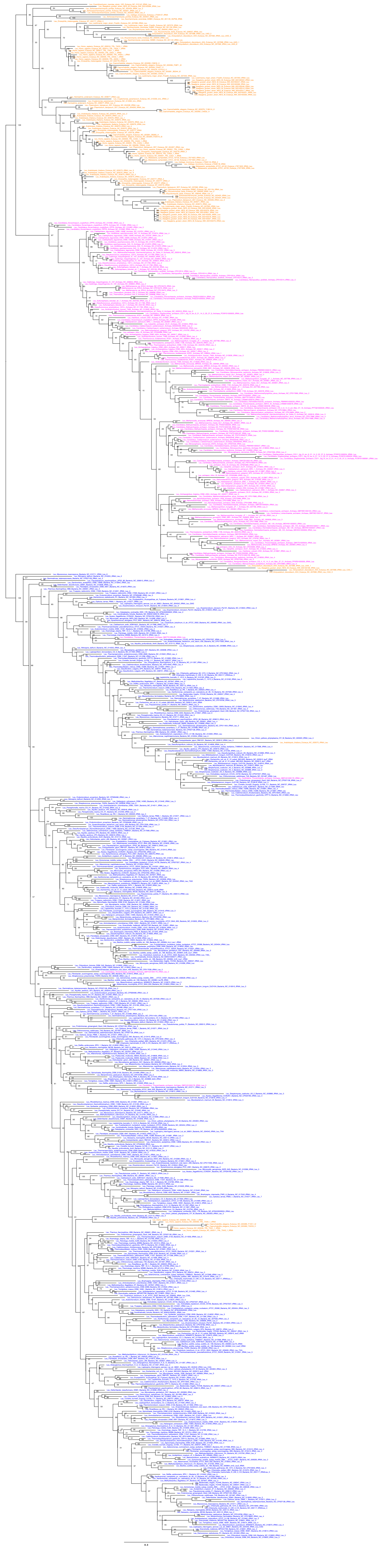

tree.

(l) $tRNA^{Lys}$

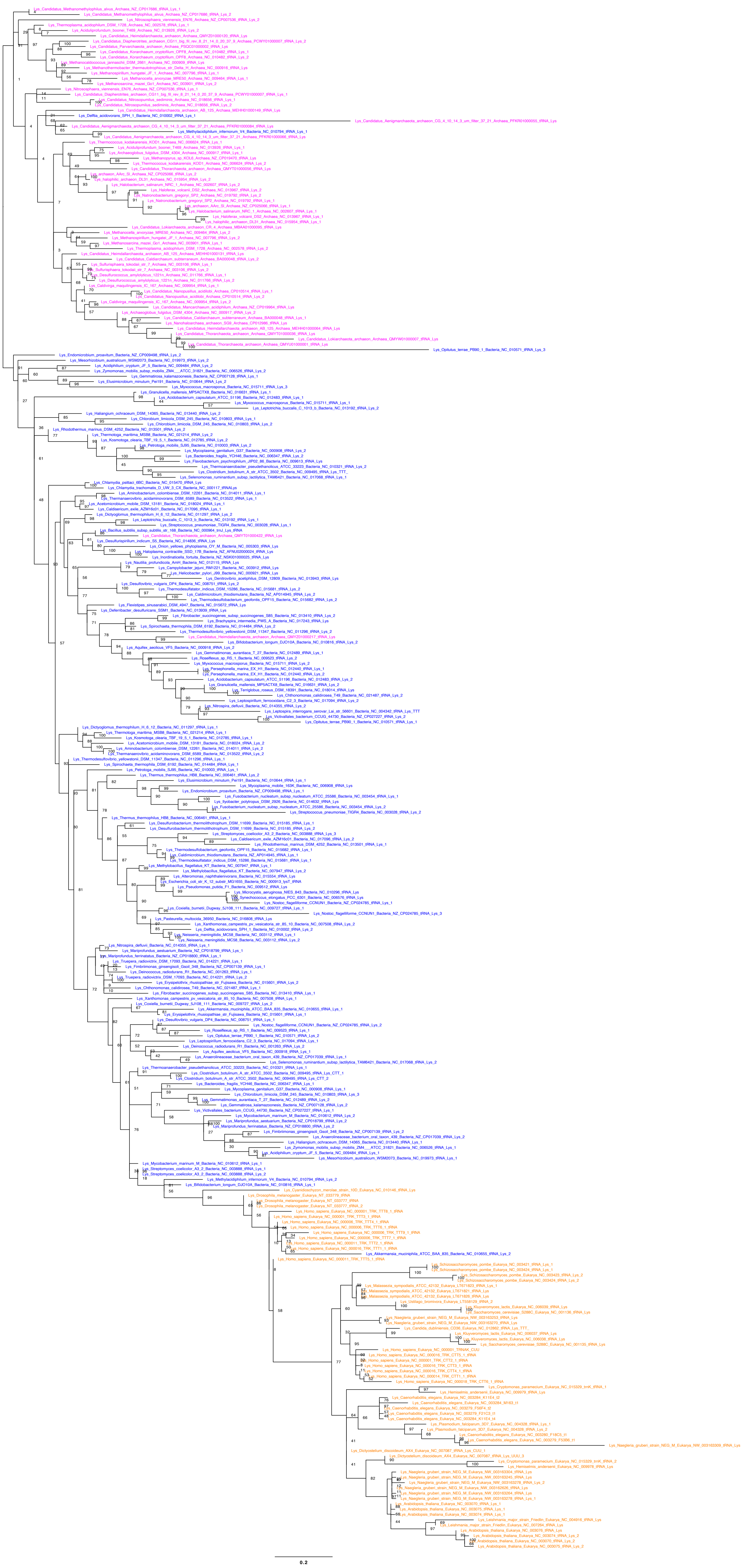

(m) tRNA^{Met} tree.

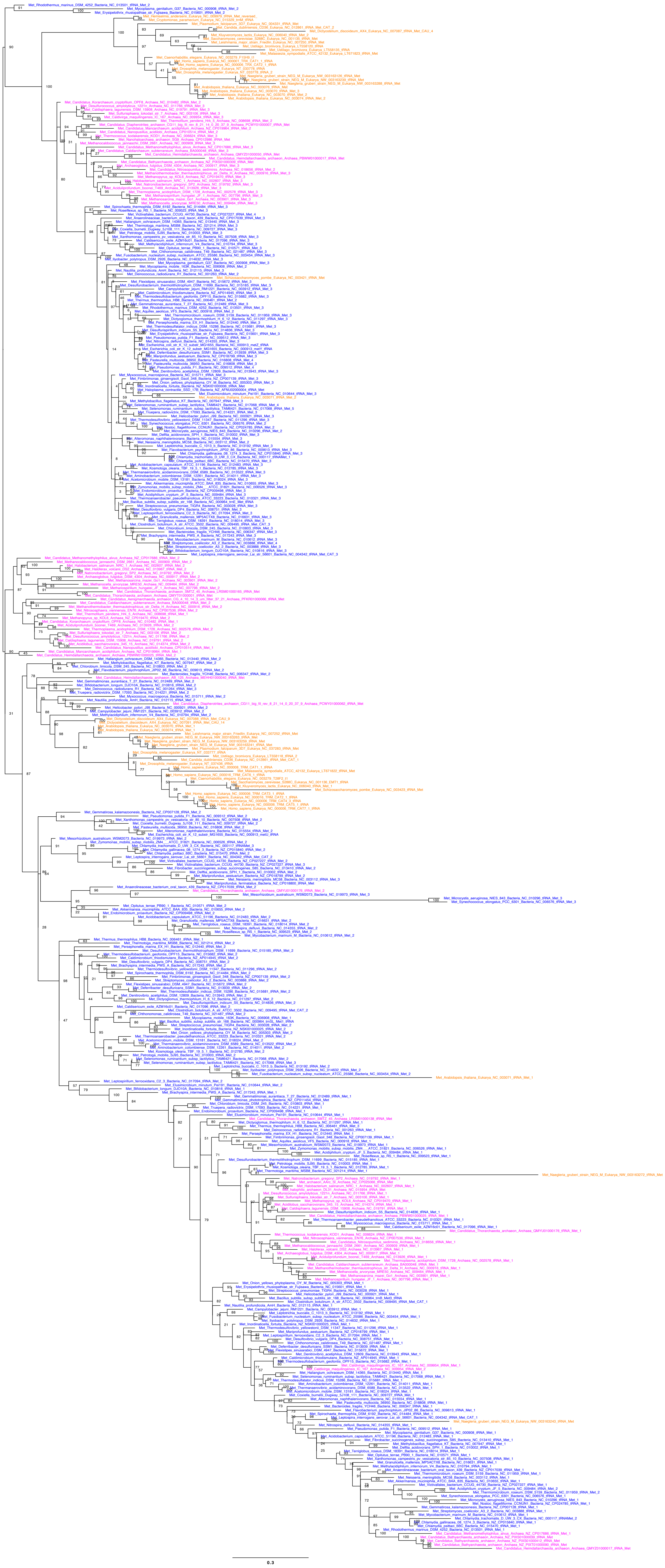

(n) tRNA^{Phe} tree.

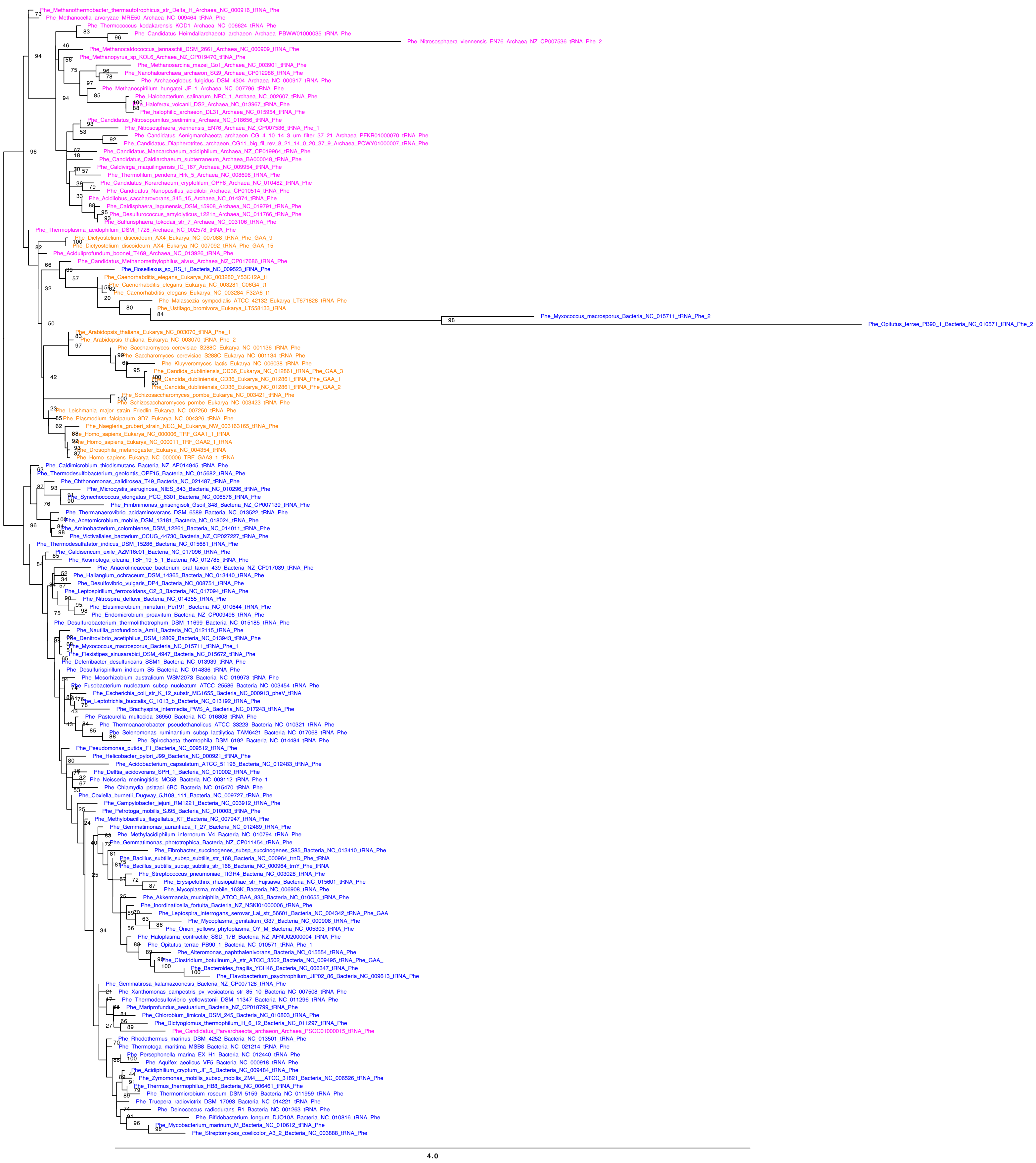

(o) tRNA^{Pro} tree.

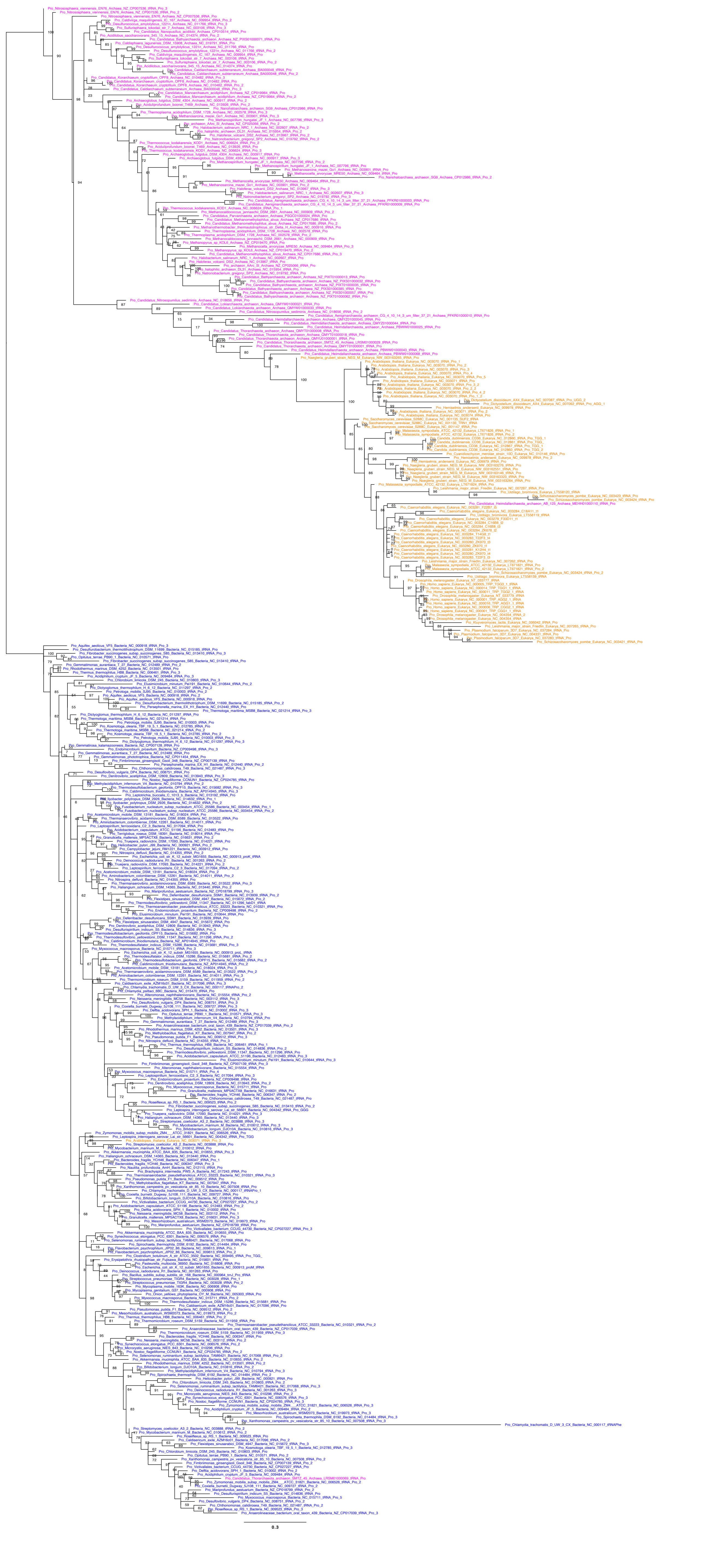

(p) $tRNA^{Ser}$ tree.

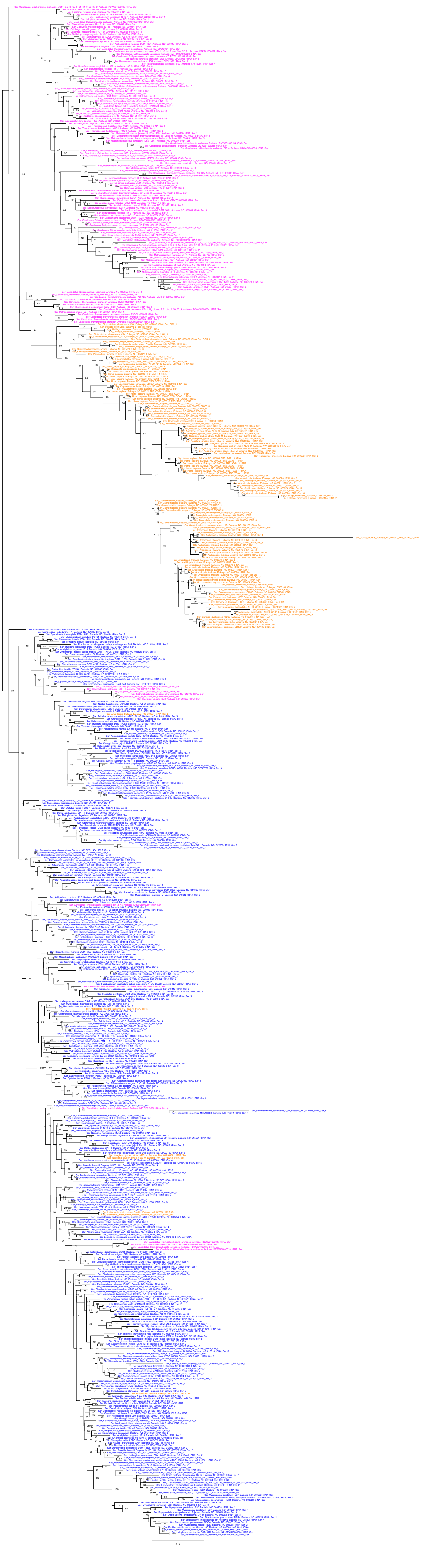

(q) tRNA^{Thr} tree.

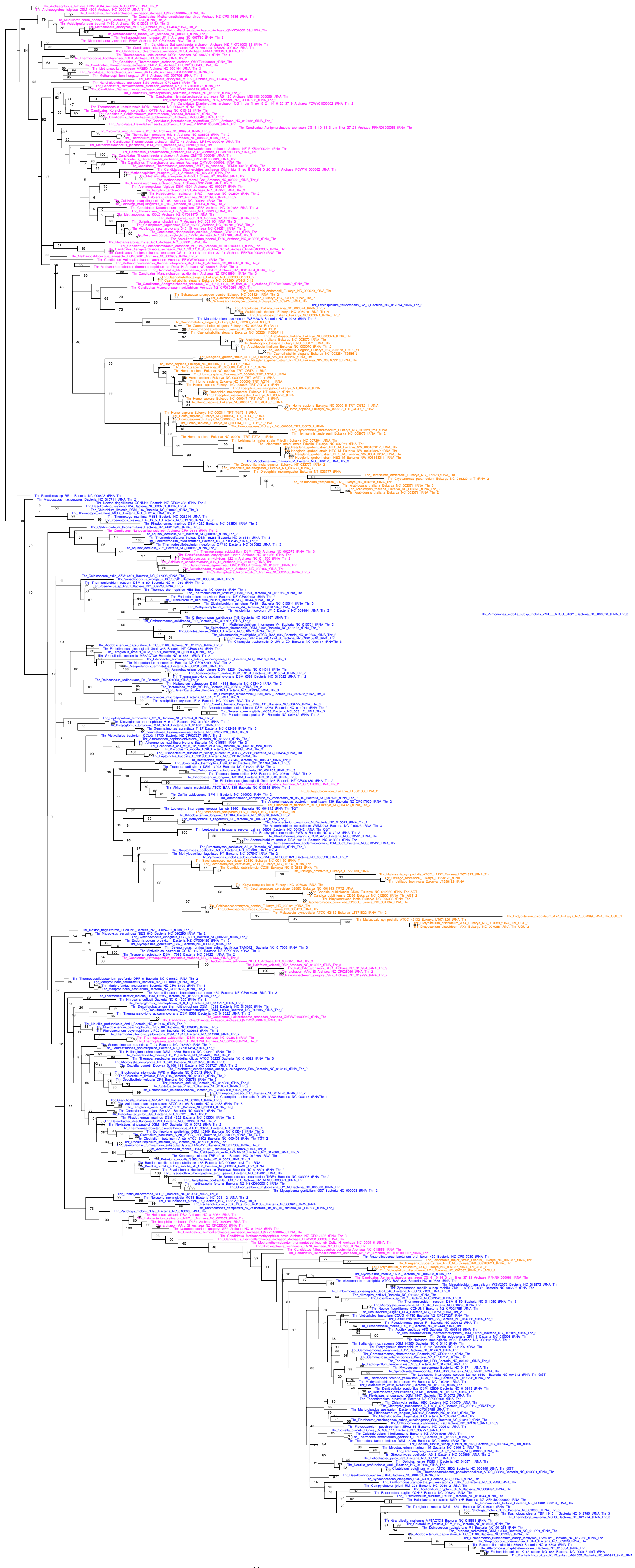

(r) tRNA^{Trp} tree.

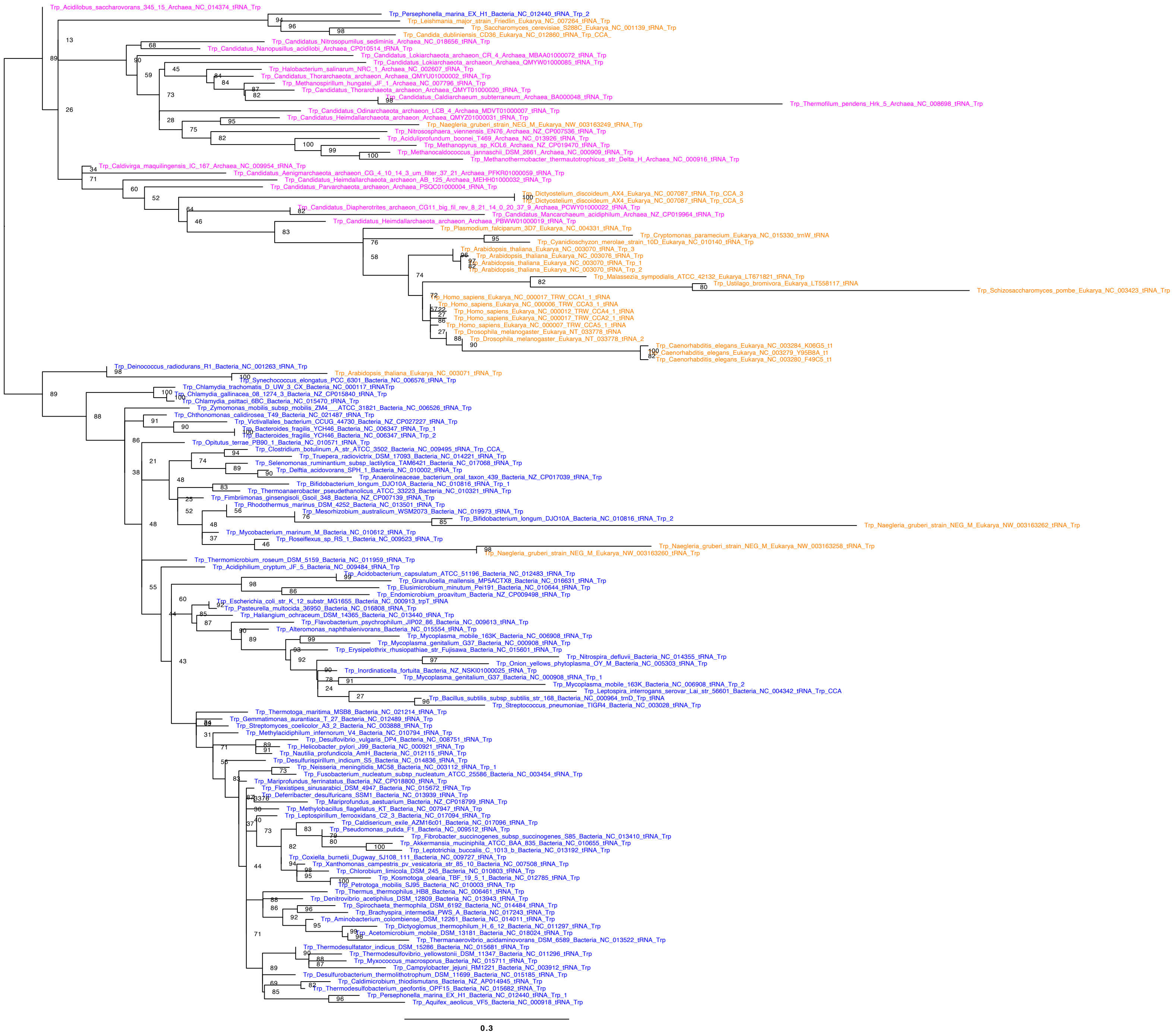

(s) tRNA^{Tyr} tree.

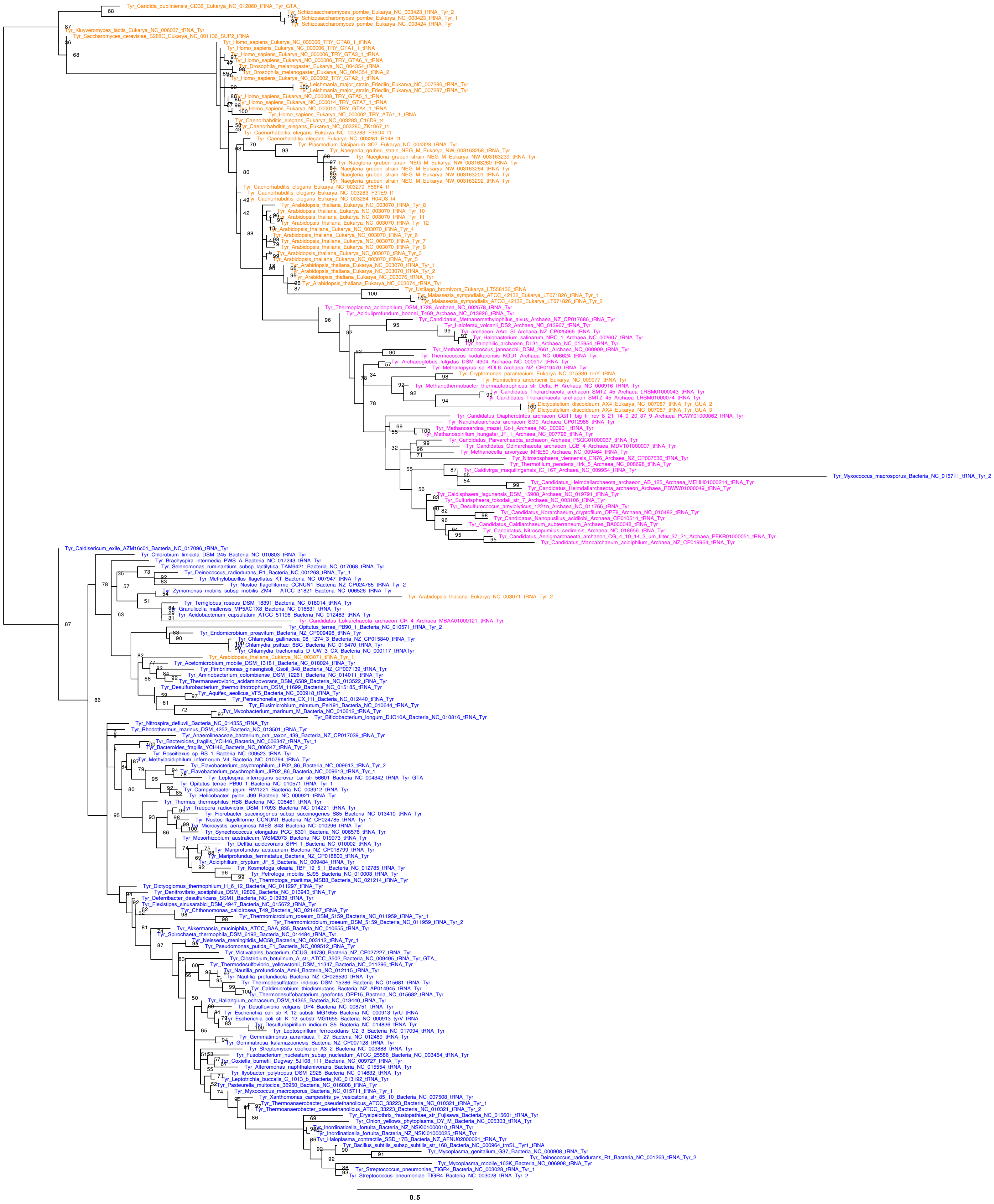

(t) tRNA^{Val} tree.

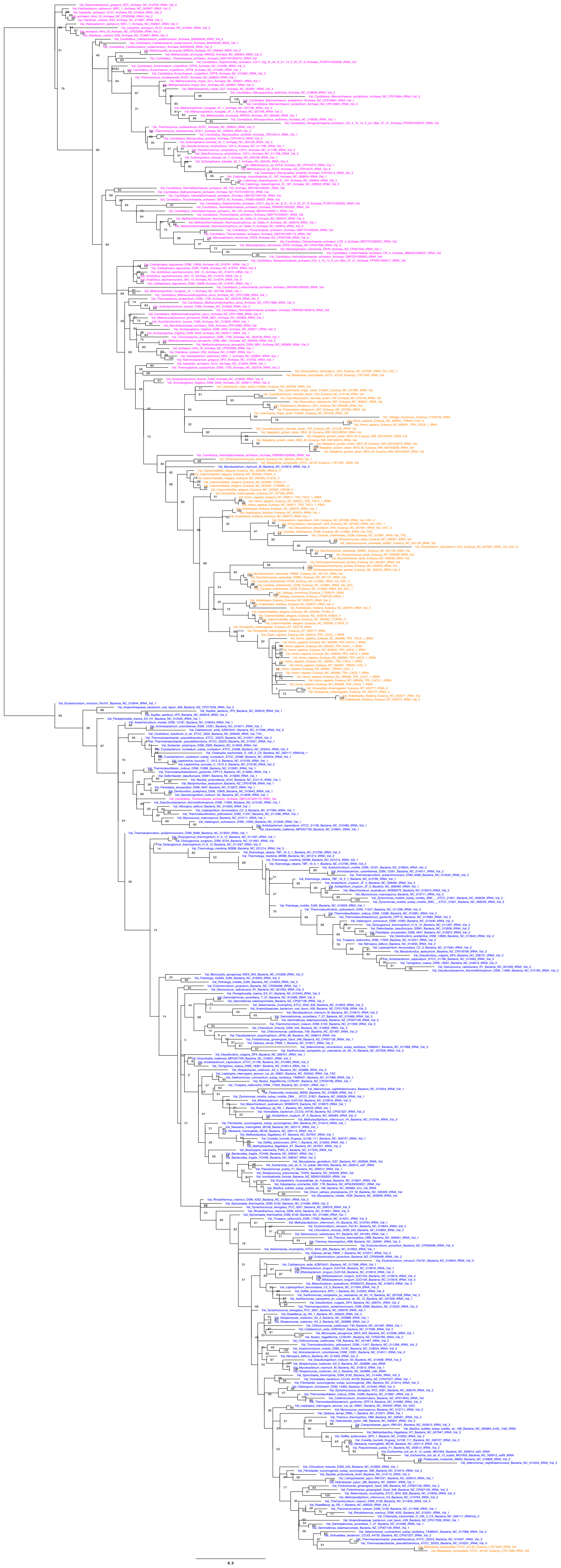

Supplementary Figure S2. Comparison of maximum probability at each site of LACA and LBCA tRNAs. Sites with maximum probability < 0.9 for any state are shown in yellow, and sites with maximum probability < 0.5 for any state are shown in red. The sequences were inferred based on the trees shown in Supplementary Fig. S1.

Ala	LACA	GGGCCGGTAGCTCAGCCTGGA-AGAGCGCCCGCTTGGCATGCGGGAGGTCCCGGGTTCAAATCCCGGCCGGTCCA
	LBCA	GGGGCGGTAGCTCAGAT-GGG-AGAGCGCCCGCTTTGCACGCGGGAGGTCCGGGGTTCAAATCCCCGCCGCTCCA
Arg	LACA	GGGCCCGTAGCTCAGCT-GGAT-AGAGCAACGGACTTCTAATCCGTAG--GTCGGGGGTTCGAATCCCCCCGGGCCCG
	LBCA	GGGCCCGTAGCTCAGCT-GGAT-AGAGCAACGGACTTCTAATCCGTAG--GTCGGAGGTTCGAATCCTCCCGGGCCCG
Asn	LACA	GCCGCCGTAGCTCAGTT-GGT--AGAGCAGCCGGCTGTTAACCGGTAG-GTCGCAGGTTCGAGTCCTGCCGGCGGCG
	LBCA	TCCGCCGTAGCTCAGTT-GGT--AGAGCAGCCGGCTGTTAACCGGTAG-GTCGCAGGTTCGAGTCCTGCCGGCGGAG
Asp	LACA	GCCTCGGTAGTATAGT---GGTT--AGTATACCCGCCTGTCACGCGGGAG-ACCCGGGTTCAATTCCCGGCCGGGGCG
	LBCA	GGTCCGGTAGTGTAGT---GGTT--AGCATGCCTGCCTGTCACGCAGGAGGTCGCGGGTTCAAGTCCCGTCCGGACCG
Cys	LACA	GGCCGGGTGGCAGAGT--GGT---TATGCGGCGGACTGCAGATCCGCTT-TACGCCGGTTCGAATCCGGCCCCGGCCT
	LBCA	GGCCGGGTGGCAGAGT--GGT---TATGCAGCGGACTGCAAATCCGCGT--ACGCCGGTTCGATTCCGGCCCCGGCCT
Gln	LACA	AGCCCCGTGGTGTAGT-GGTCC-AGCATGGCGGGTTTTGGCCCCGCCG--ACCCAGGTTCGAATCCTGGCGGGGCTA
	LBCA	AGCCCCGTGGTGTAAT-GGT---AGCACAACTGGTTTTGGTCCAGTTA--GCCCAGGTTCGAATCCTGGCGGGGCTG
Glu	LACA	GCCCCCGTAGTGTAGTCCGGTCAAGCATTTCGGGCTTTCACCCCGAAG-ACCCGGGTTCAAATCCCGGCGGGGGCA
	LBCA	GCTCCCATCGTCTAGC--GGTCTAGGACACTGGCCTCTCACGCCGGAA-ACACGGGTTCGAATCCCGTTGGGAGCA
Gly	LACA	GCGGGGGTAGTTTAGCCTGG--TAGGACATCAGCCTTCCAAGCTGAAGGTCGCGGGTTCAAATCCCGCCCCCCGCA
	LBCA	GCGGGAGTAGCTCAG-TTGG--TAGAGCATCAGCCTTCCAAGCTGAGGGTCGCGGGTTCAAATCCCGTCTCCCGCT
His	LACA	GTGGAAATAGTTCAGT---GGTT-AGAACATCAGATTGTGGCTCTGAAG--ACCCGGGTTCGATTCCCGGTTTCCACA
	LBCA	GTGGATATAGTTCAGTT--GGTT-AGAGCGCCAGATTGTGGCTCTGGAG-GTCGTGGGTTCAAGTCCCACTATCCACC
Ile	LACA	GGGCCCGTAGCTCAGCCT-GGTT-AGAGCGCTCGGCTGATAACCGAGAGGTCGGGGGTTCGAATCCCCCCGGGCCCA
	LBCA	GGGCCCGTAGCTCAGCCT-GGTT-AGAGCGCTCGGCTGATAACCGGGAGGTCGGGGGTTCAAATCCCCCCGGGCCCA
Leu	LACA	GCCGGGGTGGCCGAGTT-GGTCT-AAGGCGCTGGATTCAGGATCCAGTG-CCC--GTAA---------GG-CGCGTGGGTTCGAATCCCACCCCCGGCA
	LBCA	GCCGGGGTGGCCGAATT-GGTA--GAGGCGCTGGACTCAGGATCCAGTG-CCC--GTAA---------GG-CGTGCGGGTTCGAATCCCGCCCCCGGCA
Lys	LACA	GGGCCCGTAGCTCAGCCAGGT--AGAGCACCTGACTTTTAATCAGGTGGTCGAGGGTTCGAATCCCTCCGGGCCCG
	LBCA	GGGCCCGTAGCTCAGCC-GGT--AGAGCACCTGACTTTTAATCAGGTGGTCGTGGGTTCGAATCCCACCGGGCTCA
Met_e	LACA	GGCGGGGTAGCTCAGCC-GGTT-AGAGCGCCGGACTCATAATCCGGAGGTCGAGGGTTCGAATCCCTCCCCCGCCA
	LBCA	GGCGACGTAGCTCAGCT-GGTT-AGAGCGACGGAATCATAATCCGCAGGTCCGGGGTTCGAATCCCTGCGTCGCTA
Met_i	LACA	AGCGGGGTAGGGCAGCCT-GGT--AGCCCGCCGGGCTCATAACCCGGAGG-TCGGAGGTTCAAATCCTCCCCCCGCTA
	LBCA	CGCGGGGTAGAGCAGTCT-GGC--AGCTCGTCGGGCTCATAACCCGAAGG-TCGGAGGTTCAAATCCTCCCCCCGCTA
Phe	LACA	GCCGTGGTAGCTCAGTT-GGG-AGAGCGCCAGACTGAAGATCTGGATGTCGCTGGTTCGATTCCGGCCCACGGCA
	LBCA	GCCGGGGTAGCTCAGTT-GGT-AGAGCAGCGGACTGAAAATCCGCGTGTCGCTGGTTCGATTCCGGCCCCCGGCA
Pro	LACA	GGGGTCGTTGTCTAGCTTGGT--AGGATGCCAGCCTTGGGCGCTGGAGGTCGCTGGTTCGAATCCGGCCGACCCCA
	LBCA	CGGGGCGTAGCTCAGCCTGGT--AGAGCGCCTGCTTTGGGAGCAGGAGGTCGCCGGTTCAAATCCGGCCGCCCCGA
Ser	LACA	GCAGAGGTGGCTGAGC--GGTTC-AAGGCGCCGGTCTCGAAAACCGGTT-CCC-----GTAA------------GGGAACGTGGGTTCGAATCCCACCCTCTGCG
	LBCA	GGAGAGGTGGCTGAGT--GGTTG-AAGGCGCCGGTCTCGAAAACCGGTT-TCCC----GCAA-----------GGGA-ACGTGGGTTCGAATCCCACCCTCTCCG
Thr	LACA	GCCGCCGTAGCTCAGCT-GGC--AGAGCGGCTGACTTGTAATCAGCAG-GTCGCGGGTTCAAATCCCGCCGGCGGCT
	LBCA	GCCGGCGTAGCTCAGCT-GGC--AGAGCAACTGACTTGTAATCAGTAG-GTCGCGGGTTCAAATCCCGCCGCCGGCT
Trp	LACA	GGGCCCGTAGCTC-AGCCAGGT--AGAGCGGCGGGCTCCAGACCCGTAGGTCGGGGGTTCGAGTCCCTCCGGGCCCA
	LBCA	GGGTTCGTAGCTC-AGTT-GGT--AGAGCGGCGGTCTCCAAAACCGTAGGTCGGGGGTTCGAGTCCCTCCGGGCCCG
Tyr	LACA	CTCTCGGTAGCCAAGTT---GGTTTAAGGCGCCAGACTGTAAATCTGGAG------------ATCGGGCGTTCGACTCGCC
	LBCA	GGGTAGGTAGCCAAGT----GGTTAAAGGCGACAGACTGTAAATCTGTTGGC-CGT-C-GCCTTCGGGGGTTCGAATCCCT
Val	LACA	GGGTTGGTGGTCTAGTCCGGTT-ATGACACCTCCTTCACACGGAGGAG-GTCGGCGGTTCAAATCCGCCCCAACCCA
	LBCA	GGGTCGGTAGCTTAGCT-GGTC-AGAGCACCTCCTTTACACGGAGGGG-GTCGTAGGTTCGAGTCCTATCCGGCCCA

Supplementary Figure S3. Comparison of maximum probability at each site of LUCA tRNAs. Sites with maximum probability < 0.9 for any state are shown in yellow, and sites with maximum probability < 0.5 for any state are shown in red. The sequences were inferred based on the trees shown in Fig. 3B. For tRNA[Tyr], the form with long extra arm is shown.

Site	1	2	3	4	5	6	7	8	9	10	11	12	13	14	15	16	17	18	19	20	21	22	23	24	25	26	27	28	29	30	31	32	33	34	35	36	37	38	39	40	41	42	43	44	45	46	47	48	49	50	51	52	53	54	55	56	57	58	59	60	61	62	63	64	65	66	67	68	69	70	71	72	73	74	75	76	77	78	79	80	81	82	83	84	85	86	87
Ala_Anc	G	G	G	C	C	G	G	T	A	G	C	T	C	A	G	C	C	-	G	G	A	-	-	A	G	A	G	C	G	C	C	C	G	C	T	T	T	G	C	A	C	G	C	G	G	G	A	G	G	-	-	-	-	-	-	-	-	-	-	-	T	C	C	C	G	G	G	T	T	C	A	A	A	T	C	C	C	G	G	C	C	G	G	T	C	C	A
Arg_Abc	G	G	G	C	C	C	G	T	A	G	C	T	C	A	G	C	T	-	G	G	A	T	-	A	G	A	G	C	A	A	C	G	G	A	C	T	T	C	T	A	A	T	C	C	G	T	A	G	G	-	-	-	-	-	-	-	-	-	-	-	T	C	G	G	G	G	G	T	T	C	G	A	A	T	C	C	C	C	C	C	G	G	G	C	C	C	G
Asn_Anc	G	C	C	G	C	C	G	T	A	G	C	T	C	A	G	T	T	-	G	G	T	-	-	A	G	A	G	C	A	G	C	C	G	G	C	T	G	T	T	A	A	C	C	G	G	T	A	G	G	-	-	-	-	-	-	-	-	-	-	-	T	C	G	C	A	G	G	T	T	C	G	A	G	T	C	C	T	G	C	C	G	G	C	G	G	C	G
Asp_Anc	G	G	T	C	C	G	G	T	A	G	T	G	T	A	G	T	-	-	G	G	T	T	-	A	G	C	A	T	G	C	C	T	G	C	C	T	G	T	C	A	C	G	C	A	G	G	A	G	-	-	-	-	-	-	-	-	-	-	-	-	G	T	G	C	G	G	G	T	T	C	A	A	G	T	C	C	C	G	T	C	C	G	G	A	C	C	G
Cys_Anc	G	G	C	C	G	G	G	T	G	G	C	A	G	A	G	T	-	-	G	G	T	T	-	-	A	T	G	C	A	G	C	G	G	A	C	T	G	C	A	A	A	T	C	C	G	C	G	T	T	-	-	-	-	-	-	-	-	-	-	-	A	C	G	C	C	G	G	T	T	C	G	A	T	T	C	C	G	G	C	C	C	C	G	G	C	C	T
Gln_Anc	A	G	C	C	C	C	G	T	G	G	T	G	T	A	G	T	-	-	G	G	T	C	C	A	G	C	A	C	G	G	C	G	G	G	T	T	T	T	G	G	C	C	C	A	G	C	C	G	-	-	-	-	-	-	-	-	-	-	-	-	A	C	C	C	A	G	G	T	T	C	G	A	A	T	C	C	T	G	G	C	G	G	G	G	C	T	A
Glu_Anc	G	C	T	C	C	C	A	T	C	G	T	C	T	A	G	C	-	-	G	G	T	C	T	A	G	G	A	C	A	C	T	G	G	C	C	T	C	T	C	A	C	G	C	C	G	G	A	A	-	-	-	-	-	-	-	-	-	-	-	-	A	C	A	C	G	G	G	T	T	C	G	A	A	T	C	C	C	G	T	T	G	G	G	A	G	C	A
Gly_Anc	G	C	G	G	G	G	G	T	A	G	C	T	C	A	G	T	T	-	G	G	T	-	-	A	G	A	G	C	A	T	C	A	G	C	C	T	T	C	C	A	A	G	C	T	G	A	A	G	G	-	-	-	-	-	-	-	-	-	-	-	T	C	G	C	G	G	G	T	T	C	A	A	A	T	C	C	C	G	C	C	C	C	C	C	G	C	A
His_Anc	G	T	G	G	A	A	A	T	A	G	T	T	C	A	G	T	T	-	G	G	T	T	-	A	G	A	G	C	A	T	C	A	G	A	T	T	G	T	G	G	C	T	C	T	G	A	A	G	G	-	-	-	-	-	-	-	-	-	-	-	T	C	G	C	G	G	G	T	T	C	A	A	G	T	C	C	C	G	C	T	T	T	C	C	A	C	A
Ile_Anc	G	G	G	C	C	C	G	T	A	G	C	T	C	A	G	C	C	T	G	G	T	T	-	A	G	A	G	C	G	C	T	C	G	G	C	T	G	A	T	A	A	C	C	G	G	G	A	G	G	-	-	-	-	-	-	-	-	-	-	-	T	C	G	G	G	G	G	T	T	C	A	A	A	T	C	C	C	C	C	C	G	G	G	C	C	C	A
Leu_Anc	G	C	C	G	G	G	G	T	G	G	C	C	G	A	G	T	T	-	G	G	T	C	T	A	A	G	G	C	G	C	T	G	G	A	C	T	C	A	G	G	A	T	C	C	A	G	T	G	-	C	C	C	G	T	A	A	G	G	-	C	G	C	G	T	G	G	G	T	T	C	G	A	A	T	C	C	C	A	C	C	C	C	C	G	G	C	A
Lys_Anc	G	G	G	C	C	C	G	T	A	G	C	T	C	A	G	C	C	-	G	G	T	-	-	A	G	A	G	C	A	C	C	T	G	A	C	T	T	T	T	A	A	T	C	A	G	G	T	G	G	-	-	-	-	-	-	-	-	-	-	-	T	C	G	A	G	G	G	T	T	C	G	A	A	T	C	C	C	T	C	C	G	G	G	C	C	C	A
MetE_Anc	G	G	C	G	G	G	G	T	A	G	C	T	C	A	G	C	C	-	G	G	T	T	-	A	G	A	G	C	G	C	C	G	G	A	C	T	C	A	T	A	A	T	C	C	G	G	A	G	G	-	-	-	-	-	-	-	-	-	-	-	T	C	G	A	G	G	G	T	T	C	G	A	A	T	C	C	C	T	C	C	C	C	C	G	C	C	A
MetI_Anc	A	G	C	G	G	G	G	T	A	G	G	G	C	A	G	C	C	T	G	G	T	-	-	A	G	C	C	C	G	C	C	G	G	G	C	T	C	A	T	A	A	C	C	C	G	G	A	G	G	-	-	-	-	-	-	-	-	-	-	-	T	C	G	G	A	G	G	T	T	C	A	A	A	T	C	C	T	C	C	C	C	C	C	G	C	T	A
Phe_Anc	G	C	C	G	G	G	G	T	A	G	C	T	C	A	G	T	T	-	G	G	T	-	-	A	G	A	G	C	A	G	C	G	G	A	C	T	G	A	A	A	A	T	C	C	G	C	G	T	G	-	-	-	-	-	-	-	-	-	-	-	T	C	G	C	T	G	G	T	T	C	G	A	T	T	C	C	G	G	C	C	C	C	C	G	G	C	A
Pro_Anc	G	G	G	G	G	C	G	T	A	G	C	T	C	A	G	C	C	T	G	G	T	-	-	A	G	A	G	C	G	C	C	T	G	C	C	T	T	G	G	G	A	G	C	A	G	G	A	G	G	-	-	-	-	-	-	-	-	-	-	-	T	C	G	C	C	G	G	T	T	C	A	A	A	T	C	C	G	G	C	C	G	C	C	C	C	C	A
Ser_Anc	G	C	A	G	A	G	G	T	G	G	C	T	G	A	G	C	-	-	G	G	T	T	C	A	A	G	G	C	G	C	C	G	G	T	C	T	C	G	A	A	A	A	C	C	G	G	T	T	T	C	C	C	G	T	A	A	G	G	G	A	A	C	G	T	G	G	G	T	T	C	G	A	A	T	C	C	C	A	C	C	C	T	C	T	G	C	G
ThrAnc	G	C	C	G	C	C	G	T	A	G	C	T	C	A	G	C	T	-	G	G	C	-	-	A	G	A	G	C	A	G	C	T	G	A	C	T	T	G	T	A	A	T	C	A	G	T	A	G	G	-	-	-	-	-	-	-	-	-	-	-	T	C	G	C	G	G	G	T	T	C	A	A	A	T	C	C	C	G	C	C	G	G	C	G	G	C	T
Trp_Anc	G	G	G	C	C	C	G	T	A	G	C	T	C	A	G	C	T	-	G	G	T	-	-	A	G	A	G	C	G	G	C	G	G	A	C	T	C	C	A	A	A	T	C	C	G	T	A	G	G	-	-	-	-	-	-	-	-	-	-	-	T	C	G	G	G	G	G	T	T	C	G	A	G	T	C	C	C	T	C	C	G	G	G	C	C	C	A
Tyr_Anc_long	G	G	G	T	C	G	G	T	A	G	C	C	A	A	G	T	T	-	G	G	T	T	A	A	A	G	G	C	G	A	C	A	G	A	C	T	G	T	A	A	A	T	C	T	G	T	A	G	G	C	C	-	G	T	C	-	G	C	C	T	T	C	G	G	G	G	G	T	T	C	G	A	A	T	C	C	C	T	C	C	C	G	G	C	C	C	A
Val_Abc	G	G	G	T	C	G	G	T	A	G	C	T	T	A	G	C	C	-	G	G	T	T	-	A	G	A	G	C	A	C	C	T	C	C	T	T	T	A	C	A	C	G	G	A	G	G	A	G	G	-	-	-	-	-	-	-	-	-	-	-	T	C	G	T	G	G	G	T	T	C	A	A	A	T	C	C	C	A	T	C	C	G	G	C	C	C	A

Supplementary Figure S4. Cloverleaf structures of LUCA tRNAs. Numbering system of each nucleotide in tRNA is according to Giegé & Eriani (Giegé and Eriani 2023). The degree of identity of each nucleotide in the ancestral tRNA sequence with the corresponding nucleotide in the *E. coli* tRNA genes is shown in stages from deep red to white. Number of tRNA gene in *E. coli* genome (NC_000913) is as follows. One: $tRNA^{Cys}$s, $tRNA^{His}$, and $tRNA^{Trp}$, two: $tRNA_e^{Met}$ and $tRNA^{Phe}$, three: $tRNA^{Asp}$, $tRNA^{Pro}$, and $tRNA^{Tyr}$, four: $tRNA^{Asn}$, $tRNA^{Gln}$, $tRNA^{Glu}$, $tRNA_i^{fMet}$, and $tRNA^{Thr}$, five: $tRNA^{Ala}$, $tRNA^{Ile}$, and $tRNA^{Ser}$, six: $tRNA^{Gly}$ and $tRNA^{Lys}$, seven: $tRNA^{Arg}$ and $tRNA^{Val}$, eight: $tRNA^{Leu}$.

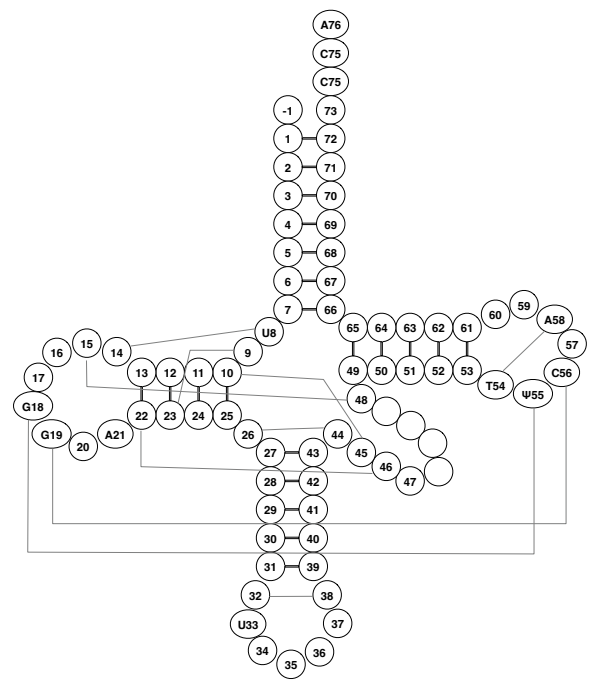

Portion of different residues of E. coli tRNAs from Commonote tRNA	
0	G
0 < x ≤ 0.25	G
0.25 < x ≤ 0.5	G
0.5 < x ≤ 0.75	G
0.75 < x < 1	G
1	G

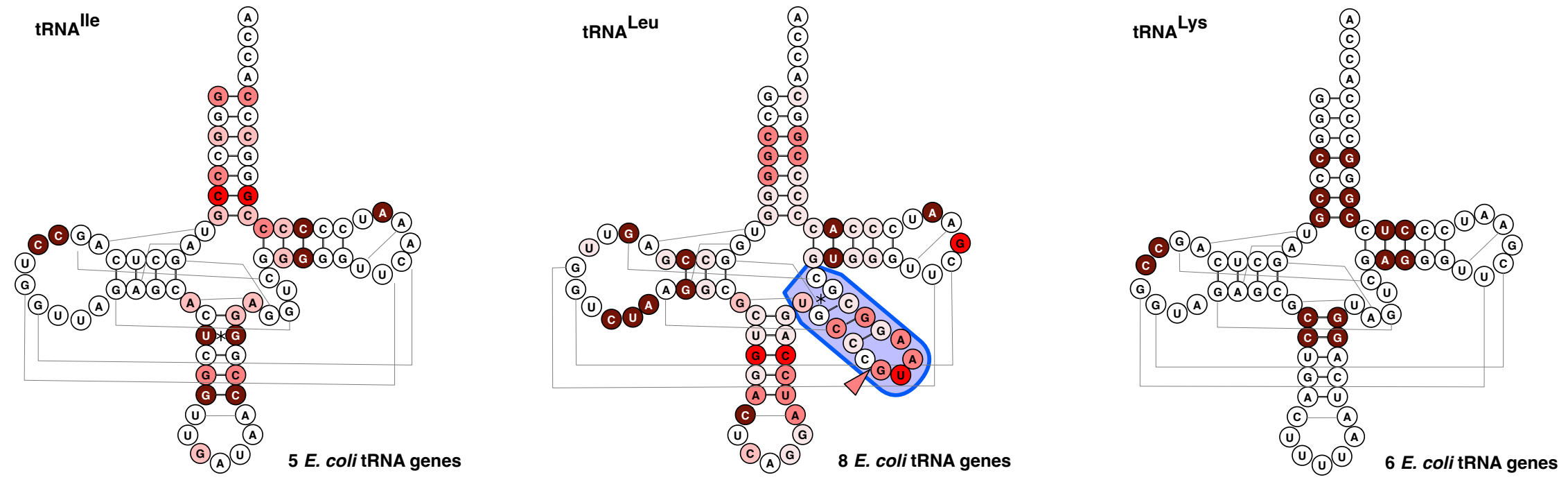

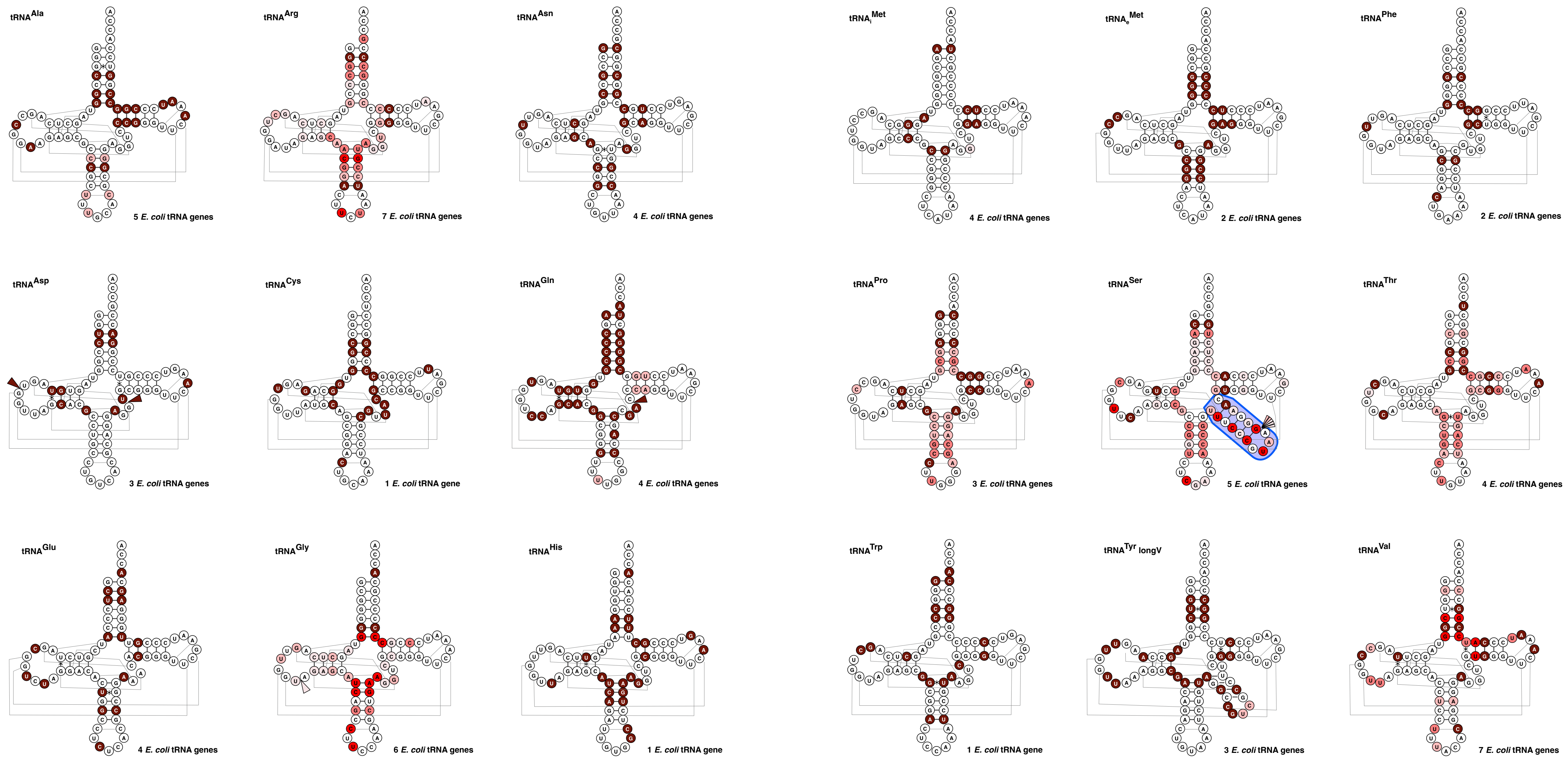

Supplementary Figure S5. 7M urea 15% acrylamide gel electrophoresis of *in vitro* transcripts of tRNAs of mutants of *E. coli* and LUCA $tRNA^{Ala}$ and $tRNA^{Ser}$. Abbreviations. Eco: *E. coli*, Anc: Ancestral (LUCA).

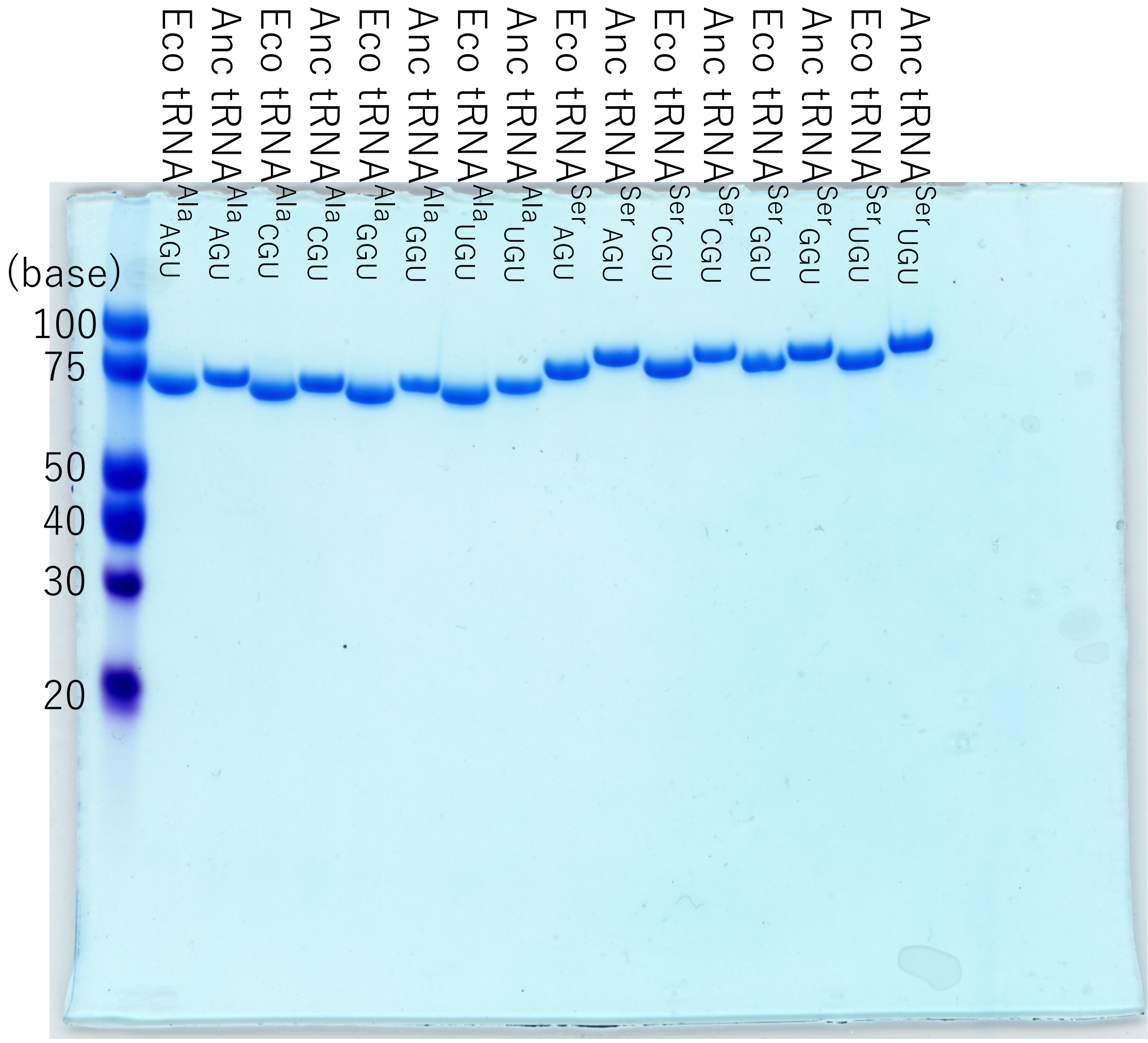

Supplementary Figure S6. LUM values measured by the HiBiT system for cell-free protein synthesis reaction solutions with test codon ACN and *E. coli* $tRNA^{Ser}_{NGU}$ in (A) and LUCA $tRNA^{Ser}_{NGU}$ in (C), expressed as a percentage with the LUM value of the positive and negative controls as 1 and 0, respectively. However, the data was obtained from only one measurement. Based on the results of (A) and (C), the translation efficiency of the third letter of codon by the first anticodon letter of *E. coli* $tRNA^{Ser}$ was evaluated in (B), and the translation efficiency of the third letter of codon by the first anticodon letter of LUCA $tRNA^{Ser}$ was evaluated in (D) based on the criteria in (E). Since the efficiency of translation of the third letter of the codon by the first anticodon letter of $tRNA^{Ser}$ in this experiment is lower than that of $tRNA^{Ala}$, the evaluation criterion in (E) was set lower than that in Figure 7 (E).

A

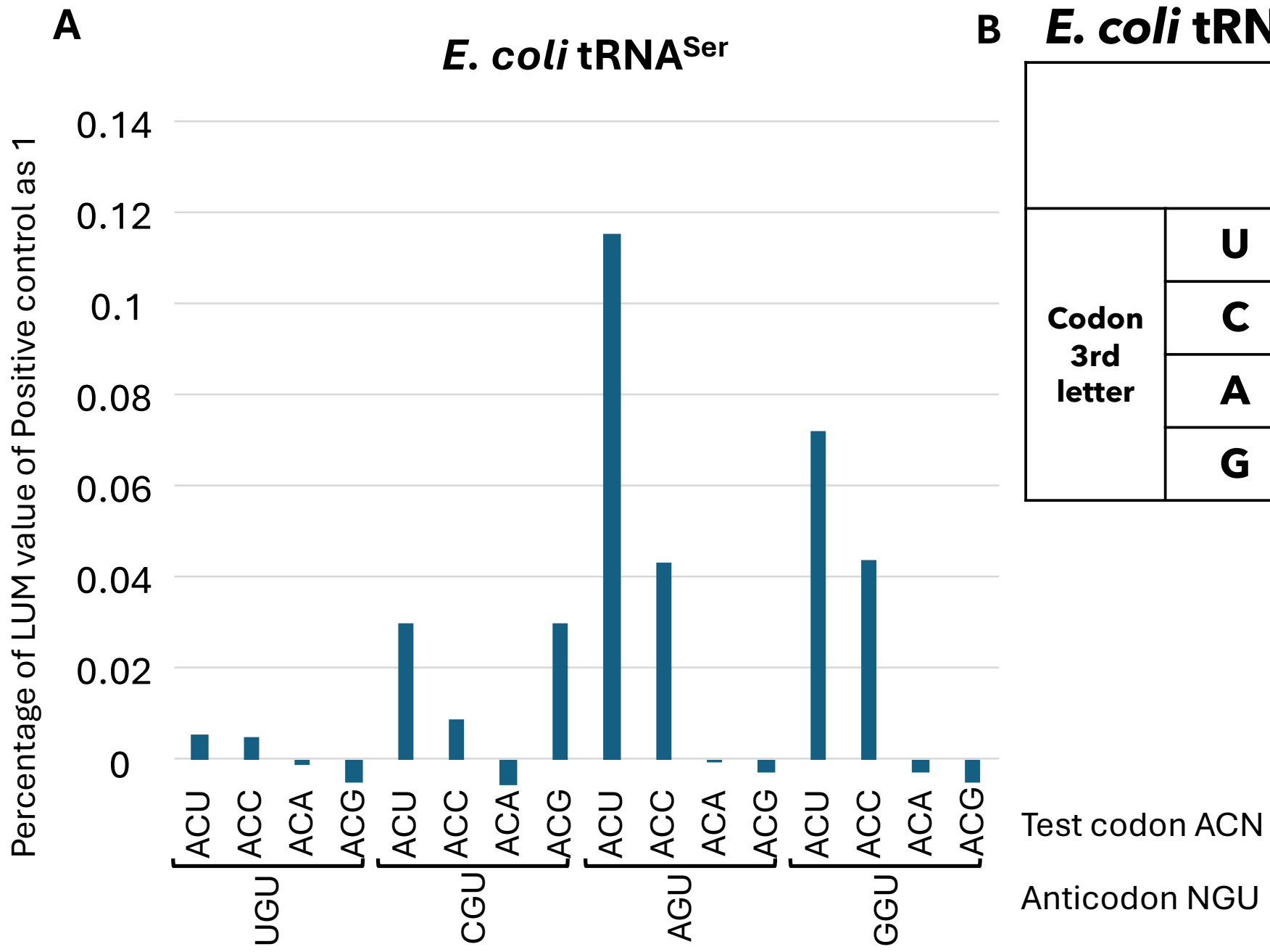

B ***E. coli* $tRNA^{Ser}$**

		Anticodon 1st letter			
		U	C	A	G
Codon 3rd letter	U	+	++	+++	+++
	C	±	+	++	++
	A	-	-	-	-
	G	-	++	-	-

C

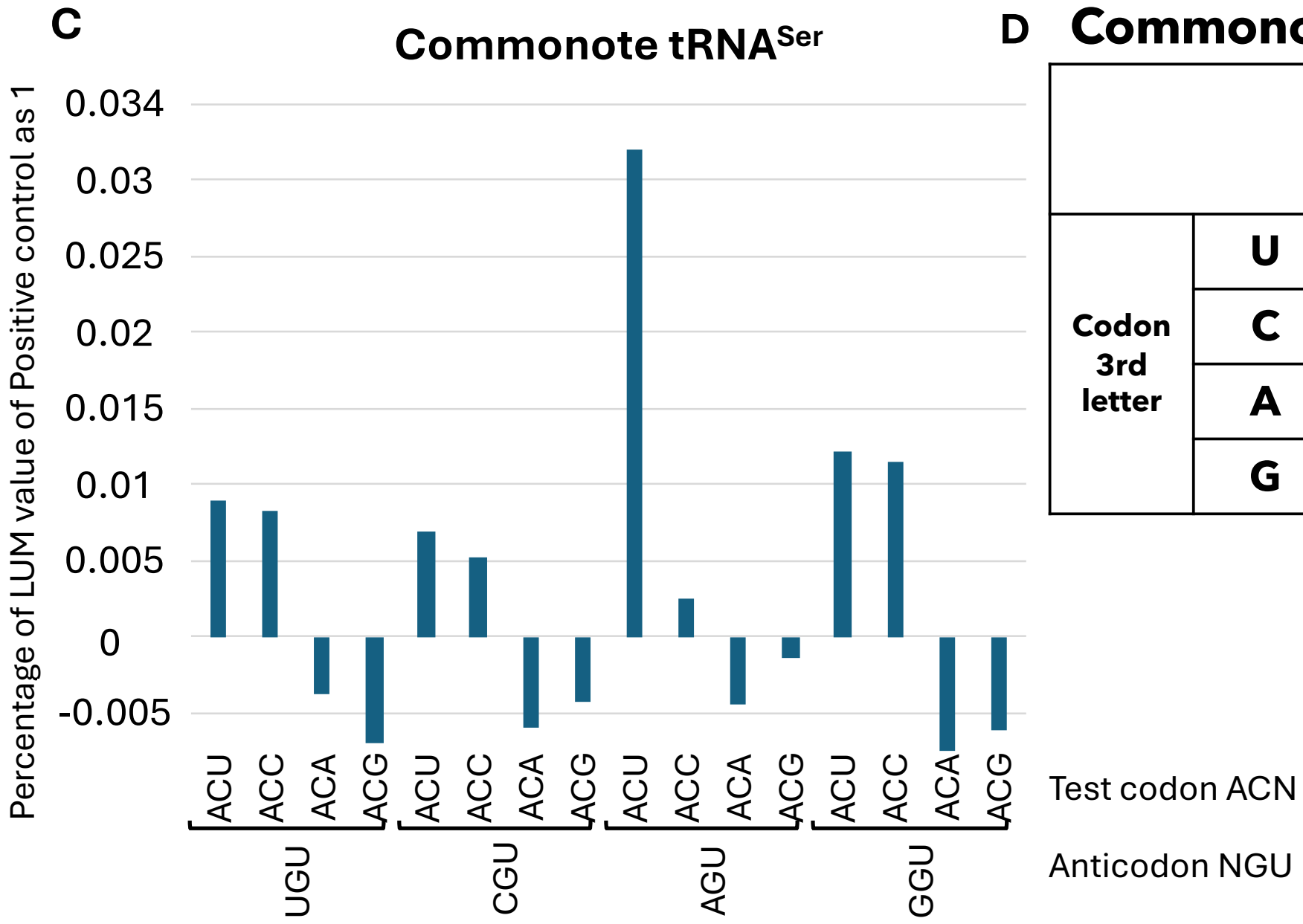

D **Commonote $tRNA^{Ser}$**

		Anticodon 1st letter			
		U	C	A	G
Codon 3rd letter	U	+	+	++	+
	C	+	+	±	+
	A	-	-	-	-
	G	-	-	-	-

E

+++: ≧ 0.05
++: ≧ 0.025
+: ≧ 0.005
±: ≧ 0.0025
–: < 0.0025
